# Sodium butyrate mediates histone crotonylation and alleviated neonatal rats hypoxic–ischemic brain injury through gut–brain axis

**DOI:** 10.3389/fmicb.2022.993146

**Published:** 2022-10-20

**Authors:** Xuejia He, Ting Zhang, Yubing Zeng, Pei Pei, Yulan Liu, Wenbin Jia, Hongyang Zhao, Meirong Bi, Shan Wang

**Affiliations:** ^1^Beijing Municipal Key Laboratory of Child Development and Nutriomics, Capital Institute of Pediatrics-Peking University Teaching Hospital, Beijing, China; ^2^Beijing Municipal Key Laboratory of Child Development and Nutriomics, Capital Institute of Pediatrics, Beijing, China; ^3^Department of Pediatrics, Central Hospital Affiliated to Shandong First Medical University, Jinan, China; ^4^Department of Pharmacology, Graduate School of Pharmaceutical Sciences, Tohoku University, Sendai, Japan

**Keywords:** neonatal hypoxic–ischemic encephalopathy, hypoxic–ischemic brain damage, gut microbiota, sodium butyrate, histone crotonylation

## Abstract

Neonatal hypoxic–ischemic encephalopathy (HIE) refers to nervous system damage caused by perinatal hypoxia, which is the major cause of long-term neuro-developmental disorders in surviving infants. However, the mechanisms still require further investigation. In this study, we found that the butanoate metabolism pathway exhibited significantly decreased and short chain fatty acid (SCFAs)-producing bacteria, especially butyrate-producing bacteria, were significantly decreased in fecal of neonatal hypoxic–ischemic brain damage (HIBD) rats. Surprisingly, Sodium butyrate (SB) treatment could ameliorate pathological damage both in the cerebral cortex and hippocampus and facilitate recovery of SCFAs-producing bacteria related to metabolic pathways in neonatal HIBD rats. Moreover, we found that in samples from SB treatment neonatal HIBD rats cortex with high levels of butyrate acid along with aberrant key crotonyl-CoA-producing enzymes ACADS levels were observed compared HIBD rats. We also demonstrated that a decrease in histone 3-lysine 9-crotonylation (H3K9cr) downregulated expression of the HIE-related neurotrophic genes *Bdnf*, *Gdnf*, *Cdnf*, and *Manf* in HIBD rats. Furthermore, SB restored H3K9cr binding to HIE-related neurotrophic genes. Collectively, our results indicate that SB contributes to ameliorate pathology of HIBD by altering gut microbiota and brain SCFAs levels subsequently affecting histone crotonylation-mediated neurotrophic-related genes expression. This may be a novel microbiological approach for preventing and treating HIE.

## Introduction

Neonatal hypoxic–ischemic encephalopathy (HIE) refers to nervous system damage caused by perinatal hypoxia, which is the major cause of long-term neuro-developmental disorders in surviving infants (Wang and Jia, 2021). When a fetus is subjected to hypoxia during the perinatal period, dysfunction and metabolic disorders occur in multiple organs, such as the brain and gut (Watkins et al., 2017). Indeed, many bidirectional interactions occur between the central and enteric nervous systems in the human body through metabolic, immune, and other pathways (Koh et al., 2016; Cryan et al., 2019). This microbiota–gut–brain axis plays a key role in regulating homeostasis in the human body (Cryan et al., 2020).

The gut microbiota is a part of the gut–brain axis, and the establishment and functional maturation of the key microbiome is crucial in early life (Gala et al., 2022). Studies have shown that the microbiome plays an important, region-specific role in brain development as early as hours after birth (Castillo-Ruiz et al., 2018). Some studies demonstrated that dysbiosis of the gut microbiome early in life is associated with specific childhood or adult diseases, such as asthma, diabetes, allergic diseases, and autism spectrum disorder (Arrieta et al., 2015; Al-Jameel, 2021). Studies have reported that fecal microbiota transplantation from stroke model mice increases the severity of ischemic brain damage in germ-free mice (Singh et al., 2016). However, administration of a specific bacterial strain, *Clostridium butyricum*, has a neuroprotective effect in a cerebral ischemia–reperfusion injury mouse model (Sun et al., 2016b). This evidence demonstrates a complex interplay between the gut microbiota and its role in the development of nervous system function in an animal model.

Accumulating evidence has shown that the metabolomic profiles of fecal and plasma samples significantly differ between newborns with HIE and age-matched healthy control groups (Sanchez-Illana et al., 2017; Shibasaki et al., 2018; O'Boyle et al., 2021). Among the microbial metabolites involved in the gut–brain axis, short chain fatty acids (SCFAs) are probably the most fascinating. Specific gut microbiota break down dietary fiber into SCFAs, such as acetate, propionate, and butyrate, which are energy sources and are also involved in regulation of the gut epithelial barrier (Ratajczak et al., 2019). SCFAs also promote blood brain barrier (BBB) integrity through regulation of the tight junction protein occludin (Braniste, 2014).

Butyrate is a microbiota produced SCFAs, its profound effect on epigenetic modulation is not limited to gut (Lee, 2019; Noureldein et al., 2020). Protein lysine acylation, such as acetylation, crotonylation, and butyrylation, is a common post-translational protein modification (Fellows et al., 2018). Lysine acylation is modulated by “writers” (transferases that catalyze the modification), “erasers” (enzymes that remove the modification), and small molecular acyl-coenzyme As (CoAs), which are sourced from intermediate metabolites from different metabolic pathways and the gut microbiota (Sabari et al., 2017; Sharma and Rando, 2017). Growing evidence has demonstrated that butyrate can promote histone crotonylation. One line of evidence indicates that it inhibits the decrotonylase activity of histone deacetylases (Fellows et al., 2018). Furthermore, another critical line of evidence indicates that butyrate can be converted to butyryl-CoA and further into crotonyl-CoA, which is a precursor of histone crotonylation (Li, 2022; Park et al., 2022). Microbiota-derived butyrate can affect the brain epigenetic machinery after being absorbed from the gut and crossing the BBB (Minamiyama et al., 2004). Concentrations of butyrate in wet-brain samples ranged from 0.4 to 0.7 μmol/g and were approximately an order of magnitude higher than those reported in peripheral blood (Stilling et al., 2016). One study revealed that the butyric acid level could be slightly elevated in brains through supplementation with live *Clostridium butyricum* in a mouse model of brain ischemia (Sun et al., 2016a). Taken together, this evidence supports that butyrate is crucial in regulating brain and gut communication (Cryan et al., 2020).

In this study, we found that the gut microbiota composition and metabolic phenotype obviously differed in hypoxic–ischemic rats. The butanoate metabolism pathway exhibited significantly decreased enrichment, and SCFAs-producing bacteria, especially butyrate-producing bacteria, were significantly decreased in the neonatal hypoxic–ischemic brain damage (HIBD) model rats faeces. Surprisingly, sodium butyrate (SB) treatment could ameliorate pathological brain injury and behavioral function in neonatal HIBD rats and facilitate recovery of SCFAs-producing bacteria and the related metabolic pathways. Moreover, in samples from HIBD neonatal rats cortex with high levels of butyrate acid along with aberrant key crotonyl-CoA-producing enzymes ACADS levels were observed under SB treatment. Furthermore, we demonstrated that a decrease in histone 3-lysine 9-crotonylation (H3K9cr) downregulated expression of the HIE-related neurotrophic genes. Our findings comprise the first demonstration that regulation of the H3K9cr level at gene promoters alleviates brain damage in hypoxic–ischemic rats, which will increase the understanding of the relationship between the gut microbiota and the neonatal brain. The gut microbiome may be recognized as a key factor that improves outcomes of HIE infants in the near future.

## Materials and methods

### Animals

Wistar rats, 8–10 weeks old, SPF grade, provided by the Central Hospital Affiliated to Shandong First Medical University Laboratory and housed in a 12-h light/dark cycle, with free access to food and water. One hundred and forty P7-P10 rats pups were randomly divided into the following groups: Sham (*n* = 30), HIBD (*n* = 50), HIBD + SB group (*n* = 50) and HIBD + SAL group (*n* = 10). The Ethics Committee of Central Hospital Affiliated to Shandong First Medical University, Shandong, China, approved all protocols used in this study (JNCH2021-58), and procedures involving animal handling comply with institutional guidelines for the care of laboratory animals.

### HIBD model

The hypoxic–ischemic model was referenced by the classical Rice–Vannucci method (Rice et al., 1981). A total of P7-P10 rats’ pups, weighing 13–16 g was used and anesthetized with chloral hydrate (200 mg/kg). Subsequently, the left common carotid artery was exposed and permanently ligate using sutures. After the wound suturing and the blood drying, the rats’ pups were returned to their mother. After awakening, the pups were placed in a closed chamber containing 8% O2 and 92% N2 for 2 h. The Sham group pups underwent artery exposure, but without any ligation and hypoxia. Pups were kept in a warm condition to remain body temperature through all processes.

### TTC staining

The brain samples cerebral infarction were detected with TTC staining to evaluate the success of animal modeling, after 24 h of HI. The TTC powder (Sigma-T8877) was prepared into 1% TTC solution with phosphate buffered saline (PBS). Pups were deeply anesthetized and quickly removed the brains. Then, the brain samples were frozen in a refrigerator at −20°C for 10 min and were cut in coronal position to 5 brain sections with a thickness of 2–3 mm. The brain slices were placed successively into a 12-well plate with 1%TTC solution, shielded from light, placed in a 37°C-water bath for about 10 min, and photographed.

### SB treatment of rats

The pups were treated intragastrically with SB (100 mg/kg, Sigma-303410) at doses of 100 μl once daily for successive 2 weeks. The Sham, HIBD and HIBD + SAL groups rats were treated with SAL at doses of 100 μl as the vehicle control.

### Morris water maze test

After 2 weeks of SB treatment, four groups of rats pups were tested for learning and memory in a Morris water maze. From day 1 to day 5, the pups entered the water from different quadrants at the same time every day. The pups that found the underwater platform within 60 s were allowed to stay on the platform for 5 s. If they fail to find the platform, it is gently guided to the platform after 60 s and remains there for 15 s. The time it takes to find the platform is called escape latency. On day 6, the platform was taken out of the water and each pup was given an exploratory test. Record the time they spent in the original platform location and the number of times they crossed the platform.

### Fecal samples harvest

After the last day of SB treatment, the four groups rats were deeply anesthetized to collect fresh feces from colon and rectum. Then put into new sterile tubes and stored at −80°C until further use.

### Tissue harvest

Briefly, after deeply anesthetized with chloral hydrate (200 mg/kg), the rats pups in Sham, HIBD, HIBD + SAL and HIBD + SB groups were perfused with physiological saline from the right auricle until the liver turns white. The brain of rats was removed on ice, put into an ice-cold PBS and rapidly separated the infarct side cerebral cortex (including periinfarct tissue) and hippocampus, snap-frozen in liquid nitrogen, and then stored at −80°C for following molecular experiments. Meanwhile, the brains were collected and stored in the 4% paraformaldehyde for following Immunohistochemical (IHC) and Hematoxylin and Eosin (HE) staining.

### HE staining

Brain tissue stored in the 4% paraformaldehyde were stained with hematoxylin and eosin (HE) and histopathological changes of brain tissue were observed using light microscopy.

### Immunohistochemical staining

Brain tissue stored in the 4% paraformaldehyde were performed IHC staining for H3K9cr and ACADS. Incubation with primary antibodies against H3K9cr (1:500 dilution; PTM-516RM) and ACADS (1,200; Ab156571). The intensity of the positive area was analyzed by Image J software.

### Western blot analysis

Histones were extracted from 100 mg cerebral cortex tissue using EpiQuik Total Histone Extraction Kit (Epigentek, USA). Western blot experiments were performed as previously described (Jia et al., 2021). Incubation with primary antibodies Anti-crotonyllysine antibody (PTM-501, 1:2000 dilution), Anti-acetyllysine antibody (PTM-105, 1:2000 dilution), anti-H3K18cr (PTM-517, 1:2000 dilution), and anti-H3K9cr (PTM-516RM, 1:2000 dilution) and secondary antibody (ab205718, 1:5000 dilution).

### 16S rRNA gene sequencing

For this sequencing, Sham, HIBD and HIBD + SB groups contain 5, 5 and 8 samples, respectively. Genomic DNA of fecal samples were extracted by using QIAamp Power Fecal QIAcube HT kit (QIAGEN-51531). Bacterial DNA was amplified with the primers targeting V3–V4 regions (5′-TACGGRAGGCAGCAG-3′, 5′-GGGTATCTAATCCT-3′). Then DNA was sequenced using MiSeq PE300 platform (Illumina, California, USA). After generating high-quality sequences from the sequencing data (reads with ambiguous, homologous sequences or below 200 bp were abandoned. Reads with 75% of bases above Q20 were retained. Then, reads with chimera were detected and removed. These two steps were achieved using QIIME software1.8.0). Clean reads were subjected to primer sequences removal and clustering to generate OTUs using Vsearch software with 97% similarity cutoff. QIIME package was used to pick out representative reads of each OTU, and all representative sequences were compared and annotated with the database. Greengenes database was used for comparison, and RDP classifier software was used for annotation of species comparison.

### Metabolomic analysis

For non-targeted metabolite analysis, every group contains 6 samples. The 60 mg fecal samples was weighed and placed into a 1.5 ml centrifuge tube with 40 μl of internal standard (L-2-chloro-phenylalanine, 0.3 mg/ml, methanol) for gas chromatography–mass spectrometry (GC/MS) preprocessing. Analysis of the samples was performed on an Agilent 7890B gas chromatography system and an Agilent 5977A MSD system (Agilent Technologies, USA). The injector temperature was maintained at 260°C. Injection volume was 1ul by splitless mode. The collision energy was 70 eV. Mass spectrometric data was acquired in a full-scan mode (*m*/*z* 50–500). The data were pretreated using BaseFileConverter software. Metabolites were annotated through LUG database (Untarget database of GC–MS from Lumingbio).

For targeted metabolic analysis, every group contains 6 samples. UPLC-ESI-MS/MS analysis method was used for qualitative and quantitative detection of SCFAs. The 30 mg brain samples were weighed and added 300 μl 50% acetonitrile-water solution (V/V), ultrasound in ice water bath for 10 min, centrifuge (10 min, 4°C, 12,000 RPM), take 80 μl supernatant transfer to sample vial. Derivatization of samples and standards: add 40 μl 200 mM 3-nph (50% acetonitrile-aqueous solution, v/v) and 40 μl 120 mM edc-6% pyridine (50% acetonitrile-aqueous solution, v/v) into the injection glass filled with extraction solution, and react at 40°C for 30 min. After cooling on ice for 1 min, 150 μl of supernatant was absorbed with a syringe, filtered by a 0.22 μm organic phase pinhole filter, and transferred to a brown injection bottle, stored at −80°C until machine analysis. Metabolite quantification was analyzed using MRM mode of triple quadrupole mass spectrometry. Mass spectrometry data of different samples were obtained and peak area integration was performed for all chromatographic peaks. The peak area of each chromatographic peak represents the relative content of corresponding metabolites.

### RNA-seq analyses

After the homogenate of the tissues，total RNA was isolated using TRIzol Reagent (Invitrogen, USA) and purified by mRNA enrichment according to the manufacturer’s protocol. The cDNA libraries were constructed for each pooledd sample according to the manufacturer’s instructions. Base Calling of Illumina high-throughput sequencing raw files and conversion to Sequenced Reads. Original sequenced reads were filtered and analyzed after quality control, including reference genome alignment, sample correlation analysis, expression level analysis, differential gene analysis and GO enrichment analysis.

### Real-time quantitative polymerase chain reaction

After the homogenate of the tissues, total RNA was extracted using the Trizol reagent (Invitrogen, USA). The RNA to cDNA reverse transcription was performed using Revert Aid First Strand cDNA Synthesis Kit (ABM, Canada). Maxima SYBR Green/ROX qPCR Master Mix (ABM, Canada) was used for RT-qPCR. The threshold cycle of each sample was recorded, and data were analyzed by normalization to GAPDH values using the 2^−△△Ct^ method. The primer sequences were listed in Additional: Supplementary Table S8.

### ChIP-seq

Fifty milligram cerebral cortex tissue was as ChIP samples. ChIP was done with Simple ChIP Plus Enzymatic Chromatin IP Kit (Magnetic Beads; CST-9005S) following the manufacturer’s protocols. The immunoprecipitations used antiH3K9cr (PTM-516RM, 1:50) and normal Rabbit IgG is used as negative controls. Before CHIP-sequencing, sample fragments have to be 200–500 bp qualified by Agilent2200. Generate Illumina sequencing libraries from the NEBNext^®^Ultra™DNA Library Preparation Kit (New England Biolabs) manufacturer’s Manual. The library quality was examined using an Agilent 2100 bioanalyzer followed by high-throughput 150-base pair end sequencing on the Illumina Novaseq 6000 sequencer. Then these clean reads were aligned to rat reference genome (UCSC rn5) using bowtie2 software (v2.2.4) with default parameters. Peak calling was performed with MACS software (v2.2.7.1). Differentially enriched regions were identified by diffReps software (v1.55.4). Then, the enriched peaks were annotated with the UCSC RefSeq database, and the peak information was connected with gene annotations.

### ChIP qPCR

ChIP assays and chromatin prepared as mentioned above, then the ChIPed DNA was analyzed by qPCR, which were performed using QuantStudio 7 Flex with SYBR Green detection. The ChIP qPCR primer sequences were listed in Additional: Supplementary Table S9. The calculation formula is percent input = 2% × 2^(CT) 2% input sample − (CT) IP sample^.

### Statistical analysis

Data were analyzed using GraphPad Prism (Version 9.2.0) and SPSS 20.0 (IBM, USA). Data were presented as means ± SD. Student’s *t* test or ANOVA analysis was used for group comparisons. *p* values <0.05 were considered indicative of a statistically significant difference. Pearson’s correlation analysis was used to analyze the association between gut microbiota and metabolites.

## Results

To explore a higher dimension of evidence, we performed a multi-omics analysis of brain tissue and gut fecal material from the Sham, HIBD, and HIBD + SB groups, including metabolomic, microbiome, transcriptomic, and epigenomic analyses.

### Reduced butyrate metabolic pathway in fecal metabolite profiles of HIBD rats

**Figure 1:**
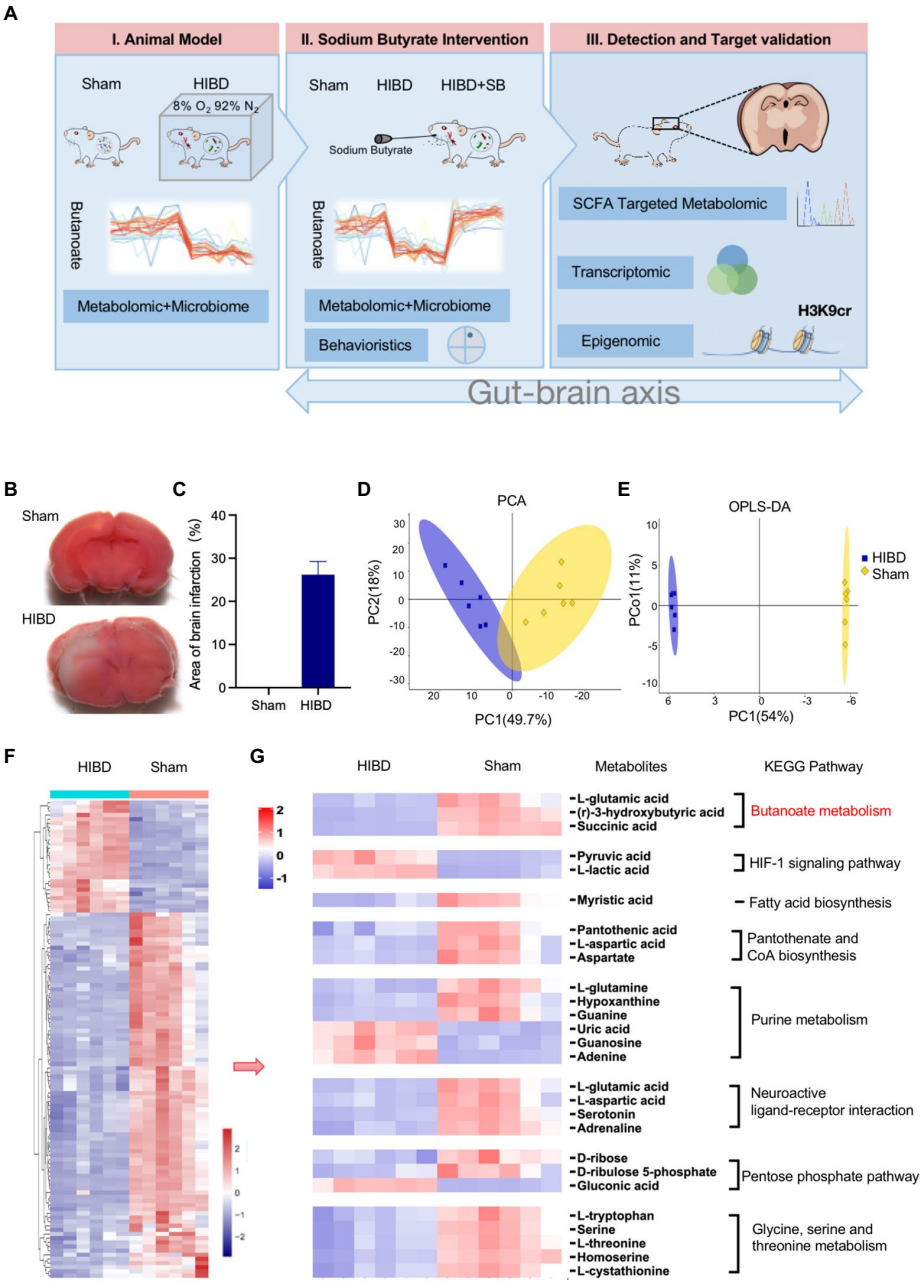
(**A**) Experimental flow chart depicting this study. Sham “sham operated group”, HIBD “hypoxic–ischemic brain damage group,” HIBD + SB “sodium butyrate (SB) treatment group.” (**B,C**) TCC staining detection and Image J quantification of the area of brain infarction, *n* = 3, error bars represent SD. (**D,E**) PCA analysis to the overall distribution and stability of each sample, and (O)PLS-DA to distinguish the global metabolic differences between groups and identify the differential metabolites between Sham and HIBD group (*n* = 6). (**F**) Heat map of all differential metabolites between the Sham and HIBD group (*n* = 6, all *p* < 0.05). (**G**) Representative differential metabolites in the Sham and HIBD group were screened out, the KEGG terms shown on the right side (*n* = 6, all *p* < 0.05).

First, we established the Hypoxic–ischemic P7-P10 neonatal rats model as previous studies by the classical Rice–Vannucci method. A flowchart depicting the analyses of this study is shown in Figure 1A. 2,3,5-Triphenyltetrazolium chloride staining was conducted to evaluate the extent of brain damage. The 2,3,5-triphenyltetrazolium chloride staining results at 24 h after HIBD induction are illustrated in Figures 1B,C. No cerebral infarction occurred in the Sham group. In the HI group, a significant infarct volume (29.5 ± 2.10%) was observed in the injured brain hemisphere. The gut fecal material from Sham and HIBD group were harvested for microbiome and metabolome characterizations through 16S rRNA gene sequencing and gas chromatography–mass spectrometry metabolomic analyses. It reveals a significant difference between the Sham group and the HIBD group through principal component analysis (PCA) in Figure 1D. The two groups of samples exhibit significant differences in the orthogonal projections to latent structures discriminant analysis (OPLS-DA) scores (Figure 1E). We also identified differentially abundant metabolites between the Sham and HIBD groups. A total of 115 metabolites with significant differences were identified (Figure 1F). Next, we examined the Kyoto Encyclopedia of Genes and Genomes (KEGG, Supplementary Table S1) pathway and pathway-related metabolites among the identified metabolites, including butyrate metabolism, the HIF-1 signaling pathway, fatty acid biosynthesis, pantothenate and CoA biosynthesis, purine metabolism, and neuroactive ligand-receptor interactions, which were screened and visualized using a heat map. For example, (R)-3-hydroxybutyric acid, l-glutamic acid and Succinic acid in butyric metabolic pathway were significantly down-regulated in HIBD group (Figure 1G; Supplementary Figure S3, *p* < 0.05). Taken together, these results suggested that the fecal metabolic phenotype was significantly changed in HIBD rats.

### Decrease of SCFAs-producing bacteria exhibited in the gut microbiota in HIBD rats

**Figure 2:**
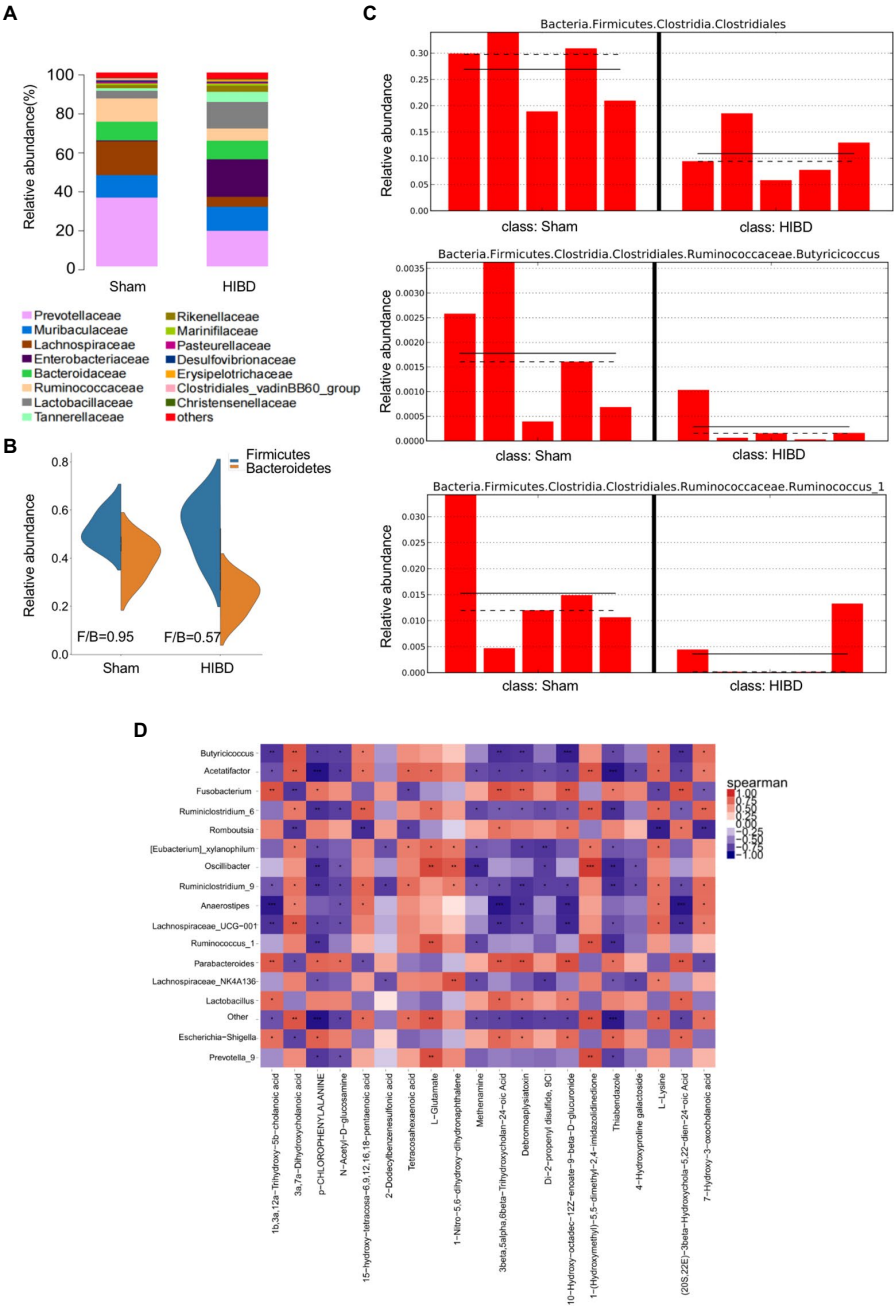
(**A**) Taxonomic summary of the gut microbiota of two groups at family level. The abscissa represents the group, and the ordinate represents the relative abundance (*n* = 5). (**B**) *Firmicutes/Bacteroidetes* ratio of the gut microbiota of two groups at phylum level. (**C**) Histogram of relative abundance of short-chain producing bacteria, where the solid line is the average value of relative abundance and the dotted line is the median value of relative abundance. Each column represents the relative abundance of each sample in each group (all LDA threshold value >2.0, *n* = 5). (**D**) Correlation analysis of metabolomic and microbiome of two groups. The color scale represents the strength of correlation, ranging from 1 (strong positive correlation) to −1 (strong negative correlation). *p < 0.05, **p < 0.01, and ***p < 0.001.

Gut microbiota is closely related to fecal metabolites. Fecal samples were collected from Sham and HIBD groups rats, and a total of 15 bacterial family were identified, with the remaining bacteria classified as “others” (Figure 2A). In addition, the *Firmicutes/Bacteroidetes* ratio is widely believed to have an important influence on maintaining normal intestinal homeostasis, and an increase or decrease in the ratio is regarded as dysbiosis (Indiani et al., 2018; Stojanov et al., 2020). We identified that the *Firmicutes/Bacteroidetes* ratio of the HIBD group was significantly decreased compared with that of the Sham group (Figure 2B). In our study, 51 differentially abundant taxons were found between the HIBD and Sham group (Supplementary Figures S1, S2). We next examined differences in the abundance of several common SCFAs-producing bacteria between the two groups using the linear discriminant analysis effect size. The most abundant butyrate-producing bacteria belong to the *Clostridiales* cluster (Eeckhaut et al., 2013; Ratajczak et al., 2019). *Ruminococcus* and *Butyricicoccus* from *Clostridiales* cluster, are tightly associated with butyrate production (Figure 2C). To determine whether the distribution of fecal metabolic and the microbiota profiles were correlated, we used a Pearson's correlation analysis. The resulting metabolic association heatmap (Figure 2D) indicates positive and negative correlations between the metabolic levels and the identified gut microbiota at the genus level. *Ruminococcus*, displayed a strong positive correlation with L − Glutamate (Correlation = 0.84, *p* = 0.004) and negative correlation with p-Chlorophenylalanine (Correlation = −0.81, *p* = 0.006). *Butyricicoccus*, displayed a positive correlation with l-Lysine (Correlation = 0.74, *p* = 0.01) and also negative correlation with p-Chlorophenylalanine (Correlation = −0.75, *p* = 0.01).

### SB ameliorated pathological brain injury and behavioral function in HIBD rats

**Figure 3:**
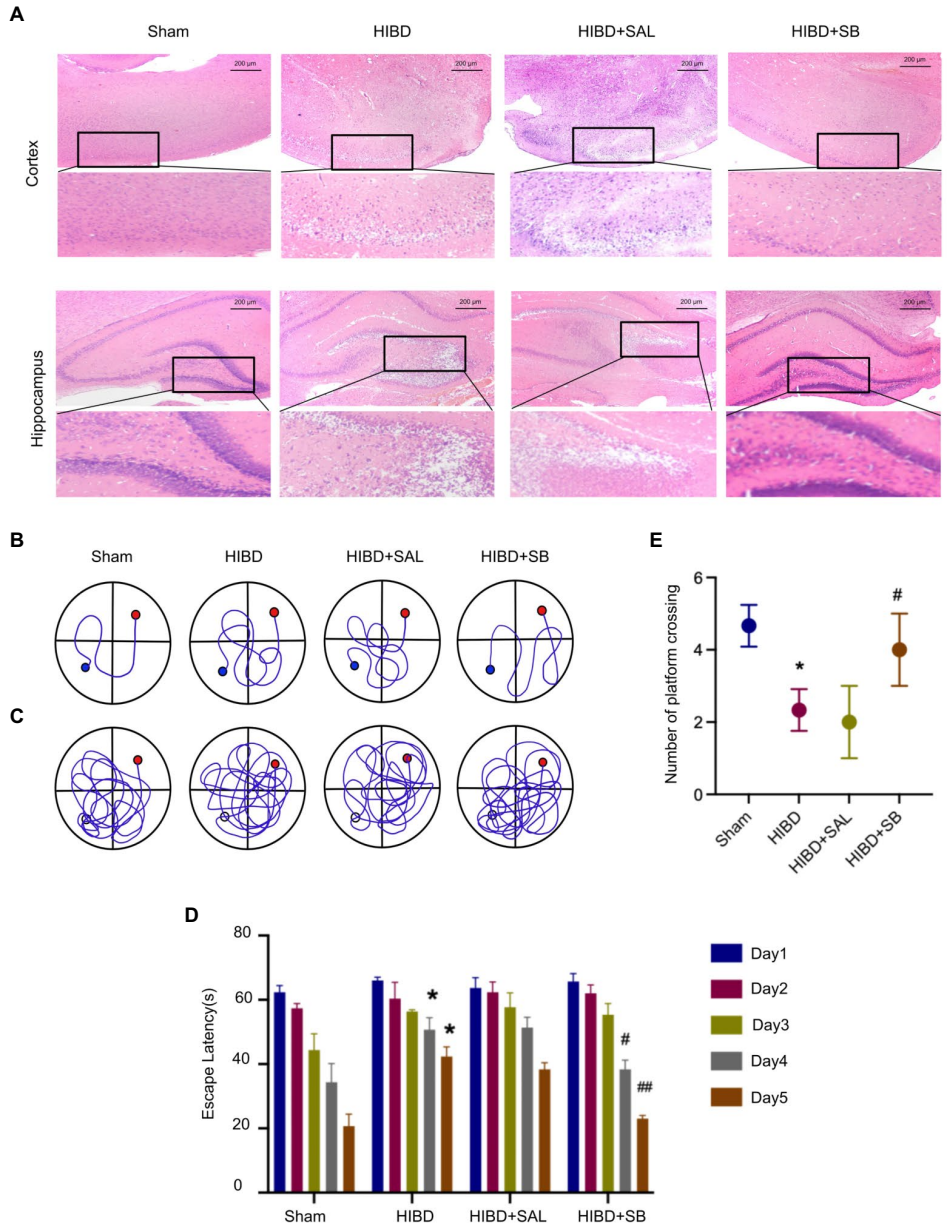
(**A**) H&E staining of cerebral cortex and hippocampus tissue pathological changes of rats. (**B–E**) SB ameliorated learning memory deficits in hypoxia-ischemia brain damage Rats: Representative pathways on the fifth day of navigation test (**B**); representative swim paths on the sixth day of test (**C**); Escape latency (**D**); Number of times the rat pups crossed the former location of the platform (**E**); *n* = 3, error bars represent SD, * < 0.05 compared to Sham group; ^#^ < 0.05, ^##^ < 0.01 compared to HIBD group.

The hematoxylin and eosin staining results revealed that the Sham group exhibited an intact neuron structure and regular arrangement with red staining of the cytoplasm and blue staining of the nuclei in both the cerebral cortex and hippocampus. However, hypoxia-ischemia induced obvious neuronal injury in the cerebral cortex, including neuronal shrinkage, nuclear chromatin condensation, and loss of intercellular connections. Heterogeneity was also obvious in the hippocampus, shown as a disordered arrangement of neurons, an unclear hierarchy, a significantly reduced number of neurons, and partial nucleoplasmic shrinkage. After 2 weeks of intragastric SB administration, these pathological changes in the cerebral cortex and hippocampus were significantly reduced, as revealed by normal-shaped nerve cells in most areas compared with the HIBD group (Figure 3A). The Morris water maze was used to investigate the effects of SB on learning and memory deficits in rats after hypoxia-ischemia. Analysis of the mean path length showed that memory was significantly improved in SB-treated rats (Figures 3B,C). Figure 3D shows that SB treatment reduced the escape latency on day 4 and day 5 (^#^*p* < 0.05, ^##^*p* < 0.01). A decrease in the number of times that rats crossed the former location of the platform was observed in the HIBD group versus the sham group (*p < 0.05), and supplementation with SB reversed this effect (^#^*p* < 0.05), as shown in Figure 3E. This experiment also confirmed that intragastric saline administration had no effect on brain histopathology and behavioral function in hypoxic–ischemic rats. These results suggest that SB can alleviate pathological brain injury and rescue learning and memory abilities in rats after hypoxia-ischemia.

### Effects of SB treatment on the gut microbiota and metabolites in HIBD rats

**Figure 4:**
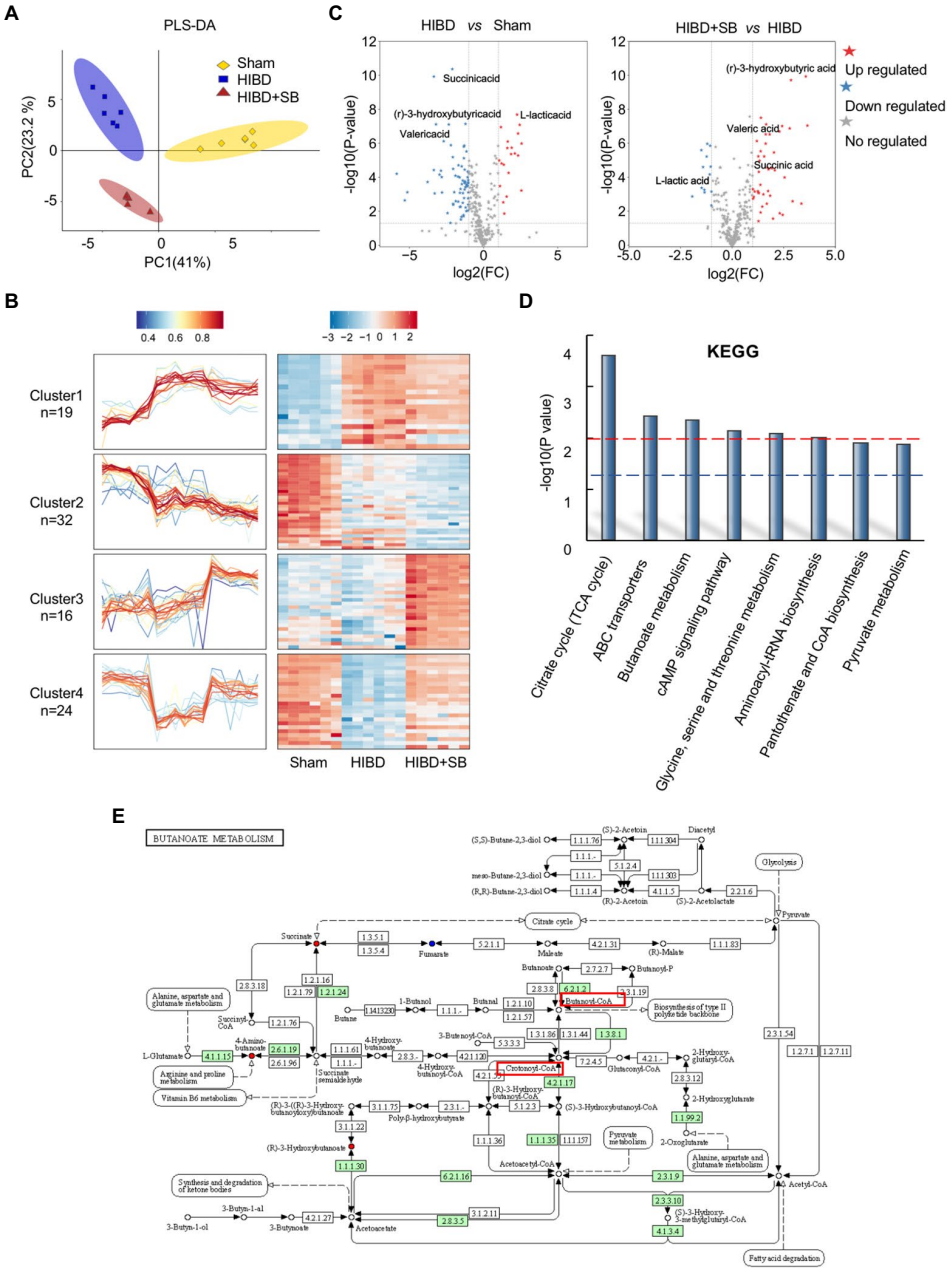
(**A**) PLS-DA to distinguish the overall metabolic differences between the Sham, HIBD and HIBD + SB group (*n* = 6 per group). (**B**) Cluster heat map of differential metabolites (*n* = 6 per group, *p* < 0.05). (**C**) Volcano plots of all differential metabolites between three groups (all *p* < 0.05). (**D**) Metabolic pathway enrichment analysis of differential metabolites was performed based on KEGG database between HIBD + SB and HIBD groups, where *p* = 0.01 indicated by the red line and *p* = 0.05 indicated by the blue line. (**E**) KEGG pathway of butanoate metabolism.

**Figure 5:**
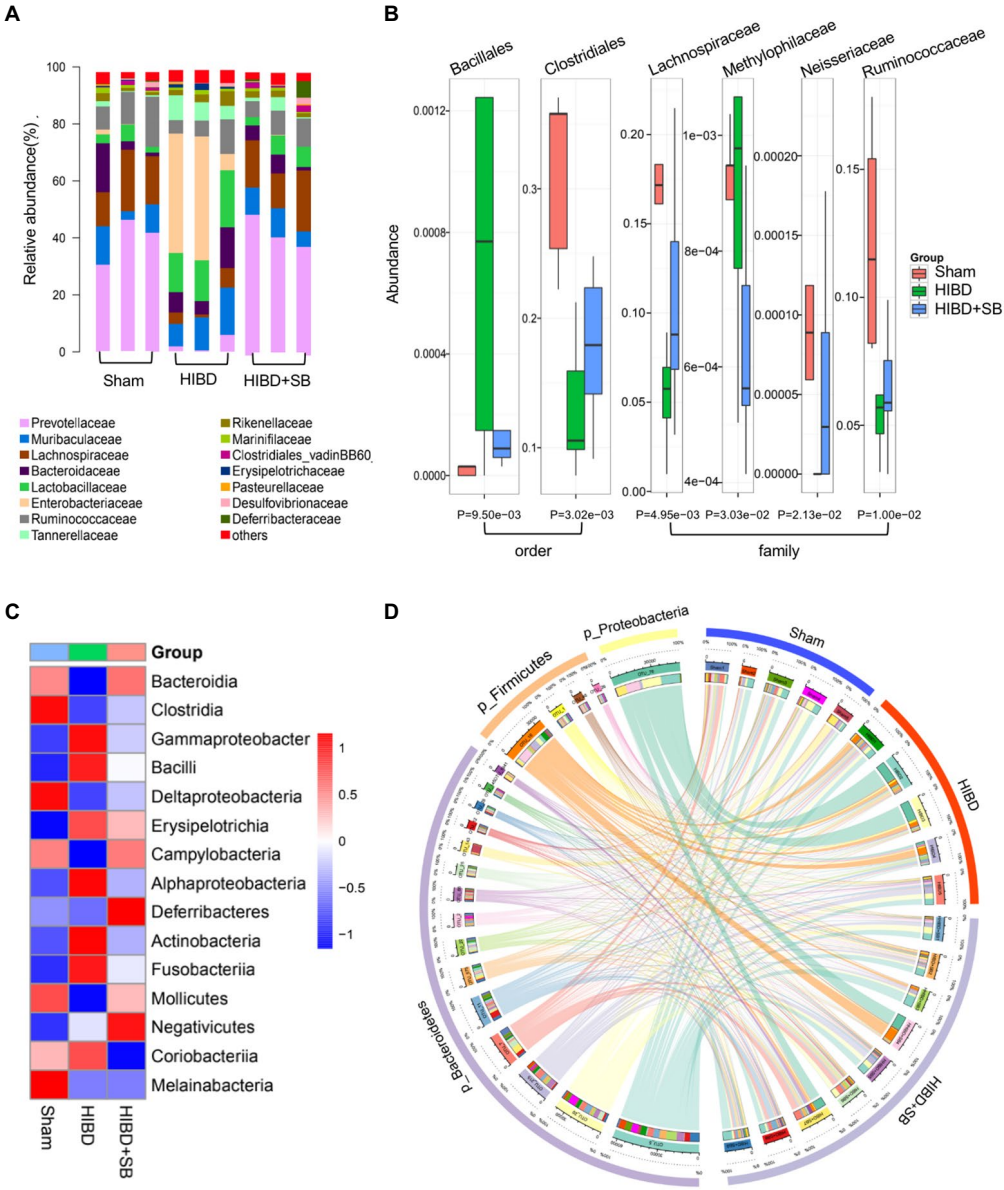
(**A**) Taxonomic summary of the gut microbiota species between the Sham, HIBD and HIBD + SB group at family level. Each column represents a sample, different colors represent different annotation information, and the ordinate represents the relative abundance (*n* = 3 per group). (**B**) The Boxplot of microbiota between groups at order and family level, different colors represent different groups of samples, and the ordinate represents the relative abundance value of species, the bottom label is *p* value (*n* = 5 in Sham and HIBD group, *n* = 8 in HIBD + SB group). (**C**) Heat map of microbiota species at class level, red indicates high relative abundance of species, blue indicates low relative abundance of species (*n* = 5 in Sham and HIBD group, *n* = 8 in HIBD + SB group). (**D**) The outer circle and inner circle of the left semicircle represent the species information at phylum level with Top5 abundance and their proportions in different groups, respectively. The outer and inner circles of the right semicircle represent the sample grouping information and the proportion of species contained at the Top5 phylum level.

Multivariate statistical analysis showed that the experimental group exhibited good stability and repeatability, which could be used to explain and predict the differences among the three groups (Figure 4A). To more intuitively demonstrate the effect of SB treatment on fecal metabolism and the differences in metabolites among the three groups, we conducted hierarchical clustering analysis of all metabolites with significant differences (*p* < 0.05, Supplementary Table S2). The Mfuzz method was used to perform cluster analysis on the expression of the differential metabolites. As shown in Figure 4B, this analysis identified 4 clusters referring to as clusters 1–4. We focused on clusters 4, those metabolites decreased in HIBD group and returned after SB treatment. As expected, the molecules in cluster 4 (*n* = 24) are associated with butyrate metabolism, which labels in the volcanic diagram (Figure 4C). Differential metabolite pathway enrichment analysis can be helpful for understanding the mechanism of brain injury recovery after SB treatment. We listed related functional pathway metabolites that were differentially abundant between the HIBD + SB and HIBD groups (Figure 4D). The butanoate metabolism pathway and related metabolites are shown in Figure 4E. In addition, SB altered the structure and composition of the gut microbiota. Figure 5A shows a histogram of the community structure of the top 15 species in order of abundance at the family classification level (Supplementary Table S3). In particular, *Prevotellaceae* were significantly reduced in the HIBD group compared with the Sham group, but this effect was reversed in the HIBD + SB group. *Enterobacteriaceae* and *Lactobacillaceae* were significantly more abundant in the HIBD group than in the other groups. A boxplot analysis of relative abundance was performed at the family and order levels for intergroup comparisons of dominant SCFAs-producing species (Figure 5B), and a heatmap was constructed according to the relative abundances of different species (Figure 5C). A Circos diagram was used to show the corresponding relationship between the groups and microbiota, which showed the composition proportion of the top 5 dominant species at the phylum level in each sample and their proportional distribution among the groups (Figure 5D). The linear discriminant analysis effect size indicated differences in the abundance of several common SCFAs-producing bacteria (butyrate in particular) among the three groups (Supplementary Figure S4). The differentially abundant metabolites and gut metabolic association heatmap of the microbiota (Supplementary Figure S5) indicated positive and negative correlations between the HIBD and HIBD + SB groups. These results indicate that SB treatment effect on SCFAs-producing bacteria exerts its SCFAs-producing species activity by activation of butanoate metabolism pathway. This led us to further investigate the mechanism of SB treatment-mediated refractoriness.

### SB treatment promoted H3K9cr in the brain tissue of hypoxic–ischemic rats

**Figure 6:**
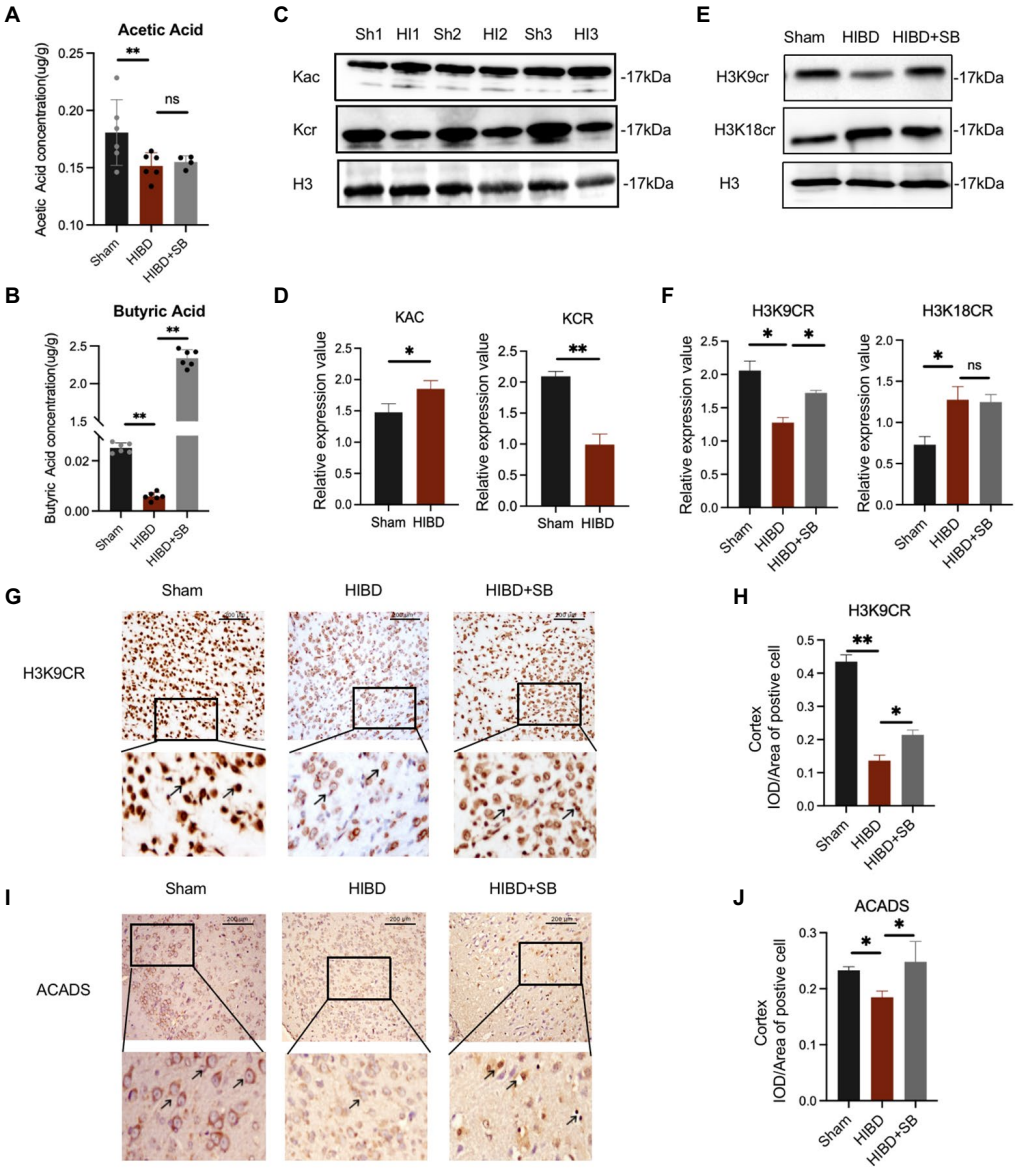
(**A,B**) The concentration of acetic acid and butyric acid in the brain tissue (μg/g). Data are shown as mean ± SD, *n* = 6, error bars represent SD, Statistical significance was considered at ns: not significant, *p < 0.05, **p < 0.01 for one-way ANOVA analysis. (**C**) Changes of acetylation and crotonylation in brain tissue after HI which was analyzed by Western blot with anti-Kac and anti-Kcr antibodies. (**D**) Relative abundance analysis of (**C**), *n* = 3, error bars represent SD, *p < 0.05, **p < 0.01 for one-way ANOVA analysis. (**E**) Brain H3K9cr and H3K18cr expression was analyzed by Western blot of Sham, HIBD and HIBD + SB group. (**F**) Relative abundance analysis of (**E**), *n* = 3, error bars represent SD, ns: not significant, *p < 0.05, **p < 0.01 for one-way ANOVA analysis, error bars are standard deviation. (**G**) Immunohistochemical of the H3K9cr expression level of cerebral cortex in three groups. (**H**) Relative abundance analysis of (**G**), *n* = 3, error bars represent SD, *p < 0.05, **p < 0.01 for one-way ANOVA analysis, error bars are standard deviation. (**I**) Immunohistochemical of the ACADS expression level of the cerebral cortex in three groups. (**J**) Relative abundance analysis of (**I**), *n* = 3, error bars represent SD, ns: not significant, *p < 0.05, for one-way ANOVA analysis, error bars are standard deviation.

Several research showed that the gut microbiome plays an important role in basic neurogenerative processes such as the formation of the blood–brain barrier, neurogenesis, and microglia maturation, a subsequence effect on animal behavior. To explore the mechanism of butyrate intervention in alleviating cerebral hypoxic–ischemic injury, we measured the SCFAs concentration in brain tissue using targeted high-performance liquid chromatography–tandem mass spectrometry. SCFAs, including acetic acid (*p* = 0.040) and butyric acid (*p* < 0.001), were decreased in the HIBD group compared with the Sham group. In particular, the butyric acid concentration in the brain increased significantly after butyrate supplementation in the HIBD + SB group compared with HIBD rats (*p* < 0.001; Figures 6A,B). This result is consistent with previous reports indicating that butyrate penetrates the BBB. The correlation analysis pointed out that *Butyricicoccus* and *Ruminococcus* which decrease in feces of HIBD rats displayed a positive correlation with acetic acid and butyric acid in the brain (Supplementary Figure S6). Furthermore, studies have suggested that SCFAs are converted into cognate acyl-CoAs, which are directly used as substrate factors for histone acylation reactions. Western blot analysis results indicated that histone crotonylation was relatively abundant in the brain and more significantly altered in the HIBD group than in the Sham group (acetylation *p* = 0.041, crotonylation *p* < 0.01) (Figures 6C,D). We hypothesized that histone crotonylation may be linked to hypoxic–ischemic brain injury. We tested this hypothesis by examining the H3K9cr and H3K18cr expression levels in brain tissues from the three groups. The results indicated that H3K9cr was decreased in the HIBD group, and the protein expression level increased after SB treatment. H3k18cr was also altered in the HIBD group, but SB treatment did not affect its expression level (Figures 6E,F). Immunohistochemical analyses showed a similar trend in H3K9cr expression levels in the cerebral cortex (Figures 6G,H). We also found that acyl-CoA dehydrogenase short chain (ACADS) expression was greater in the Sham group than that in the HIBD group, and the number of ACADS-positive cell nuclei in the cerebral cortex greatly increased after SB treatment (Figures 6I,J; Supplementary Figures S7A,B).

### Alterations in the gene expression profile after SB treatment

**Figure 7:**
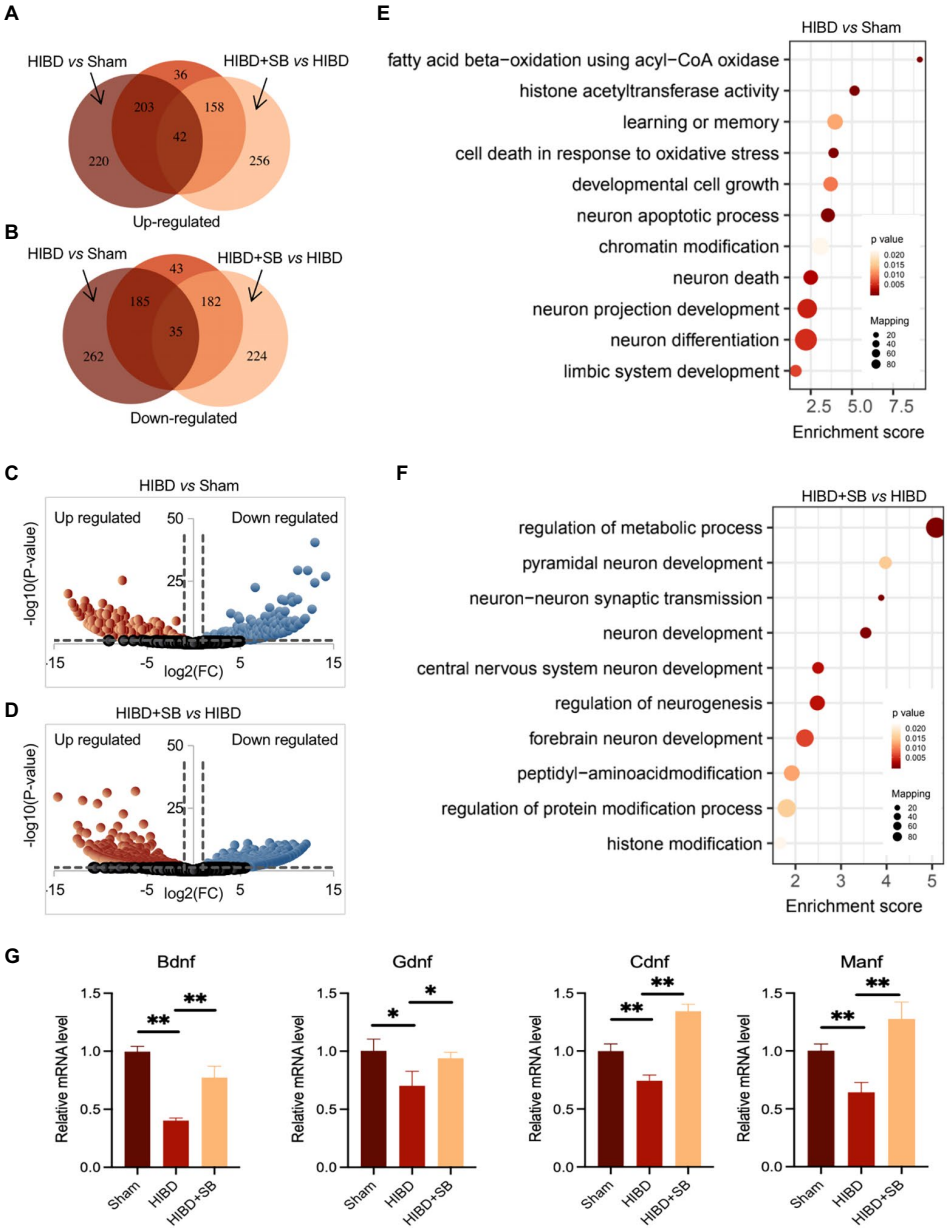
The total RNA was analyzed using RNA-seq. Identified-differential expression genes in Sham, HIBD and HIBD + SB group, which were shown by Venn (**A,B**) and volcano diagram (**C,D**). (**E,F**) The GO terms and corresponding enrichment *p* values in three groups. (**G**) HIE-related neurotrophic factor genes *Bdnf*, *Gdnf*, *Cdnf* and *Manf* mRNA extracted from the brain in three groups were assessed by RT-qPCR. GAPDH was used as control. *p < 0.05, **p < 0.01, *n* = 3, error bars represent SD.

To identify alterations in the gene expression profile caused by hypoxia-ischemia and SB treatment, we performed RNA sequencing on cerebral cortex tissue. Differentially expressed genes were identified in the Sham, HIBD, and HIBD + SB groups, which are shown by Venn (Figures 7A,B; Supplementary Tables S4,S5) and volcano diagrams (Figures 7C,D). By comparing libraries from the HIBD and Sham groups, we identified a number of significantly differentially expressed genes, including 465 upregulated and 482 downregulated genes. Compared with the HIBD group, there were 456 upregulated and 441 downregulated genes after SB treatment. Further functional analysis with Gene Ontology (GO) terms indicated that enriched differentially expressed genes were related to neuron development, metabolic processes, and chromatin/histone modification (Figures 7E,F). Quantitative reverse transcription polymerase chain reaction analysis revealed that several HIE-related neurotrophic factor genes were downregulated in the HIBD group and upregulated in the HIBD + SB group, including *Bdnf*, *Manf*, *Ogdh,* and *Cdnf* (Figure 7G). Taken together, our data suggest that hypoxia-ischemia resulted in changes in the brain gene expression pattern and decreased expression of HIE-related neurotrophic factor genes, and this phenomenon can be reversed by SB treatment.

### H3K9cr binding to HIE-related neurotrophic factor genes in hypoxic–ischemic rat brains and changes in these genes after SB treatment

**Figure 8:**
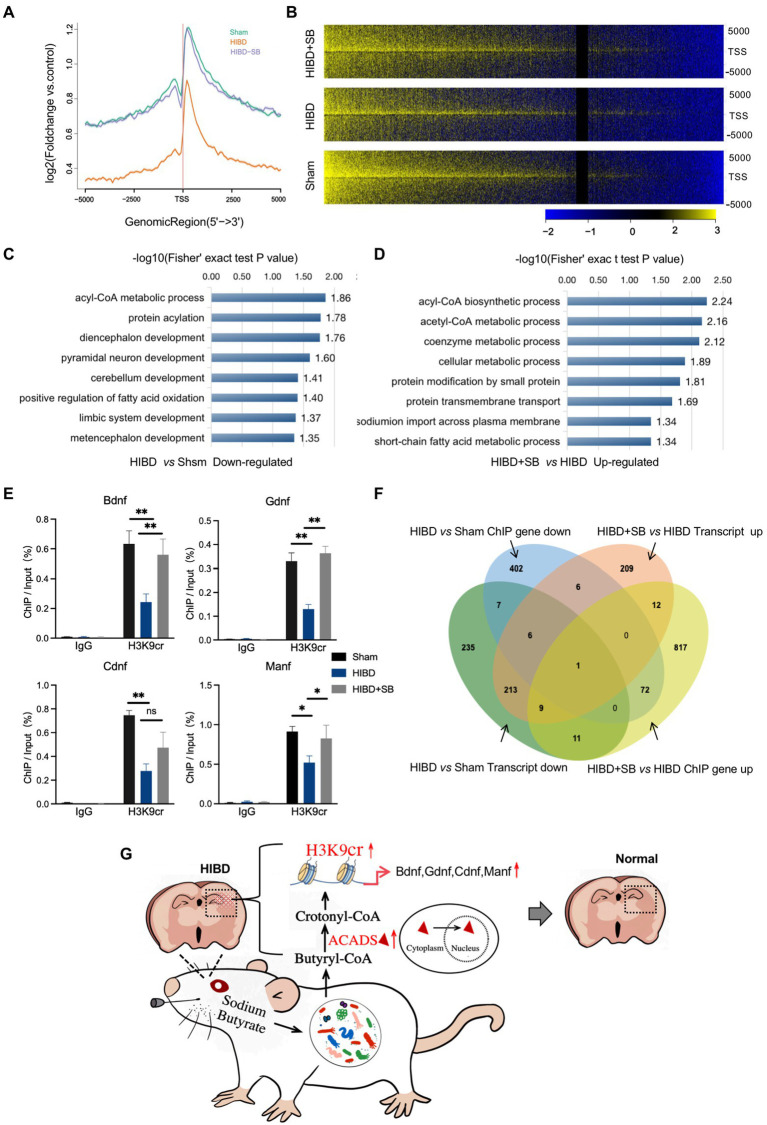
(**A**) Comparison of average ChIP-Seq peaks aggregated around TSS for H3K9cr between Sham, HIBD and HIBD + SB group rats. (**B**) ChIP-Seq enrichment profiles for H3K9cr levels in Sham, HIBD and HIBD + SB group rats. (**C**) Genes corresponding to differential enrichment peaks in the promoter region (TSS − 2000 to TSS + 2000) were used to GO analyze. The bar chart shows the significantly down-regulated GO terms and their adjusted *p*-values in the HIBD compared with Sham group. (**D**) The bar chart shows the significantly up-regulated GO terms and their adjusted *p*-values in the HIBD + SB compared with HIBD. (**E**) Enrichment of HIE-related neurotrophic factor genes *Bdnf*, *Gdnf*, *Cdnf* and *Manf* was measured by CHIP-qPCR. IgG was used as control, *n* = 3, *p < 0.05, **p < 0.01, error bars represent SD. (**F**) Venn diagram of ChIP seq and RNA seq combined analysis. (**G**) After SB intragastric treatment, the gut microbiota composition and the fecal metabolic phenotype in neonatal HIBD rats were significantly changed. SB is absorbed by gut microbiota and metabolized primarily as butanoyl-CoA, and further converted to crotonyl-CoA by ACADS in the nucleus. These molecular metabolites ultimately alleviate brain injury by specifically regulating the H3K9cr enrichment level and binding genes *Bdnf*, *Gdnf*, *Cdnf*, and *Manf.*

Our data suggest that a decrease in H3K9cr in the brain may underlie brain injury during hypoxia-ischemia. To further explore the importance of H3K9cr during neural system injury and repair, chromatin immunoprecipitation sequencing (ChIP-seq) was carried out in cerebral cortex tissues from the three groups. By analyzing equal numbers of reads for H3K9cr, 6007, 3752, and 4220 total peaks in the promoter were detected in the Sham, HIBD, and HIBD + SB groups, respectively, using an anti-H3K9cr antibody and scanning through the entire rat genome. ChIP-seq of H3K9cr target genes showed that the enrichment level of H3K9cr was significantly decreased in the HIBD group compared with the Sham group, and the enrichment level was successfully rescued by SB treatment (Figure 8A). ChIP-seq analysis identified a remarkable overall change in H3K9cr near transcription start site genomic regions in the three groups (Figure 8B, Supplementary Tables S6,S7). An analysis of ChIP GO terms in the HIBD and Sham groups showed that the significantly downregulated genes targeted by H3K9cr were related to nervous system development, especially the limbic system, diencephalon, metencephalon, and cerebellum, and CoA metabolic processes (Figure 8C). A bar chart shows the significantly upregulated GO terms in the HIBD + SB group compared with the HIBD group (Figure 8D). We analyzed the specific region and accumulation of H3K9cr peaks for the *Bdnf*, *Manf*, *Ogdh*, and *Cdnf* genes (Supplementary Figure S7C). ChIP-qPCR results showed that H3K9cr binding of the HIE-related neurotrophic factor genes *Bdnf*, *Manf, Ogdh,* and *Cdnf* was significantly downregulated in HIBD rat brains and was significantly upregulated by SB treatment (Figure 8E). The venn diagram of ChIP seq and RNA seq combined analysis shows there were 14 genes were down-regulated synchronously in ChIP and transcript in HIBD group and 22 genes were up-regulated synchronously after SB treatment (Figure 8F). These data indicated that decreased binding of H3K9cr to HIE-related neurotrophic factor genes in cerebral cortex tissue of hypoxic–Ischemic rats, resulting in repression of these genes. Furthermore, SB treatment enhances H3K9cr expression and affects H3K9cr binding to HIE-related neurotrophic factor genes.

## Discussion

The gut microbiome is currently recognized as a key factor that regulates host health and has a profound influence on host physiology by regulating the metabolome, transcriptome, and epigenome. In the present study, we report a bidirectional association between the gut microbiome and brain through the gut–brain axis in an HIBD rat model and confirm that SB treatment reduces hypoxia-ischemia-induced brain damage in the cerebral cortex and hippocampus. Although many previous studies have reported the benefits of SB in brain damage-inducing diseases, the mechanisms remain unclear. This study is the first demonstration that SB treatment alleviates hypoxic–ischemic brain injury by specifically regulating the H3K9cr enrichment level and binding genes, which extends the importance of the microbiota–gut–brain axis.

Perinatal hypoxia-ischemia may cause gut barrier dysfunction, such as necrotizing enterocolitis (Young et al., 2011). HIE has also been reported to alter the gut microbiota (Ni et al., 2019; Gala et al., 2022). We found a decreased Firmicutes/Bacteroidetes ratio in HIDB rats, which indicated a dysbiosis in the gut microbiota (Figure 2B). Importantly, because most of the bacteria identified by 16S rRNA were butyrate-producing phylogenetic groups belonging to the *Firmicutes* phylum and *Clostridia* class, a decreased *Firmicutes/Bacteroidetes* ratio also indicated decreased butyrate production (Turnbaugh and Gordon, 2009; Stilling et al., 2016). Next, we found that several common butyrate-producing bacteria were significantly reduced in the HIBD group (Figure 2C). When these bacteria are maintained at a certain population level, their metabolism increases the butyrate concentration and promotes other favorable gut conditions that inhibit detrimental gut bacteria, thus maintaining the gut environment and supporting optimal host health (Watkins et al., 2017; Zhao et al., 2018; Morais et al., 2021). In addition, metabolic links are present between different types of bacteria. For example, acetic acid produced by Bacteroidetes can be used by Firmicutes to produce butyric acid. *Lachnospiraceae* have the ability to produce butyric acid in the presence of lactic acid and acetic acid (Martin-Gallausiaux et al., 2018; Sun et al., 2018). In our study, butanoate metabolism, fatty acid biosynthesis, and other KEGG pathways exhibited significantly low enrichment in fecal metabolomic analysis in the HIBD group (Figure 1F). Some studies have noted that oral or intravenous sodium butyrate supplementation regulates brain metabolic activity, neurogenesis, and hippocampal structural plasticity (Val-Laillet et al., 2018; Sun et al., 2020). Based on the evidence above, we choose the butyrate as the gut–brain messenger of interest.

Butyrate produced by the gut microbiota is rapidly absorbed by intestinal epithelial cells *via* the Na^+^-coupled transporter SLC5A8 and the H^+^-coupled transporter SLC16A1, which are widely present in the central nervous system and peripheral tissues (Dalile et al., 2019). SB, as the anionic part of dissociated butyric acid and its salts (Stilling et al., 2016), plays an important role in the nervous system through the gut–brain axis. In particular, butyric acid intake shortly after birth is beneficial for gastrointestinal development in mammals (Guilloteau et al., 2010). We found significantly higher levels of butyric acid in the brain tissue of HIBD rats after 2 weeks of SB administration, suggesting that SB can cross the BBB and affect brain metabolism (Figures 6A,B). We found a higher concentration of butyric acid than that found in other previous studies of rat brains after SB supplementation, which may be because of the enhanced sensitivity for SCFAs by targeted ultra-performance liquid chromatography-electrospray-tandem mass spectrometry and the high intragastric SB concentration. Future investigations should address the intragastric concentration gradient. One study also found significant metabolic changes in several brain structures, including the hippocampus, in pigs fed a standard diet with oral sodium butyrate supplementation for 3–4 weeks (Val-Laillet et al., 2018). In addition to the effects on brain metabolism, SB showed neurogenic effects in the subgranular zone of the hippocampus and stimulated neurogenesis in the ipsilateral subventricular region during treatment of ischemia-induced brain injury (Kim et al., 2009; Tian et al., 2019). Our study reported that pathological brain injury in the cerebral cortex and hippocampus and behavioral dysfunction were obviously alleviated by SB treatment in an HIBD model (Figure 3). Meanwhile, the H3K9cr level was downregulated in HIBD rats and was upregulated in the cerebral cortex by SB treatment (Figures 6C,E). We did not confirm the target cells and molecular pathways of SB treatment after hypoxia-ischemia-induced brain injury. However, recent studies have shown the role of SB in alleviating microglia-dependent inflammation after ischemic brain injury. Future investigations should explore the epigenetic regulation of SB treatment in glial cells using cytological experiments.

Regarding the relationship between butyrate and histone crotonylation, it is worth noting that the gut microbiota is a potential source of dynamic acyl-CoA metabolism. SCFAs from the gut microbiota, especially butyrate, are precursors of lysine acylation, suggesting that the physiological function of SCFAs may be regulation of histone acylation modification (Sabari et al., 2017). Supplementation with SCFAs such as β-hydroxybutyric acid or crotonic acid stabilized the level of corresponding histone acylation in a dose-dependent manner through a significantly increased cell concentration of acyl-CoA (Sabari et al., 2017). Crotonylation is differentially regulated by two factors: modification enzymes and small-molecule acyl-coenzymes (crotonyl-CoA) (Fang et al., 2021). These include p300/CBP as crotonyltransferases and class I histone deacetylases, HDAC1, HDAC2, and HDAC3 as decrotonylase enzymes (Wang et al., 2021). The HDAC2 level in the brain was slightly down-regulated in the following of SB treatment (Supplementary Figures S7D,E), which was not correlated with significantly up-regulated butyric acid level in the brain (Figure 6B). Therefore, we hypothesized that the regulation on HDAC2 has only a partial effect on butyrate for crotonylation regulation. However, the intracellular concentration of crotonyl-CoA is approximately 600- to 1,000-fold lower than that of acetyl-CoA. Thus, crotonyl-CoA may be a limiting factor in the transfer reaction of crotonylation (Fang et al., 2021). Interestingly, butyrate is a source of crotonyl-CoA. We speculated that butyrate may regulate histone crotonylation by acting as a decrotonylase and providing crotonyl-CoA.

As expected, we found that the staining of total ACADS was also decreased in cerebral cortex tissue of HIBD samples compared with Sham (Figures 6I,J). One study performed nitrate transfer experiments to further demonstrate that extracellular butyric acid is absorbed by *Bacteroides* and metabolized primarily as butanoyl-CoA (Li, 2022; Park et al., 2022). The conversion of butyric acid to butyryl-CoA was mediated by acyl-CoA synthase short chain family member 2 (ACSS2). ACADS is believed to be a mitochondrial protein that participates in the β-oxidation spiral, where it reduces butyryl-CoA to crotonyl-CoA (Yang et al., 2021). Interestingly, we observed that the number of ACADS-positive cell nuclei greatly increased after SB treatment. Eu et al. showed that ACADS is both a nuclear protein and a mitochondrial protein that is located in the cytoplasm and nucleus and proposed that its function is to participate in the β-oxidation pathway in the nucleus and regulate butanoyl-CoA accumulation (Yu et al., 2020; Yang et al., 2021). Li also showed that crotonyl-CoA-producing enzymes, such as ACADS, were localized to the nucleus, especially in endoderm cells, suggesting that these enzymes play a role in the nucleus (Fang et al., 2021). This evidence explains the increase in nuclear ACADS expression after SB treatment observed in our study. Another key piece of evidence is that ACADS knockout significantly decreased the H3K9Cr level, suggesting H3K9Cr is regulated by ACADS (Yang et al., 2021).

As mentioned above, the effect of the gut–brain axis is bidirectional. Therefore, the effect of SB treatment on the gut microbiome and metabolic profiling while alleviating brain injury remains to be determined. In this study, we similarly observed that SB treatment changed the gut microbiota composition and obviously altered the fecal metabolic phenotype, including an increase in SCFAs-producing bacteria and substantial enrichment of the citric acid cycle and the pantothenate and CoA biosynthesis metabolism pathway. Metabolism and gene expression regulate each other, and epigenetic modification usually acts as a molecular link between the two fundamental processes (Ren et al., 2021). Histone modifications, such as acetylation and crotonylation, are closely related to transcriptional regulation, and crotonylation is a more potent transcriptional activator than acetylation (Liu et al., 2017; Ratajczak et al., 2019; Wang et al., 2021). Of note, some studies have shown that crotonylation is relatively enriched near the transcription start sites of genes involved in various metabolic processes in pluripotent stem cells (Fang et al., 2021). Our ChIP-seq results for H3K9 crotonylation demonstrated that the enrichment level was significantly decreased and the expression levels of HIE-related neurotrophic factor genes were downregulated, while the enrichment level and binding of genes were restored by SB treatment in the HIBD rat brain (Figures 7G,8E). The role in promoting neuron growth and neuroprotection of brain derived neurotrophic factor (BDNF) has been confirmed in neonatal HIE and animal HIBD models. In agreement, study have confirmed that the decrease of serum BDNF level after HIE may be related to long-term hypoxia in uteros (Chavez-Valdez et al., 2021; Xue et al., 2021). Glial cell-derived neurotrophic factor (GDNF) is one of the most effective neurotrophic factors isolated from glial cells After HIBD. During cerebral ischemia, GDNF can reduce infarct size by preventing neuronal apoptosis (Li et al., 2014). The neurotrophic role of mesencephalic astrocyte derived neurotrophic factor (MANF) and cerebral dopamine neurotrophic factor (CDNF) has been demonstrated in several models of neurological diseases and crosstalk with BDNF and GDNF (Konovalova et al., 2021). Similar observations have shown that SB regulates the H3K9ac level on the gene promoter and enhances the neuro-protective function of microglia during ischemic stroke (Patnala et al., 2017). Previous studies showed that butyrate influences both histone acetylation and crotonylation as an HDAC inhibitor. In this study, the P7-P10 rats were selected for the HIBD model. Thus, we speculate that different chromatin modifications are introduced and removed with very different time windows, in which some chromatin modifications may react more dynamically to changes in time and development status. The future studies are required for confirming whether and how the histone crotonylation and acetylation dynamically regulate in the distinct time windows.

In summary, our study highlights the importance of microorganisms and their metabolites for neonatal brain development. This study revealed that SB induced H3K9cr and alleviated hypoxic–ischemic brain injury through the gut–brain axis. Butyrate from the gut microbiota is a potential source of crotonyl-CoA, a necessary molecule for histone crotonylation. Furthermore, SB supplementation rescued the decrease in the H3K9cr enrichment level observed in HIBD rats and ameliorated pathological brain injury and behavioral function. We demonstrated that butyrate connects neurogenesis and the gut microbiota *via* histone crotonylation. This study provides important evidence for fecal microbiota transplantation and a novel ecological approach for HIE treatment and prevention.

## Data availability statement

The data presented in the study are deposited in the NCBI BioProject and GEO repository, accession numbers PRJNA838931 and GSE205144. The mass spectrometry data for metabolomics is accessible at http://www.ebi.ac.uk/metabolights/, accession numbers MTBLS4932 and MTBLS4933.

## Ethics statement

The animal study was reviewed and approved by Ethics Committee of Central Hospital Affiliated to Shandong First Medical University.

## Author contributions

SW, MB, and HZ conceived and designed this study. XH, YZ, and PP participated in laboratory work. WJ and YL performed the data analysis. SW and XH participated in the writing of the manuscript. SW and TZ participated in advising and revising the manuscript critically. All authors contributed to the article and approved the submitted version.

## Funding

This work was supported by the National Natural Science Foundation Projects (82071690 and 81971390), Research Foundation of Capital Institute of Pediatrics (FX-2020-05 and CXYJ-2-21-09), Public service development and reform pilot project of Beijing Medical Research Institute (BMR2021-3). Beijing municipal administration of hospitals incubating program (PX2020027), Beijing Hospitals Authority Clinical Medicine Development of Special Funding (SML202110).

## Conflict of interest

The authors declare that the research was conducted in the absence of any commercial or financial relationships that could be construed as a potential conflict of interest.

## Publisher’s note

All claims expressed in this article are solely those of the authors and do not necessarily represent those of their affiliated organizations, or those of the publisher, the editors and the reviewers. Any product that may be evaluated in this article, or claim that may be made by its manufacturer, is not guaranteed or endorsed by the publisher.
