# Supplementary material for: Sodium butyrate mediates histone crotonylation and alleviated neonatal rats hypoxic–ischemic brain injury through gut–brain axis
Source: Front Microbiol. 2022 Oct 20;13:993146. doi: 10.3389/fmicb.2022.993146 (PMC9631217; doi:10.3389/fmicb.2022.993146)
Supplement: Supplementary file 1 [file Data_Sheet_1.ZIP › Supplementary Table/Table S2.pdf]

Table S2 Significant differences metabolites

Metabolite name	Sham -1	Sham -2	Sham -3	Sham -4	Sham -5	Sham -6	HIBD-1	HIBD-2	HIBD-3	HIBD-4	HIBD-5	HIBD-6	HIBD+SB -1	HIBD+SB-2	HIBD+SB-3	HIBD+SB -4	HIBD+SB -5	HIBD+SB-6
Kynurenic acid	8.310711376	8.005936304	8.689813205	8.174292245	7.202457875	6.415599851	0.000110403	0.000705429	5.948244096	9.17311E-05	0.000225391	0.000247205	8.823093	1.9387199	6.7480112	1.756685	6.4468866	6.8390757
Scyllo-inositol	0.001437058	0.017126429	0.014901917	0.012491654	0.00828816	0.00784137	0.005830193	0.00600917	0.008012748	0.012082295	0.017680391	0.018821858	0.18205	0.1601748	0.1336537	0.13003	0.1307127	0.1221764
Caproic acid	0.029742096	0.023861169	0.022911011	0.025035922	0.015033392	0.008719941	0.000317933	0.000383143	0.000413474	0.000384069	0.000345129	0.000310075	0.00428	0.0043284	0.0044552	0.00475	0.0040251	0.0037266
D-fructose -1-phosphate	0.003411891	0.002323247	0.002135762	0.002067323	0.001017534	0.000718998	6.10047E-05	2.08748E-05	9.60189E-05	5.46039E-05	5.19069E-05	4.56507E-05	4.24E-05	5.606E-05	4.292E-05	3.07E-05	2.684E-05	3.985E-05
3,6-anhydro- d-galactose	0.127451923	0.085187572	0.075082104	0.069527981	0.029783243	0.017757477	0.001525409	0.001531008	0.00172926	0.001738795	0.001717329	0.00190159	0.005449	0.0038381	0.002927	0.003396	0.0031743	0.0028852
Glycyl tyrosine	0.191000546	0.30314089	0.324489651	0.362646654	0.385211967	0.767429189	0.027243345	0.039077233	0.071701281	0.160863469	0.259136052	0.26952234	1.098773	1.0772613	0.857311	0.9069	0.8562519	0.9218421
(r)-3-hydroxybutyric acid	0.002913297	0.002921097	0.003485724	0.003171681	0.002690987	0.002194741	0.000163475	0.000171735	0.000309076	0.000302339	0.000448204	0.000479584	0.002392	0.0021446	0.0023942	0.002324	0.0020954	0.0021892
Pinitol	0.005844683	0.008556103	0.025408394	0.023608649	0.02381599	0.009266516	0.005220394	0.008896485	0.008984074	0.02968824	0.03986866	0.037723927	0.114953	0.1102724	0.118779	0.113805	0.1120151	0.129266
Epicatechin	0.000533452	0.000470036	0.000635741	0.0005489	0.0002832	0.000594257	0.001074511	0.001345754	0.001663166	0.001640248	0.002114085	0.002032492	0.009998	0.0068751	0.0040761	0.005328	0.0054335	0.0036682
Gamma-aminobutyric acid	0.011888813	0.018835071	0.019182588	0.01721744	0.020016254	0.05514034	0.003276578	0.004374716	0.014250517	0.064486826	0.104901902	0.128289022	0.226549	0.2038009	0.1417097	0.169425	0.1701803	0.1572499
Pentitol	0.000622805	0.000308136	0.000432306	7.01175E-05	2.05688E-05	5.81456E-06	4.50189E-05	0.000196097	0.000140633	4.15965E-05	0.000141259	0.000481445	0.00187	0.0010073	0.0007256	0.00048	0.0007123	0.0003855
1-pentanol	0.003882213	0.003730405	0.003879305	0.004156555	0.003305556	0.008764927	0.001155452	0.001148163	0.001628832	0.001012237	0.001269917	0.001324567	0.014377	0.0129626	0.0034786	0.013271	0.0028433	0.0106706
L-arabitol	0.001086785	0.001149414	0.001155822	0.001283277	0.001081895	0.001551302	0.001075087	0.001319491	0.001434498	0.00169162	0.001739827	0.002030707	0.008254	0.007054	0.0057829	0.006457	0.0055184	0.0048083
4-hydroxymandelic acid	0.010923718	0.009705793	0.009479098	0.009570847	0.006525343	0.003856388	0.00071444	0.000768211	0.000883883	0.000930499	0.000770424	0.000810621	0.000926	0.0008766	0.0007342	0.000827	0.0001698	0.0007804
Cadaverine	0.04904996	0.059054069	0.078926161	0.068670474	0.113579439	0.276638484	0.017206736	0.021290523	0.02452116	0.026939713	0.045215196	0.041890335	0.272207	0.1943084	0.1385074	0.155821	0.1540105	0.1171338
Succinic acid	1.001302382	1.100802304	1.276055261	1.159273275	1.127619433	1.190319087	0.077474891	0.10171461	0.12195119	0.113258235	0.124226839	0.130225606	0.685174	0.539808	0.358569	0.406542	0.4298475	0.3847931
5-methyluridine	0.024928312	0.022963671	0.028603177	0.028174854	0.021221027	0.017106477	0.003694349	0.004843258	0.006487692	0.005228186	0.005191267	0.005542295	0.030642	0.0235549	0.0273382	0.027453	0.0200042	0.0229166
Tyramine	0.014762275	0.027723635	0.030048789	0.026581225	0.033236655	0.069343076	0.002533129	0.003992267	0.008897374	0.026222702	0.046445272	0.050983757	0.092122	0.0935322	0.0820421	0.084787	0.0853887	0.0885609
L-aspartic acid	0.287624009	0.258496613	0.316592399	0.270324489	0.14620381	0.072557833	0.064219662	0.078666078	0.103970711	0.054467728	0.06269118	0.0493468	0.037201	0.0252435	0.0248205	0.022393	0.0189698	0.0198161
Putrescine	0.040734347	0.036591515	0.036536109	0.033448539	0.017253319	0.012475446	0.001555073	0.001847647	0.001801352	0.002198885	0.002767368	0.002714567	0.006783	0.0053912	0.0050745	0.005861	0.0049959	0.0043047
L-glutamine	0.029757487	0.035561035	0.044318503	0.036186564	0.030641926	0.01987555	0.007402671	0.009353429	0.012256107	0.005792481	0.006789927	0.005720227	0.003565	0.0041511	0.0054538	0.00412	0.0031435	0.0031454
Erythritol	0.001691423	0.001591011	0.001714508	0.001512128	0.001258284	0.001453573	0.001610128	0.001955835	0.002311112	0.001864158	0.001933001	0.001892805	0.009376	0.0073194	0.0055741	0.005976	0.0060525	0.0049758
3-hydroxypropionic acid	0.005050782	0.005872063	0.006048492	0.00648562	0.005705451	0.006081869	0.006924237	0.007754804	0.008498626	0.00786468	0.00860634	0.008355691	0.036721	0.0285466	0.021546	0.024605	0.0244389	0.0200703
Melibiose	0.00730195	0.008481656	0.012255954	0.010953759	0.013620169	0.01479097	0.015713128	0.020668194	0.023094263	0.029423201	0.039280984	0.040880857	0.074851	0.0705488	0.0641668	0.066561	0.0618933	0.0697318
1-methyladenosine	0.001594943	0.000982725	0.001336091	0.000923974	0.000615713	0.000347876	0.000102878	9.76721E-05	9.64464E-05	0.00012076	9.16241E-05	7.8615E-05	0.000148	7.929E-05	0.0001679	6.72E-05	0.0002176	6.478E-05
Sorbitol	0.007561776	0.014240762	0.007697817	0.010591316	0.010759289	0.007374394	0.006242778	0.008385433	0.007756664	0.007904184	0.009955024	0.008316854	0.031596	0.0251586	0.0338425	0.030856	0.0249426	0.0248887
1,5-anhydroglucitol	0.000155535	0.000130301	0.000169002	0.00021619	0.000158038	0.000106835	0.001014352	0.001073535	0.001522121	0.000788513	0.000568603	0.00051258	0.00025	0.0002363	0.0002929	0.000243	0.0002223	0.0001897
Sinapinic acid	0.028498827	0.03662099	0.042401632	0.041025543	0.033992585	0.058075891	0.026625015	0.028933876	0.040739359	0.032542617	0.034996465	0.035405334	0.122252	0.1115213	0.1027952	0.102117	0.0887584	0.0977442
L-phenylalanine	0.181134858	0.173829493	0.208112865	0.195840663	0.157014657	0.092922584	0.05439955	0.060642753	0.09089106	0.066309682	0.071315082	0.069114493	0.040963	0.0322486	0.0355709	0.033052	0.0280933	0.0246401
Adenine	0.009737313	0.012696835	0.012172604	0.014023054	0.01552065	0.01818181	0.057091035	0.079821524	0.090827004	0.069489978	0.09020981	0.098470024	0.031386	0.0256859	0.0216881	0.028968	0.0341216	0.0412423
N-acetylputrescine	0.040571686	0.054772124	0.05924057	0.056230502	0.067028247	0.101346126	0.024898028	0.035520869	0.04697663	0.101296909	0.146072216	0.155031452	0.24111	0.2470444	0.2010425	0.206136	0.2159828	0.2068118
Valeric acid	0.041201318	0.037831506	0.041138188	0.041949519	0.033111644	0.024134175	0.005116755	0.005685451	0.007410892	0.005311181	0.00539338	0.005049268	0.011516	0.0107133	0.0108096	0.011217	0.0099835	0.0107703
Ethylmorphine	0.300551312	0.242274996	0.222003226	0.276044117	0.165749878	0.103855464	0.020713264	0.025174711	0.028358553	0.026256032	0.050882015	0.033474479	0.04798	0.0613983	0.0584355	0.054	0.0565572	0.0600747
4-hydroxybenzeneacetic acid	0.001388116	0.001515865	0.001612179	0.001417252	0.001428817	0.001484121	0.000287555	0.000360565	0.000397519	0.000369178	0.000343317	0.000290012	0.00132	0.0010837	0.0009858	0.000901	0.0009235	0.0007993
D-mannose	2.417966675	2.17738305	4.122447014	2.493934502	3.029419907	1.833344699	0.37750706	0.510761465	0.474240791	0.223219979	0.396201912	0.207014013	2.243209	0.7686391	0.6449861	0.223093	2.6656995	2.1080998
Beta-mannosylglycerate	0.00358611	0.003314249	0.002484014	0.002240321	0.001790229	0.001442642	0.001789369	0.002456923	0.002442369	0.002962245	0.002211636	0.002384338	0.001002	0.0007458	0.0008428	0.000789	0.0009305	0.0007462
2-hydroxypentanoic acid	0.015757958	0.014157034	0.0162261	0.015224853	0.012024059	0.008464312	0.003803722	0.003772167	0.005796878	0.003432451	0.004340867	0.004103803	0.015107	0.0137695	0.013595	0.01337	0.0108175	0.011439
Chlorogenic acid	0.002043346	0.002212777	0.0026211	0.002330818	0.0029478	0.004400948	0.000860911	0.001202531	0.001070921	0.001404991	0.001982136	0.001975236	0.005357	0.0043175	0.0042209	0.004219	0.0038738	0.0042358
Myo-inositol	0.042703274	0.030917122	0.030440671	0.029621644	0.018315717	0.008533265	0.003040126	0.003886164	0.003292077	0.003657875	0.003949839	0.005115472	0.012055	0.007561	0.0065721	0.006462	0.0065848	0.0071301
3-deoxyhexitol	0.003978066	0.006015022	0.005871482	0.005789405	0.006524272	0.009644121	0.005444907	0.005590144	0.006314882	0.008442494	0.010967666	0.011957521	0.02577	0.0213163	0.0182157	0.019018	0.0183057	0.0169484
2-hydroxy-3-methylpentanoic aci	0.001179785	0.001298202	0.001356803	0.001404245	0.001713872	0.002172211	0.005130773	0.004970905	0.006390714	0.006940534	0.007915862	0.007985124	0.007917	0.0078719	0.0070095	0.006511	0.0060919	0.0060165
2-deoxyerythritol	7.14842E-05	9.28279E-05	0.00012431	8.61575E-05	0.000116952	9.00395E-05	0.000161267	0.000182697	0.000251787	0.000323776	0.000410902	0.000378472	0.000566	0.0004378	0.0004445	0.000404	0.000385	0.0003648
L-lactic acid	0.257465999	0.326026325	0.324633091	0.333052621	0.544559583	0.744835646	1.737679864	2.004081717	1.968758295	2.053580943	2.185856633	2.16090024	1.819921	1.6990402	1.5636833	1.571895	1.5387048	1.6644071
Gluconic acid	0.147830067	0.14267184	0.152253257	0.143489602	0.190045396	0.257789812	0.683427204	1.045307402	0.947981526	0.959568522	1.007581752	0.952342278	0.418784	0.5095895	0.37752	0.568267	0.5678323	0.9100885
Phenylacetic acid	0.003893189	0.004039456	0.005257171	0.0050123	0.005009901	0.004305551	0.000565048	0.000564308	0.000850091	0.000926542	0.001258261	0.001237465	0.001554	0.0013428	0.0012882	0.001319	0.001269	0.0012671
Deoxycholic acid	0.000620771	0.000501466	0.000531181	0.000562217	0.00059668	0.000567627	0.002552523	0.002828017	0.003595171	0.002698149	0.003000459	0.002261662	0.002367	0.0017392	0.0014893	0.001516	0.0019589	0.0015829
Lactobionic acid	0.000464312	0.000426341	0.000652994	0.000583319	0.000369553	0.000376834	0.000757558	0.000630366	0.000887407	0.000912545	6.77533E-05	0.001004748	0.002326	0.0019169	0.0014234	0.001811	0.0013981	0.0013562
N-carbamoylaspartate	0.001865898	0.003479367	0.002769032	0.002282283	0.00241828	0.004476912	0.001667979	0.001636979	0.001974043	0.002571584	0.002942206	0.003351061	0.010612	0.0076704	0.0052388	0.006327	0.0063551	0.0041885
L-glutamine dehydrated	0.005238743	0.00703647	0.009228942	0.008362726	0.008622281	0.006580317	0.002938746	0.003935934	0.005037834	0.003038657	0.00361085	0.002980524	0.001525	0.0018064	0.0025697	0.002202	0.0017948	0.001716
Isochlorogenic acid	0.0151831	0.007622088	0.006743986	0.006301582	0.00372909	0.001948344	0.001173162	0.001426087	0.001078965	0.001044824	0.00146817	0.001494169	0.001812	0.0012355	0.000973	0.001286	0.0009603	0.0010106
Pyruvic acid	0.002262813	0.002364492	0.002157043	0.002214471	0.003059073	0.003574242	0.0146138	0.013360862	0.017716927	0.012112259	0.010882448	0.009720341	0.009645	0.0080227	0.0071421	0.008556	0.0082983	0.0108534
2-hydroxy-3-methylbutyric acid	0.003011808	0.003572095	0.003841712	0.003707452	0.004893091	0.007161784	0.012150036	0.012660952	0.016473461	0.017034693	0.018412653	0.018217487	0.019309	0.0182574	0.0171222	0.016829	0.0168746	0.0166119
3-desoxy-pentitol	0.031474267	0.029591237	0.034386468	0.034365044	0.029859214	0.023652691	0.010471947	0.011859666	0.016111738	0.010223367	0.012353169	0.011598663	0.035543	0.0308411	0.0329979	0.032623	0.0286934	0.0304983
L-tyrosine	5.584245881	5.295052515	6.468118871	5.830610335	4.325010771	2.79508639	1.279307434	1.582937146	2.178265853	1.516720051	1.70485896	1.619865042	1.614138	1.2527276	1.2560397	1.168576	0.9960224	0.8558444
Galactitol	0.012062451	0.011684062	0.014449309	0.014041874	0.013020368	0.011350551	0.004883404	0.005021381	0.007953921	0.006043811	0.0080057	0.007108977	0.021292	0.0171383	0.0168897	0.016194	0.0142504	0.0159499
Dl-dopa	0.09752972	0.11527998	0.161153372	0.147946377	0.243859361	0.224426416	0.16729288	0.168993099	0.143347441	0.11603527	0.253720784	0.250769453	0.083527	0.0622798	0.0721414	0.075223	0.07442	0.0675362
2-ketobutyric acid	0.002040499	0.001830181	0.002033041	0.001562764	0.001625564	0.001427447	0.001901621	0.002259873	0.002697584	0.002088562	0.002142502	0.002065881	0.001087	0.0009789	0.0009812	0.000823	0.0008406	0.0009967
Malate	0.036462479	0.028875623	0.025924426	0.024500795	0.018061785	0.018447451	0.027890496	0.031167711	0.032909901	0.024967074	0.023326062	0.02326403	0.014102	0.0100624	0.0092871	0.01168	0.0123336	0.0120457
Benzoic acid	0.003189481	0.003346684	0.003387408	0.003423561	0.002811872	0.002411125	0.00599423	0.005068468	0.006406565	0.003714857	0.003487075	0.003182465	0.002113	0.0018606	0.0016524	0.001886	0.0017416	0.0017059
Benzenepropanoic acid	0.026029603	0.024644207	0.028658322	0.028414219	0.020896587	0.015311894	0.004129505	0.004537927	0.0064598	0.005537777	0.006876499	0.006543117	0.008912	0.0079047	0.0081057	0.008159	0.007595	0.0083791
2-hydroxy-2-phenylpropanoic aci	0.001286799	0.001468993	0.001611618	0.001646157	0.001999672	0.002289371	0.005052091	0.005163388	0.006197079	0.006981475	0.007485951	0.007484398	0.004075	0.00383	0.0037994	0.003596	0.003518	0.0035088
Serotonin	0.0584873	0.05874797	0.063604442	0.055678654	0.038248628	0.028266238	0.010271217	0.011038297	0.014943169	0.012548703	0.015871864	0.01633523	0.022215	0.0151693	0.0141613	0.013652	0.0125466	0.0118218
Homoserine	0.006181426	0.006562473	0.007309229	0.006884119	0.006315547	0.006776861	0.002123172	0.002321573	0.003228348	0.002835673	0.003382037	0.003269233	0.007794	0.0065633	0.0059615	0.006596	0.0062271	0.0062542
4-hydroxyhippuric acid	0.003086149	0.002961757	0.003242647	0.003295306	0.003022561	0.002093861	0.003657116	0.003996197	0.004574258	0.004255524	0.004591123	0.004413632	0.008375	0.0076735	0.0077569	0.007462	0.0072404	0.0074099
Lactitol	0.001147998	0.000729067	0.000500821	0.000494751	0.000367527	0.000354067	0.000359179	0.000353362	0.000394487	0.000523179	0.000477138	0.000453504	0.000252	0.0002175	0.0001843	0.000197	0.0002048	0.0001883
Creatinine	0.001227878	0.001733436	0.002438345	0.001993959	0.002327943	0.001665993	0.001143384	0.001273099	0.001019902	0.001325007	0.001608505	0.001437526	0.004831	0.0038916	0.0024565	0.002804	0.0027395	0.0024192
3-methyl-2-oxovaleric acid	0.000599706	0.000499346	0.000538023	0.000517374	0.000408487	0.000281532	0.000197176	0.000149013	0.0002669	0.00014663	0.000174908	0.00014027	0.000175	0.0001084	0.0001418	0.000126	0.0001294	0.0001288
Agmatine	0.012202036	0.006973814	0.00866263	0.009547931	0.005374633	0.034782502	0.001440184	0.001878771	0.001225898	0.002029664	0.005813217	0.004069544	0.011085	0.0080136	0.0042413	0.003562	0.0028909	0.0032404
Piceatannol	0.000976498	0.00218328	0.002938024	0.001002949	0.002418241	0.00190542	0.001479401	0.001385341	0.001412877	0.001162135	0.002602131	0.00150338	0.005669	0.0039767	0.0032634	0.003539	0.0032909	0.0029663
Guanine	0.067605653	0.060438692	0.088898477	0.069179187	0.041236856	0.025524653	0.010357562	0.015230761	0.017164797	0.015501178	0.01589964	0.015261571	0.021204	0.0184386	0.014784	0.019875	0.0162971	0.0182272
Phenylethylamine	0.004161334	0.002352899	0.002244177	0.002095737	0.001935996	0.002363567	0.001127326	0.001215212	0.001702764	0.002066593	0.002406614	0.002033227	0.008259	0.0062038	0.0039599	0.003244	0.0031725	0.0026344
Erythronic acid	0.000227102	0.000239174	2.06558E-05	0.000228237	0.00022522	0.000154571	0.000335415	0.000308082	0.000421111	0.000303403	0.000242719	6.37672E-06	0.000717	0.0005769	0.0004247	0.000448	0.000484	0.0004122
Uric acid	0.006196106	0.005634069	0.005647853	0.006091924	0.005226452	0.005639077	0.00987029	0.01007366	0.013117543	0.010722081	0.011798578	0.01232579	0.006471	0.0053898	0.0048104	0.005574	0.0051072	0.005093
L-methionine	0.174794887	0.178958072	0.21341108	0.195613053	0.150849291	0.115320155	0.046291332	0.052648439	0.073998716	0.057789005	0.069564552	0.069052095	0.06671	0.05825	0.056061	0.054811	0.0481815	0.0460239
3-(4-hydroxyphenyl)propionic aci	0.008128622	0.008531186	0.009500558	0.00890571	0.007227314	0.006876521	0.008036059	0.008235335	0.009531013	0.008137892	0.008023412	0.00751298	0.025413	0.0198104	0.016072	0.0153	0.0142496	0.0114637
Aspartate	0.00135553	0.001051163	0.001083443	0.000957061	0.000483223	0.000208619	0.00019916	0.000247928	0.000289997	0.000145768	0.000194136	0.000166692	0.000883	0.0007203	8.051E-05	0.000783	0.0007254	0.0007939
Resveratrol	0.035303012	0.030408327	0.029920115	0.025887752	0.017451062	0.012525742	0.005714708	0.007423978	0.00872196	0.008241081	0.009955075	0.009960893	0.009791	0.0086029	0.0075548	0.007316	0.0067599	0.0062049
1,3-diaminopropane	0.002792493	0.003495107	0.004211098	0.003944417	0.004247804	0.004715714	0.003017507	0.00310465	0.004293619	0.00432969	0.004834012	0.004900162	0.008104	0.0070147	0.0080443	0.008031	0.0072747	0.0085336
Pseudo uridine	0.591688215	0.390847869	0.391090277	0.345375328	0.173067845	0.135010513	0.075523139	0.076167249	0.092299687	0.079570399	0.085659344	0.078308953	0.157587	0.1117982	0.103265	0.106592	0.0118864	0.0903309
Leucinic acid	0.002343678	0.002901235	0.002820656	0.002864038	0.004159933	0.005689849	0.008946013	0.010118877	0.012042917	0.01079868	0.011332278	0.011071759	0.007807	0.0074791	0.0075077	0.006831	0.0063919	0.0066512
2-aminoethyl methacrylate	0.143843802	0.133037115	0.149763207	0.134727534	0.091145306	0.070593915	0.026819957	0.030577406	0.0393805	0.034397438	0.044433199	0.043393016	0.061249	0.0489265	0.0424331	0.044829	0.0384156	0.0362633
5-hydroxyindole	0.086079044	0.08557011	0.100475715	0.092439327	0.07910078	0.075201212	0.064850354	0.064766494	0.106573349	0.062122015	0.061932431	0.055532434	0.045715	0.034953	0.0359132	0.035224	0.0357484	0.0421611
Galactonic acid	0.007371154	0.008146537	0.010049729	0.009204447	0.012526815	0.014649959	0.025062013	0.028280543	0.034581817	0.029044374	0.035933244	0.03497572	0.026657	0.0217487	0.019596	0.020032	0.0205012	0.0231966
Desyrel	0.00373461	0.002215342	0.003589236	0.001520179	0.00517611	0.005143456	0.000506503	0.001883996	0.000440819	0.001153473	0.001877284	0.00144882	0.000719	0.0007044	0.0011209	0.001151	0.001046	0.0015506
1-kestose	0.131903784	0.114568261	0.131780995	0.115890934	0.080546624	0.186716024	0.036179227	0.043794606	0.047161062	0.038664037	0.060360164	0.049690174	0.127528	0.1058705	0.0793604	0.073149	0.0900994	0.0781487
Lithocholic acid	0.001209998	0.001094757	0.001277854	0.001325077	0.001229606	0.001393167	0.000667527	0.000745834	0.000878309	0.000903467	0.001126949	0.000967393	0.002101	0.0017306	0.0016701	0.001895	0.0015341	0.0014896
5-hydroxy-3-indoleacetic acid	0.003036691	0.003094592	0.003045284	0.002712418	0.002110527	0.002021174	0.006478995	0.006514648	0.007508384	0.005339428	0.004148836	0.00442614	0.004106	0.0027011	0.0023475	0.002754	0.0025849	0.0026966
5-aminovaleric acid	0.074853202	0.097059475	0.104952703	0.101032784	0.117912853	0.16044874	0.20544031	0.246522676	0.333652603	0.28946909	0.314323714	0.30065289	0.303396	0.274168	0.2774571	0.266744	0.2518899	0.2510403
L-tryptophan	0.353603338	0.376180979	0.521288928	0.419746924	0.358835162	0.274034307	0.078721115	0.135135245	0.182964999	0.15095581	0.205631043	0.215374007	0.209693	0.1530621	0.1234439	0.138915	0.1236665	0.117711
Maltotriose	0.134436368	0.113451968	0.136183394	0.117120523	0.091433279	0.068905499	0.041025161	0.046251621	0.057439234	0.038664037	0.057597065	0.055269591	0.126287	0.1048085	0.0938057	0.093519	0.0898349	0.077789
3-methoxy-5-prop-2-en- 1-yl-2-hy	0.008621146	0.009747927	0.014022245	0.011617287	0.013182051	0.011675928	0.003809775	0.004071284	0.007506265	0.005221314	0.008820764	0.008203309	0.005072	0.0048495	0.0049011	0.004447	0.0038873	0.0039096
N-acetylglutamate	0.000532031	0.000471196	0.000487032	0.000431781	0.000418479	0.000395493	0.000678547	0.000866385	0.000957259	0.000699017	0.000720287	0.000621038	0.000542	0.0004207	0.0003781	0.000386	0.0003467	0.0003079
L-glutamic acid	0.350437713	0.313747134	0.340402903	0.29885586	0.217235755	0.1869495	0.126709949	0.127137029	0.154781402	0.127321864	0.140699567	0.13394694	0.151882	0.1202548	0.1038455	0.104446	0.0944756	0.0849103
Tromethamine	0.099568281	0.097447928	0.114511508	0.09807392	0.085852926	0.083943889	0.029792294	0.028598794	0.043089887	0.038750402	0.048897897	0.045503548	0.099878	0.0709831	0.0566944	0.06821	0.0581243	0.0606607
4-hydroxyproline	0.005143472	0.005408325	0.00573326	0.006127207	0.005917193	0.006799167	0.009951555	0.008806546	0.012590416	0.010469155	0.010791452	0.011236518	0.017995	0.0154184	0.0121175	0.013269	0.0133695	0.0113997
Pantothenic acid	0.03423308	0.034378109	0.034545414	0.029290586	0.018194249	0.019899241	0.003293936	0.013431524	0.004938576	0.014718098	0.015319013	0.013112997	0.025894	0.0204232	0.0175227	0.017558	0.0146512	0.0134463
Hexadecylamine	0.027674219	0.03894426	0.026727542	0.036954785	0.017267847	0.016994529	0.008752778	0.010214374	0.012084728	0.01122371	0.029429937	0.022779962	0.01165	0.0111577	0.0073494	0.007071	0.0108265	0.0139322
3-hydroxyphenylacetic acid	0.002384986	0.002107684	0.00219008	0.002225321	0.001721866	0.001271548	0.001273041	0.001276834	0.001558671	0.001256303	0.001277314	0.001157337	0.000965	0.0008799	0.0008611	0.000853	0.0008617	0.000861
4-hydroxybutyric acid	0.001326411	0.003602267	0.00433467	0.004030981	0.004049845	0.004399502	0.007044136	0.00741	0.008730189	0.008615579	0.009422812	0.009184466	0.010899	0.0075524	0.0089174	0.008994	0.007008	0.0076239
2-aminoisobutyric acid	0.017750935	0.017238399	0.01977174	0.019030816	0.017752828	0.01576637	0.010063915	0.01146702	0.014732349	0.012868812	0.014466124	0.014043123	0.029542	0.0255167	0.0220052	0.022062	0.0214593	0.0200077
Guanosine	0.002842422	0.00330155	0.003877495	0.002932718	0.002964749	0.00343634	0.005822308	0.006589026	0.008551769	0.006533174	0.006644052	0.006035795	0.009868	0.0072293	0.0071887	0.006963	0.0064034	0.006758
Melezitose	0.114087767	0.036931965	0.098354325	0.102336485	0.072118785	0.031795634	0.016651169	0.026005713	0.028036026	0.021838542	0.029391181	0.028459399	0.036808	0.0497295	0.0397284	0.060073	0.0398305	0.0465348
Dehydroascorbic acid	0.000303658	0.000329102	0.000314114	0.000361092	0.00028353	0.00022999	0.000291087	0.000318495	0.000394651	0.000294992	0.000251782	0.000253703	0.000145	0.0001671	0.0001732	0.000204	0.0001721	0.0001834
3-(3-hydroxyphenyl)propionic aci	0.12455582	0.126055285	0.15253752	0.15076628	0.153853145	0.133446065	0.222037441	0.226786672	0.288214251	0.233683458	0.240607656	0.228780451	0.1449	0.1297438	0.1415305	0.136649	0.1324473	0.141457
Oxoproline	0.618372425	0.592194198	0.664385085	0.61030965	0.428413384	0.371766968	0.243965827	0.274898898	0.320497998	0.282558763	0.313505782	0.304906182	0.287592	0.242344	0.2294048	0.236605	0.2193105	0.1991787
Catechollactate	0.003613892	0.004384341	0.003909897	0.005729686	0.006346057	0.008324087	0.007780217	0.006988053	0.010114495	0.015518615	0.022009038	0.022688343	0.012091	0.0108728	0.0110075	0.011581	0.011659	0.0131765
