# Supplementary material for: Sodium butyrate mediates histone crotonylation and alleviated neonatal rats hypoxic–ischemic brain injury through gut–brain axis
Source: Front Microbiol. 2022 Oct 20;13:993146. doi: 10.3389/fmicb.2022.993146 (PMC9631217; doi:10.3389/fmicb.2022.993146)
Supplement: Supplementary file 1 [file Data_Sheet_1.ZIP › Supplementary Table/Table S3.pdf]

Table S3 Community structure of the top 15 species in order of abundance at the family level

Taxonomy	Sham1	Sham2	Sham3	HIBD1	HIBD2	HIBD3	HIBD+SB1	HIBD+SB2	HIBD+SB3
Prevotellaceae	0.311777949	0.471651164	0.425380651	0.018366017	0.003228864	0.058445406	0.499052077	0.418093489	0.385952959
Muribaculaceae	0.136589845	0.030422418	0.101961017	0.079062741	0.117838735	0.16867113	0.095236685	0.103886486	0.054564844
Lachnospiraceae	0.121956277	0.221103146	0.171633391	0.041205048	0.009716215	0.069524261	0.166419812	0.123615143	0.214823153
Bacteroidaceae	0.176106404	0.029622608	0.013596777	0.072397654	0.048758813	0.145032289	0.053824279	0.065110492	0.012145269
Lactobacillaceae	0.03077789	0.059778423	0.019787902	0.138811541	0.144499082	0.203033355	0.027667516	0.068220866	0.070738788
Enterobacteriaceae	0.017980923	0.002221696	0.001333017	0.425499141	0.440310445	0.056845785	0.001984715	0.001569998	0.001273772
Ruminococcaceae	0.082054624	0.114817229	0.1783281	0.046714853	0.056964275	0.124474199	0.056490313	0.086764619	0.098969133
Tannerellaceae	0.019047337	0.00284377	0.005776409	0.088304994	0.064992002	0.047159192	0.010634516	0.048225606	0.005657918
Rikenellaceae	0.029859589	0.012204514	0.010664139	0.019580544	0.028437704	0.053024468	0.024260916	0.02192073	0.0136264
Marinifilaceae	0.019225073	0.008916405	0.006220748	0.008709047	0.014752059	0.007879614	0.009064518	0.010427158	0.003850939
Clostridiales_vadinBB60_group	0.002192073	0.017003377	0.009805083	0.000177736	0.000148113	0.000770188	0.02055809	0.005391315	0.023253747
Erysipelotrichaceae	0.004295278	0.002547544	0.001984715	0.010930742	0.023224125	0.005835654	0.000947923	0.000710943	0.001273772
Pasteurellaceae	0.005628296	0.000829433	0.000473962	0.00613188	0.000740565	0.002429054	0.001806979	0.00192547	0.00284377
Desulfovibrionaceae	0.000947923	0.004532259	0.018958469	0.003702826	0.000859056	0.011138101	0.003762071	0.003613958	0.02328337
Deferribacteraceae	2.96E-05	0	0.003910184	0	0	0	0.004828485	0	0.058563896
