# Supplementary material for: Sodium butyrate mediates histone crotonylation and alleviated neonatal rats hypoxic–ischemic brain injury through gut–brain axis
Source: Front Microbiol. 2022 Oct 20;13:993146. doi: 10.3389/fmicb.2022.993146 (PMC9631217; doi:10.3389/fmicb.2022.993146)
Supplement: Supplementary file 1 [file Data_Sheet_1.ZIP › Supplementary Table/Table S1.pdf]

Table S1 KEGG pathway in Sham and HIBD groups

| Metabolite name | KEGG | P-value | FC | average(HIBD) | average(Sham) | Sham-1 | Sham-2 | Sham-3 | Sham-4 | Sham-5 | Sham-6 | HIBD-1 | HIBD-2 | HIBD-3 | HIBD-4 | HIBD-5 | HIBD-6 |
|---|---|---|---|---|---|---|---|---|---|---|---|---|---|---|---|---|---|
| L-glutamic acid | Butanoate metabolism | 0.00031123 | 0.474691408 | 0.135099458 | 0.284604811 | 0.350437713 | 0.313747134 | 0.340402903 | 0.29885586 | 0.217235755 | 0.1869495 | 0.126709949 | 0.127137029 | 0.154781402 | 0.127321864 | 0.140699567 | 0.13394694 |
| (r)-3-hydroxybutyric acid | Butanoate metabolism | 7.60382E-08 | 0.107864214 | 0.000312402 | 0.002896255 | 0.002913297 | 0.002921097 | 0.003485724 | 0.003171681 | 0.002690987 | 0.002194741 | 0.000163475 | 0.000171735 | 0.000309076 | 0.000302339 | 0.000448204 | 0.000479584 |
| Succinic acid | Butanoate metabolism | 1.17842E-10 | 0.097566025 | 0.111475228 | 1.142561957 | 1.001302382 | 1.100802304 | 1.276055261 | 1.159273275 | 1.127619433 | 1.190319087 | 0.077474891 | 0.10171461 | 0.12195119 | 0.113258235 | 0.124226839 | 0.130225606 |
| Pyruvic acid | HIF-1 signaling pathway | 5.19891E-06 | 5.015734525 | 0.013067773 | 0.002605356 | 0.002262813 | 0.002364492 | 0.002157043 | 0.002214471 | 0.003059073 | 0.003574242 | 0.0146138 | 0.013360862 | 0.017716927 | 0.012112259 | 0.010882448 | 0.009720341 |
| L-lactic acid | HIF-1 signaling pathway | 2.04092E-08 | 4.785815872 | 2.018476282 | 0.421762211 | 0.257465999 | 0.326026325 | 0.324633091 | 0.333052621 | 0.544559583 | 0.744835646 | 1.737679864 | 2.004081717 | 1.968758295 | 2.053580943 | 2.185856633 | 2.16090024 |
| Myristic acid | Fatty acid biosynthesis | 0.000208839 | 0.493133152 | 0.024500932 | 0.049684212 | 0.0625756 | 0.055060609 | 0.053770936 | 0.051272391 | 0.038837608 | 0.036588127 | 0.021553863 | 0.021708082 | 0.020952869 | 0.023350509 | 0.031307453 | 0.028132816 |
| Pantothenic acid | Pantothenate and CoA biosynthesis | 0.000854407 | 0.380050924 | 0.010802357 | 0.028423446 | 0.03423308 | 0.034378109 | 0.034545414 | 0.029290586 | 0.018194249 | 0.019899241 | 0.003293936 | 0.013431524 | 0.004938576 | 0.014718098 | 0.015319013 | 0.013112997 |
| L-aspartic acid | Pantothenate and CoA biosynthesis | 0.002706972 | 0.305786668 | 0.068893693 | 0.225299859 | 0.287624009 | 0.258496613 | 0.316592399 | 0.270324489 | 0.14620381 | 0.072557833 | 0.064219662 | 0.078666078 | 0.103970711 | 0.054467728 | 0.06269118 | 0.0493468 |
| Aspartate | Pantothenate and CoA biosynthesis | 0.00408323 | 0.242006631 | 0.00020728 | 0.000856506 | 0.00135553 | 0.001051163 | 0.001083443 | 0.000957061 | 0.000483223 | 0.000208619 | 0.00019916 | 0.000247928 | 0.000289997 | 0.000145768 | 0.000194136 | 0.000166692 |
| L-glutamine | Purine metabolism | 3.19423E-05 | 0.240982913 | 0.007885807 | 0.032723511 | 0.029757487 | 0.035561035 | 0.044318503 | 0.036186564 | 0.030641926 | 0.01987555 | 0.007402671 | 0.009353429 | 0.012256107 | 0.005792481 | 0.006789927 | 0.005720227 |
| Hypoxanthine | Purine metabolism | 0.002717141 | 0.511443665 | 0.093145834 | 0.18212335 | 0.236706727 | 0.214193553 | 0.219711442 | 0.191633227 | 0.12543881 | 0.105056343 | 0.076908252 | 0.085769835 | 0.098281688 | 0.094841459 | 0.106429204 | 0.096644564 |
| Guanine | Purine metabolism | 0.000751263 | 0.253385335 | 0.014902585 | 0.05881392 | 0.067605653 | 0.060438692 | 0.088898477 | 0.069179187 | 0.041236856 | 0.025524653 | 0.010357562 | 0.015230761 | 0.017164797 | 0.015501178 | 0.01589964 | 0.015261571 |
| Uric acid | Purine metabolism | 1.41992E-06 | 1.972033972 | 0.01131799 | 0.005739247 | 0.006196106 | 0.005634069 | 0.005647853 | 0.006091924 | 0.005226452 | 0.005639077 | 0.00987029 | 0.01007366 | 0.013117543 | 0.010722081 | 0.011798578 | 0.01232579 |
| Guanosine | Purine metabolism | 1.01722E-05 | 2.075719863 | 0.006696021 | 0.003225879 | 0.002842422 | 0.00330155 | 0.003877495 | 0.002932718 | 0.002964749 | 0.00343634 | 0.005822308 | 0.006589026 | 0.008551769 | 0.006533174 | 0.006644052 | 0.006035795 |
| Adenine | Purine metabolism | 1.02816E-06 | 5.901809799 | 0.080984896 | 0.013722044 | 0.009737313 | 0.012696835 | 0.012172604 | 0.014023054 | 0.01552065 | 0.01818181 | 0.057091035 | 0.079821524 | 0.090827004 | 0.069489978 | 0.09020981 | 0.098470024 |
| L-glutamic acid | Neuroactive ligand-receptor interaction | 0.00031123 | 0.474691408 | 0.135099458 | 0.284604811 | 0.350437713 | 0.313747134 | 0.340402903 | 0.29885586 | 0.217235755 | 0.1869495 | 0.126709949 | 0.127137029 | 0.154781402 | 0.127321864 | 0.140699567 | 0.13394694 |
| L-aspartic acid | Neuroactive ligand-receptor interaction | 0.002706972 | 0.305786668 | 0.068893693 | 0.225299859 | 0.287624009 | 0.258496613 | 0.316592399 | 0.270324489 | 0.14620381 | 0.072557833 | 0.064219662 | 0.078666078 | 0.103970711 | 0.054467728 | 0.06269118 | 0.0493468 |
| Serotonin | Neuroactive ligand-receptor interaction | 7.99482E-05 | 0.267325399 | 0.013501413 | 0.050505539 | 0.0584873 | 0.05874797 | 0.063604442 | 0.055678654 | 0.038248628 | 0.028266238 | 0.010271217 | 0.011038297 | 0.014943169 | 0.012548703 | 0.015871864 | 0.01633523 |
| Adrenaline | Neuroactive ligand-receptor interaction | 7.50594E-06 | 0.398503009 | 0.001035914 | 0.002599514 | 0.002826927 | 0.00270228 | 0.003048955 | 0.002737944 | 0.002335921 | 0.00194506 | 0.000764487 | 0.000797074 | 0.001291068 | 0.001266046 | 0.001001644 | 0.001095165 |
| D-ribose | Pentose phosphate pathway | 0.000485698 | 0.340262482 | 0.000532341 | 0.0015645 | 0.001531365 | 0.001716921 | 0.002356438 | 0.001216226 | 0.001382397 | 0.001183653 | 0.000572056 | 0.000794673 | 0.000736339 | 0.000428843 | 0.000542282 | 0.000119851 |
| D-ribulose 5-phosphate | Pentose phosphate pathway | 0.005385737 | 0.510043686 | 0.004971106 | 0.009746431 | 0.01397216 | 0.010174414 | 0.009882014 | 0.011702544 | 0.007738795 | 0.00500866 | 0.003720754 | 0.004363173 | 0.004580067 | 0.004425454 | 0.006509286 | 0.006227901 |
| Gluconic acid | Pentose phosphate pathway | 8.19521E-08 | 5.411775518 | 0.932701447 | 0.172346662 | 0.147830067 | 0.14267184 | 0.152253257 | 0.143489602 | 0.190045396 | 0.257789812 | 0.683427204 | 1.045307402 | 0.947981526 | 0.959568522 | 1.007581752 | 0.952342278 |
| L-tryptophan | Glycine, serine and threonine metabolism | 0.000217628 | 0.420535043 | 0.161463703 | 0.383948273 | 0.353603338 | 0.376180979 | 0.521288928 | 0.419746924 | 0.358835162 | 0.274034307 | 0.078721115 | 0.135135245 | 0.182964999 | 0.15095581 | 0.205631043 | 0.215374007 |
| Serine | Glycine, serine and threonine metabolism | 6.44568E-05 | 0.506996619 | 0.230683408 | 0.454999893 | 0.466370804 | 0.477294418 | 0.535317305 | 0.492274902 | 0.414895996 | 0.343845933 | 0.16018014 | 0.180885276 | 0.269321596 | 0.225730411 | 0.273834745 | 0.274148277 |
| L-threonine | Glycine, serine and threonine metabolism | 4.50221E-05 | 0.503797397 | 0.104960571 | 0.208338852 | 0.202691014 | 0.213488624 | 0.248011212 | 0.22666676 | 0.195758173 | 0.163417329 | 0.073255964 | 0.082016457 | 0.128498029 | 0.10128373 | 0.122225794 | 0.122483453 |
| Homoserine | Glycine, serine and threonine metabolism | 7.1762E-08 | 0.428683109 | 0.002860006 | 0.006671609 | 0.006181426 | 0.006562473 | 0.007309229 | 0.006884119 | 0.006315547 | 0.006776861 | 0.002123172 | 0.002321573 | 0.003228348 | 0.002835673 | 0.003382037 | 0.003269233 |
| L-cystathionine | Glycine, serine and threonine metabolism | 6.44568E-05 | 0.506996619 | 0.230683408 | 0.454999893 | 0.466370804 | 0.477294418 | 0.535317305 | 0.492274902 | 0.414895996 | 0.343845933 | 0.16018014 | 0.180885276 | 0.269321596 | 0.225730411 | 0.273834745 | 0.274148277 |
