# Supplementary material for: Sodium butyrate mediates histone crotonylation and alleviated neonatal rats hypoxic–ischemic brain injury through gut–brain axis
Source: Front Microbiol. 2022 Oct 20;13:993146. doi: 10.3389/fmicb.2022.993146 (PMC9631217; doi:10.3389/fmicb.2022.993146)
Supplement: Supplementary file 1 [file Data_Sheet_1.ZIP › Supplementary Table/Table S5.pdf]

Table S5 Differentially expressed genes in HIBD+SB and HIBD group

Gene	Pvalue	regulation	HIBD+SB	HIBD
Slc1a3	3.96949E-36	down	0.0037	151.11305
Gabbr1	3.47975E-28	down	0.27715	176.41099
Prpf8	9.84667E-23	down	0	26.70323
Nsmf	1.75416E-20	down	0	56.78704
Slc23a2	6.38241E-18	down	0	19.93184
Stmn1	2.38226E-17	down	0	112.2474
Ankrd11	2.67236E-16	down	0.00315	13.76452
Mycbp2	2.39019E-15	down	0	12.49787
Dlg3	1.27471E-13	down	0.0038	22.7122
L1cam	1.34131E-13	down	0	14.95248
AABR0707004	8.62103E-12	down	1.25571	157.33983
Idh3g	1.47712E-11	down	0.0835	63.80711
Arpc5l	2.3612E-11	down	0	42.53452
Utrn	2.5573E-11	down	0.00225	5.19111
Sorcs2	4.70512E-11	down	0	8.67648
Slc9a6	1.84616E-10	down	0.17396	26.89067
Atrx	2.10113E-10	down	0.01771	7.86987
Sccpdh	3.74458E-10	down	0	23.26509
Rpl3	6.34555E-10	down	2.90924	160.69774
Golga4	1.14511E-09	down	0.01572	8.90734
Hiatl3	2.87172E-09	down	0	13.21873
Flnb	7.21412E-09	down	0	3.70536
RragB	7.36853E-09	down	0	8.35748
Fbxl3	8.22731E-09	down	0	9.43969
Hnrnpr	1.32596E-08	down	0	11.69359
Tlk1	1.60124E-08	down	0	8.04725
Cryzl1	1.82175E-08	down	0	16.25479
Ipo4	2.45508E-08	down	0.12841	20.33504
Arl3	2.56635E-08	down	0.04138	30.77396
Olfm3	4.6216E-08	down	0	6.50487
Hspa2	4.72439E-08	down	0	11.05517
Hpca	7.68633E-08	down	17.48788	240.40057
Sbno2	7.79433E-08	down	0	3.31753
Kcnc3	8.79406E-08	down	0	4.67054
Hbp1	1.06193E-07	down	0	9.02724
Ddx17	1.09063E-07	down	0.22003	37.70926
Setd5	1.99631E-07	down	0.19538	13.12633
Gstm5	2.09323E-07	down	0	22.48179
Sirpa	2.19207E-07	down	0.4916	27.18816
Tug1	2.21239E-07	down	0	4.22473
Trip12	3.3015E-07	down	0	2.91075
Grm4	3.33264E-07	down	0	9.15086
Tmem229a	3.60011E-07	down	0.12733	13.28578
Rrn3	4.48785E-07	down	0	5.67468
Klhl12	4.9366E-07	down	0	5.90596
LOC100912481	5.25324E-07	down	0	8.65532
Zmym6	5.75471E-07	down	0	4.52749
Srrm1	6.61882E-07	down	0	5.051
Bicral	8.65223E-07	down	0	4.41703
AABR0700291	9.08758E-07	down	0	5.45223
Ebag9	1.06951E-06	down	0	8.78823
Wnk1	1.08554E-06	down	0	1.60502
Sirpa	1.24794E-06	down	0	6.55716
Tmcc3	1.30467E-06	down	0.03888	7.31506
AABR0700764	1.34531E-06	down	0	6.37209
Pigg	1.34531E-06	down	0	3.08608
Fibcd1	1.60506E-06	down	0	3.87641
Snap91	1.60506E-06	down	0	3.75874
Sfmbt1	1.66311E-06	down	0	5.11488
Sipa1l1	2.12201E-06	down	0.1699	14.85861
Rbfox1	2.17058E-06	down	0	3.58258
Mfsd13a	2.30961E-06	down	0	7.932
Reln	2.35038E-06	down	0.01646	3.18227
Ube2a	2.41669E-06	down	0.01642	12.11393
Kmt5b	2.48938E-06	down	0.03915	6.28551
Hnrnpf	3.31717E-06	down	0	6.39647
Adgrl4	3.37049E-06	down	0	3.24109
Map4k3	3.40655E-06	down	0	3.25978
Ubtd2	3.51723E-06	down	0	5.06763
Asb15	3.69054E-06	down	0	3.16963
Pitpna	4.3613E-06	down	0	7.92001
Cldn5	5.10873E-06	down	0	8.81685
Pla2g6	5.47369E-06	down	0.01356	6.45176
Rgs6	5.48772E-06	down	0	2.15742
Pot1	5.64149E-06	down	0	5.21409
Akap13	5.79993E-06	down	0	1.00036
Gpr155	5.82195E-06	down	0.00292	3.22174
AABR0703547	5.8322E-06	down	0	4.86232
Gtf2e2	6.23491E-06	down	0	8.00432
LOC10834924	6.66797E-06	down	0	2.80842
Asphd2	6.78126E-06	down	0	6.31317
Gars	7.13391E-06	down	0	4.94151
Eif3k	7.25585E-06	down	0	13.24815
Trak1	8.86831E-06	down	0.00586	3.46172
Kdm5b	9.71845E-06	down	0	1.84454
Dhrs7b	1.14066E-05	down	0.01082	10.45477
Nsmce1	1.2294E-05	down	0	7.17271
Nsmaf	1.47206E-05	down	0	3.56029
Ccnk	1.53603E-05	down	0	4.25589
Gabra5	1.60311E-05	down	0	4.40912
Senp5	1.62287E-05	down	0	4.30047
Ankib1	1.72582E-05	down	0	1.61209
Tcte1	1.8661E-05	down	0.21465	10.79393
Ldlrad4	1.97897E-05	down	0	2.18644
Med6	2.05503E-05	down	0	8.41869
LOC689064	2.14904E-05	down	39.70266	399.46271
Lipa	2.20835E-05	down	0.00877	4.32337
Synj2	2.36338E-05	down	0	1.85002
Filip1	2.44052E-05	down	0	1.71255
Lrrc23	2.65471E-05	down	0	5.06065
Enpp6	2.87146E-05	down	0	5.35066
Slc25a27	2.92873E-05	down	0	2.13826
Jam2	3.17115E-05	down	0	2.08768
AABR0705898	3.39071E-05	down	0	2.62694
Irak2	3.50695E-05	down	0	2.70514
Pgm2l1	3.62777E-05	down	0	1.91927
Kbtbd11	3.84743E-05	down	0.26693	11.99227
Bid	3.85743E-05	down	0	3.89692
RGD1306746	3.88396E-05	down	0	3.12054
Tmem216	3.93765E-05	down	0	5.89278
Slc4a3	4.09374E-05	down	0.04243	5.19832
Dyrk1a	4.13239E-05	down	0	2.68461
Vps37c	4.17319E-05	down	0.00532	3.84323
Tm9sf4	4.19005E-05	down	0	3.01676
Kcnma1	4.23304E-05	down	0.0073	3.12263
Klc4	4.82801E-05	down	0.00579	4.03101
Cetn3	4.92427E-05	down	0	7.75591
Ddx17	5.09347E-05	down	0.06086	18.84162
Tmem150c	5.10294E-05	down	0	2.41115
Zfp692	5.13957E-05	down	0	3.91127
LOC10255264	5.1765E-05	down	0	4.55401
Adcyap1r1	5.60376E-05	down	0	1.12336
Gmppa	5.85402E-05	down	0	4.2239
Hsp90aa1	5.89698E-05	down	0	3.13358
C2cd2	5.94032E-05	down	0	1.83425
Prmt6	6.30066E-05	down	0	2.04431
Syt6	6.30066E-05	down	0	1.67635
Vma21	6.44232E-05	down	0	4.42608
AABR0706248	6.78761E-05	down	0	4.15227
AABR0702890	6.83868E-05	down	0	1.06949
Srgn	7.96149E-05	down	0	5.13821
LOC10369378	8.47052E-05	down	0	1.91848
Vegfb	8.5416E-05	down	0.12576	12.51601
Cep57l1	8.87765E-05	down	0	2.62827
Ntrk3	8.94773E-05	down	0	2.42441
Sec31a	9.11646E-05	down	0.00317	1.87207
Rest	9.92094E-05	down	0	1.82179
C3	0.000100008	down	0	1.16626
Taf6	0.000100813	down	0	2.81599
Adora1	0.000102447	down	0	3.90228
LOC10091253	0.000103275	down	0	3.27773
LOC10091259	0.000110185	down	0	10.68109
Grid2ip	0.000111995	down	0	1.6542
Usp34	0.000125437	down	1.31596	15.05683
RT1-DMa	0.000132129	down	0	4.94479
Hipk4	0.000132207	down	0.07025	7.57731
Pld5	0.000134365	down	0	3.13415
Pdhb	0.000134412	down	4.51162	61.87176
Pitpnb	0.000141345	down	0	6.5006
Caap1	0.000144994	down	0	3.47974
Ddx5	0.000146868	down	8.45812	77.02868
Dpyd	0.000153949	down	0	1.74585
Disp1	0.000155281	down	0	1.02293
Uchl3	0.000159358	down	0	5.58341
Dtx2	0.000163564	down	0	1.9828
Taf15	0.000167901	down	0	3.35809
Vps37c	0.000175438	down	0	4.65641
Arid1b	0.000178565	down	0	1.32089
Zfp800	0.000200598	down	0	1.72546
Kcnip2	0.00020427	down	0	5.78195
Cep295	0.000205915	down	0.04669	3.14161
Rnh1	0.000206137	down	0	2.84366
AABR0702898	0.000206137	down	0	2.46391
Donson	0.00023677	down	0	2.02112
Rgs12	0.000239978	down	0.003	1.33817
Tcf25	0.000248491	down	0.90161	20.15225
Zfp449	0.000267899	down	0	1.62083
Mical2	0.000275773	down	0.15079	8.36318
Gtf2ird1	0.000281194	down	0	1.48728
Uaca	0.00028202	down	0.00616	1.60287
Acsl4	0.000284805	down	0.01418	2.09924
P4ha2	0.00029505	down	0.02431	4.05745
Jak3	0.000295311	down	0	1.10638
Steap2	0.000298238	down	0	1.18589
Mboat7	0.00031976	down	0	2.83058
Inpp1	0.00031976	down	0	1.90401
Sorbs1	0.000326255	down	0	1.03184
Tec	0.000396467	down	0.01073	2.51678
Uchl3	0.000420108	down	0.22282	19.43674
Ube3c	0.000425763	down	0.58714	14.13845
LOC10090973	0.000432274	down	0	1.0691
LOC10369216	0.000436974	down	0	3.99314
Gxylt1	0.000445069	down	0.10239	4.08277
Snrpa1	0.000450552	down	0.0252	5.67786
Fgfr1op	0.000487723	down	0	1.07747
Ckap5	0.000500451	down	0.04969	3.05825
Dmac2	0.00050839	down	0.82863	20.44345
Kiss1r	0.000510114	down	0	2.84851
AABR0703119	0.000515916	down	0	1.25537
Nipsnap2	0.000558999	down	0	3.08042
Mxd1	0.000561588	down	0.0062	1.3005
AABR0704160	0.000565527	down	0	3.58987
Art3	0.000565527	down	0	1.65644
Zgpat	0.000571773	down	0.40535	14.27335
Suco	0.000617878	down	0.04408	2.72311
Hus1	0.000619672	down	0.00314	1.0229
Atrip	0.000621473	down	0	1.18934
Map2k5	0.000628963	down	0	1.3891
NEWGENE_15	0.000636572	down	0	1.16658
Map2	0.0006443	down	0	4.62752
Kcnh6	0.000652152	down	0	1.1054
Nrxn2	0.000655685	down	1.67071	16.60638
Hgs	0.000666548	down	0.25024	8.27147
Eng	0.000776036	down	0.00923	1.7396
LOC10091058	0.00078658	down	0	2.41602
Ankrd13d	0.000817146	down	0.78949	16.78299
Asph	0.000817758	down	0	1.61917
Dctn1	0.000839041	down	0.04566	2.95589
Phf1	0.000864071	down	0.16718	7.43019
Ttc7a	0.000889691	down	0.08772	4.00366
Lpxn	0.000904181	down	0.01621	2.89664
AABR0702629	0.000909954	down	0.1236	6.76132
Dnajc7	0.000910484	down	0.01878	2.62885
Tmem2	0.000912064	down	0.11754	3.91517
Pecr	0.000947248	down	0	2.21893
Chodl	0.000960347	down	0	1.03965
Ccdc51	0.000965589	down	0.02798	3.83978
Cep295	0.00096982	down	0.05468	2.44588
Alg12	0.000973691	down	0	1.2966
Psmb4	0.000980097	down	0.75024	26.65065
Mycbpap	0.001009486	down	0.01224	1.65483
Srgap2	0.001023586	down	0.008	1.3683
Magt1	0.001024636	down	0.01071	1.44046
AABR0705865	0.001029636	down	0	2.69537
Rev3l	0.001112924	down	0.16129	3.43881
Fhl1	0.001282992	down	0	1.03082
Cckbr	0.001302789	down	0	2.12583
Tsen34l1	0.00134366	down	0	1.73274
Ofd1	0.001374145	down	0.05928	3.24578
Slitrk5	0.001397355	down	0.0524	2.62643
Abhd10	0.001430855	down	0	1.85412
Max	0.001453868	down	0	4.67272
Nrxn3	0.001477394	down	0	1.73438
Nsmf	0.001501451	down	0	1.39348
Katnal1	0.001526053	down	0	1.50906
Senp8	0.001603297	down	0	2.21046
Recql	0.001630251	down	0	1.19264
Abhd10	0.001630251	down	0	2.41196
Cdr2l	0.001644225	down	0.35331	8.27095
Akr1b8	0.001657839	down	0	1.54651
LOC10834817	0.001657839	down	0	2.09581
Relb	0.001657839	down	0	1.28412
Ddx17	0.001686081	down	0	2.83295
Syt7	0.001714997	down	0	1.352
Chpf	0.001749155	down	5.76049	41.80518
Spata2L	0.001755162	down	1.36718	17.80658
Pitpnb	0.00176701	down	0.00845	1.85097
Ube2j2	0.001778299	down	0.06662	4.79502
Man1c1	0.001785546	down	0.2736	6.47511
Nt5c3a	0.001789211	down	0.06006	4.31435
Slc10a3	0.001903924	down	0	1.09268
Hyal2	0.001973378	down	0	1.05278
Abi1	0.001973378	down	0	1.12068
Dact2	0.0020074	down	0.82273	13.83821
Hrh3	0.002014708	down	0.00484	1.00523
Lsm14a	0.002019603	down	0.05061	2.96419
Nudt5	0.002046443	down	0	2.98396
Rpl19	0.00215624	down	4.89229	66.84619
Gnb3	0.002204444	down	0	1.15245
Anks3	0.002210139	down	0.19821	5.05804
Supt5h	0.00231368	down	2.04408	22.99144
Zfp385a	0.002322045	down	1.68235	21.5099
LOC680910	0.002427282	down	0	1.59173
Pigc	0.002444475	down	0.04306	5.29152
Tmem132e	0.002528944	down	0.01673	1.25047
LOC10369001	0.002553337	down	0.14058	6.59125
Vps25	0.002575215	down	0.29758	11.26176
Unc79	0.002702944	down	0.59093	6.90121
Ncoa3	0.00272644	down	0.01522	1.10675
Macf1	0.002796323	down	0.04837	1.03773
Camk2g	0.002796796	down	0	1.0066
Zfp426	0.002855991	down	0	1.03793
Efemp2	0.002916968	down	0	1.17977
Ahdc1	0.002961975	down	0.91908	9.60324
Klhl22	0.002976595	down	0.00568	1.00645
Mfhas1	0.003262151	down	0.01326	1.15835
Spp1	0.003399988	down	0	1.12119
Dtx3	0.003483367	down	6.42951	47.2141
Lamp1	0.003492979	down	35.58775	139.08367
AABR0705584	0.003504773	down	0.08639	6.34725
Mov10	0.003568355	down	0.09804	3.20095
Usp40	0.003620554	down	0.0202	1.21375
Nrxn1	0.003795697	down	0.47993	9.43727
Pip4k2c	0.003845778	down	1.62418	17.98517
Tmem72	0.003896298	down	0.03854	1.48982
Hnrnpf	0.004020915	down	0.01926	1.54467
Mipep	0.004026506	down	0.44974	9.1496
Cyp1b1	0.004308612	down	0	1.03697
Kbtbd4	0.004403588	down	0.66135	12.54513
Postn	0.004462873	down	0.01326	1.01808
Shc1	0.004496307	down	0.07678	2.76128
Nup43	0.004530361	down	0	1.18024
Letmd1	0.004580097	down	0.1259	5.1678
LOC687399	0.00464724	down	0	1.19794
Eif2ak1	0.004893933	down	0	2.31629
Etv4	0.004957608	down	0.05748	2.57683
Asgr1	0.005299419	down	0	1.12232
RGD1563986	0.005485984	down	0.32127	8.08782
LOC10835113	0.00567658	down	1.00871	19.10969
Pyroxd2	0.006031895	down	0.08555	3.17585
Anapc11	0.006196468	down	0.03415	1.47293
Senp1	0.006262181	down	0	1.32187
AABR0703930	0.006385109	down	0.31428	15.07261
Prune2	0.006460283	down	0.01453	1.86016
LOC10369490	0.006464246	down	0.03334	1.49265
Slco1a4	0.006634708	down	0.57247	8.61499
Sesn3	0.006767254	down	2.83065	21.74259
Gpr12	0.006788248	down	0.02726	1.74923
Bdnf	0.007238531	down	0.04058	1.40602
Ece1	0.007512754	down	0.14321	2.98091
Tbc1d13	0.007515146	down	1.00651	11.98669
Elp5	0.007626927	down	0.72901	12.10839
Tmed4	0.007697738	down	0.06413	2.32474
Camk2g	0.007926281	down	0.13246	2.51901
Cdv3	0.007962431	down	0	1.01682
Elovl1	0.007962431	down	0	1.8639
Cabin1	0.008193325	down	0.09472	1.87461
Ntf3	0.008200813	down	0.03076	1.8166
Adam9	0.008229528	down	1.21556	12.2852
LOC10091151	0.008416168	down	4.82296	51.17524
Naa16	0.008615084	down	0.04975	1.56373
Prpf39	0.008797133	down	0.0532	1.90057
Ubl4a	0.008943735	down	2.4971	20.82047
Cfh	0.00921577	down	0.02215	0.97983
Ddx39a	0.009309032	down	0.06492	2.36819
Fbxo11	0.009813849	down	2.42565	16.77826
Kcnip1	0.009818756	down	0.04912	2.11254
Plekhf1	0.009872993	down	0.25644	5.9038
Ntrk2	0.009940422	down	0.30376	3.97462
Bri3	0.010007755	down	0	1.43556
Tfrc	0.010105207	down	0.96479	8.58855
Fam83h	0.01043207	down	0.09706	2.0656
Eps15l1	0.010479191	down	0.82447	9.09852
AABR0701588	0.010721761	down	0	1.26474
Tab2	0.010735271	down	0.07366	1.71284
Nrxn3	0.011035232	down	0.05798	1.25101
AABR0704441	0.011936406	down	0	1.1083
LOC10834808	0.01225113	down	0.42839	5.69022
Pop1	0.01227387	down	0.05833	1.5489
Dhx16	0.01230462	down	0.30201	5.00347
Fastkd3	0.012355647	down	0.02739	1.17106
Smpdl3b	0.012444442	down	0.29662	5.02347
Orc5	0.012581816	down	0.1101	2.65583
Hba-a2	0.012674551	down	373.97338	998.82511
Aldh1l1	0.012681742	down	0.08761	2.14785
Rgma	0.012703007	down	0.42319	6.62226
Paxip1	0.013039491	down	0.03168	0.99755
Mapt	0.013318177	down	1.77315	11.42125
LOC10035968	0.013595781	down	0.23615	5.52173
AABR0704308	0.013858454	down	0	2.41475
Srpk1	0.013858454	down	0	1.17951
Mrpl14	0.013858454	down	0	1.13303
Uxs1	0.014764157	down	1.14836	12.20155
Rffl	0.014897356	down	0.16814	2.89774
Lingo1	0.015176843	down	0.28544	4.57741
Ncbp2	0.015185511	down	0.29007	5.08881
Drap1	0.01533423	down	2.78561	24.01678
Dock6	0.015971088	down	0.15903	2.16782
Trappc5	0.016132466	down	2.79123	25.2106
Arhgef37	0.016498764	down	0.08475	1.60726
Hira	0.016598976	down	1.08391	8.43317
LOC10091151	0.016719657	down	3.84346	37.69571
Mrgbp	0.016858369	down	0	1.37909
Rassf2	0.016889185	down	0.91086	8.37364
Tshz2	0.016946467	down	0.52401	6.11915
Trio	0.01695465	down	0.06895	1.09988
Dnm1	0.01699703	down	13.23485	49.97879
Grm3	0.017323713	down	0.04912	1.25746
Exoc1	0.017441877	down	1.50681	12.05337
Ass1	0.017447473	down	0.48437	7.21062
LOC10091185	0.01764594	down	0.13825	2.64855
Mindy2	0.017835728	down	1.02557	10.52896
AABR0701808	0.01828133	down	0	1.17391
Aplp2	0.019448467	down	16.0555	55.14768
Rab3c	0.019851216	down	0	1.161
AABR0706048	0.020424483	down	0.15656	3.50031
Rock1	0.02065299	down	1.65222	9.53633
Chchd4	0.020777574	down	0.23396	6.2293
Vps26a	0.021391952	down	0.02585	1.24427
Sgcb	0.021413894	down	0.24781	4.86641
Spred1	0.021417318	down	0.17172	3.20917
Tmem18	0.021779652	down	1.73319	12.43866
Cbln3	0.022020803	down	0.08621	1.86129
Ndrg2	0.022313472	down	1.92252	15.15564
Setx	0.023623732	down	0.26515	2.41424
Kdm3a	0.024617755	down	0.08167	1.32849
Bcap29	0.024830018	down	0.59718	7.12063
Lamp1	0.025079705	down	0.16292	4.19859
Cd52	0.025582611	down	0	1.33155
Zmynd8	0.025629934	down	0.16141	1.99953
Rps6ka2	0.026475226	down	0.80648	5.81832
AABR0703946	0.026499899	down	0.15795	2.54752
LOC691918	0.026703383	down	0	1.55409
Taf12	0.027259955	down	0.6094	7.59997
Tmem19	0.027299816	down	0.88847	8.88468
AABR0702996	0.027422508	down	0.15196	2.28588
Rpl11	0.027529765	down	0.48086	7.0844
Hnrnpc	0.028201873	down	0.11797	2.11804
Drosha	0.028725026	down	4.52259	19.17028
Adamts10	0.02879432	down	0.10333	1.87438
Prss22	0.029064354	down	0.03313	1.09709
Parp8	0.029538668	down	0.04656	0.99531
Naf1	0.02983269	down	0.85148	5.79987
Snx10	0.029841161	down	12.93175	56.30671
Ocln	0.031599838	down	0.2871	3.08124
Mrrf	0.031675731	down	0.31029	3.30222
Zfp467	0.031805733	down	0.20447	2.9717
Abce1	0.031835831	down	4.59792	20.53793
Angel1	0.032019123	down	0.34817	3.50469
Tcp1	0.032177331	down	0.13295	2.1623
Krit1	0.032257412	down	1.17098	8.56381
Spock1	0.032666493	down	0.28896	3.95178
Plekhh1	0.032725908	down	1.82402	10.11485
March8	0.032775403	down	0.10752	1.45542
Fermt2	0.033080907	down	0.19754	2.40981
Yeats2	0.033363992	down	0.78653	5.25535
Scyl2	0.033421163	down	0.33922	3.23347
Rce1	0.033475418	down	0.43682	5.36786
Cdc25b	0.033768379	down	0.12543	1.79306
Polr3e	0.034138273	down	1.03639	7.7924
Snx11	0.034159794	down	0.28237	3.34007
AC094212.1	0.034626949	down	0.13696	2.51042
Ccdc153	0.035031306	down	0.06338	1.38453
Stard8	0.035395723	down	0.32619	3.01228
Rara	0.035498236	down	0.1581	2.23286
AABR0702631	0.036398021	down	1.00892	11.09766
Traip	0.036904661	down	0.27475	3.25849
Msi2	0.037093362	down	0.02853	1.06646
Acad9	0.037415316	down	2.68822	15.18631
Eif2b1	0.037923068	down	0.07706	1.61771
L3mbtl2	0.040126758	down	1.13482	7.5185
Marc2	0.040332193	down	1.40957	10.47428
Mpv17l	0.040332502	down	0.1236	2.36876
Tpp1	0.040470394	down	1.05142	7.93261
Kcnh1	0.040547305	down	0.58432	4.57164
Extl2	0.0407501	down	0.10798	1.72319
Rpp25l	0.041034549	down	0.60954	7.39572
Park7	0.041476077	down	0.05327	1.28205
Lasp1	0.041569497	down	7.25364	26.61424
Aar2	0.043035625	down	2.49066	12.39514
Rcbtb1	0.043046085	down	0.09387	1.49433
Sorcs3	0.043292288	down	0.23116	2.16258
Wdr41	0.044092935	down	0.22858	2.46288
Crem	0.044107749	down	0	1.40711
Usp54	0.045339762	down	0.08563	0.97706
Eif2b4	0.045397911	down	1.48752	11.12809
LOC10013487	0.045938813	down	11.24383	59.37024
Ablim2	0.046781943	down	0.79912	6.50571
Ppm1g	0.047119126	down	4.16851	20.17995
Psmd4	0.047284325	down	0.51992	5.26275
Kalrn	0.047488863	down	0.95252	4.68553
Ddt	0.048474153	down	0.09783	2.17884
Nf1	0.049088201	down	1.48146	6.30082
Grin2a	0.04921969	down	0.36916	3.004
Yap1	0.049389536	down	0.56424	5.44927
Nampt	0.049822762	down	1.63282	9.92581
Chn1	2.39194E-20	up	152.67535	0.7209
Agrn	5.13528E-20	up	20.89284	0
Gabbr1	1.46977E-19	up	33.2655	0
Dkk3	1.7944E-18	up	28.46805	0.00287
Eif4g1	3.61265E-17	up	23.97005	0
Pdhb	4.54738E-16	up	63.91907	0
Tpi1	1.5276E-15	up	160.94964	0.60727
Fscn1	6.4266E-14	up	52.73437	0
Ssx2ip	1.00671E-13	up	22.82997	0
Ddr1	1.11014E-13	up	22.76765	0.00391
Elovl2	4.40376E-13	up	18.32345	0
Chd6	5.60237E-13	up	6.53018	0
Fermt2	5.98273E-13	up	31.10013	0.03133
Slc2a3	1.0923E-12	up	17.25559	0
Zmat2	1.1329E-12	up	33.59323	0
AABR0700039	7.68625E-12	up	109.95114	0.39091
Ppm1f	1.1406E-11	up	11.99307	0
Camk2n1	3.84783E-11	up	183.89977	0.21088
Chd8	3.97216E-11	up	6.03181	0
Plxna3	1.14766E-10	up	6.40475	0
LOC10091158	2.01925E-10	up	12.61996	0
Sgcb	3.93306E-10	up	21.22076	0.05723
LOC10369001	4.6664E-10	up	11.24458	0
LOC10368994	5.07546E-10	up	7.58039	0.00754
Megf9	7.39495E-10	up	17.98484	0.02201
Slit2	1.40103E-09	up	5.34561	0
Calcoco1	2.51389E-09	up	11.80761	0
Arnt2	2.88454E-09	up	22.42225	0.2912
Eif4g1	2.99985E-09	up	7.43165	0
Myo9a	3.92447E-09	up	4.39074	0
Wbp4	4.37095E-09	up	10.97737	0
Nploc4	5.5163E-09	up	18.5249	0.10537
Cep295	7.70084E-09	up	4.22329	0
Zcchc12	1.42272E-08	up	14.49484	0
Smarcb1	1.83463E-08	up	7.92821	0
Ampd2	1.85826E-08	up	11.98356	0.01283
Tmem9	2.01456E-08	up	20.10257	0
Plat	2.35881E-08	up	11.25461	0
Ubl7	4.03593E-08	up	19.71695	0
Slain2	5.37438E-08	up	5.41181	0
Il17ra	5.99359E-08	up	6.65202	0
Porcn	8.07654E-08	up	13.13898	0
Fmod	9.42619E-08	up	6.80325	0
Slc1a3	9.91866E-08	up	176.69699	19.8956
Snx6	1.18221E-07	up	12.10713	0
Zbtb22	1.20886E-07	up	9.08013	0
Gpatch11	1.58876E-07	up	5.61936	0
Fbxw11	1.77035E-07	up	5.50355	0
Morc3	1.82966E-07	up	6.37903	0.00349
AABR0701506	1.95569E-07	up	47.90223	0
Dhrs7b	1.96457E-07	up	17.99581	0
Ascc3	1.98169E-07	up	3.49345	0.00193
Adcy2	2.53639E-07	up	34.86999	0.74625
Nup214	2.65444E-07	up	3.73391	0
Tle4	5.23459E-07	up	4.71087	0
LOC10091151	5.29593E-07	up	87.63045	0.37338
Samp	5.33441E-07	up	16.93399	0
Reck	5.4879E-07	up	4.44478	0
Grm4	6.03373E-07	up	7.58508	0
Col6a1	6.86221E-07	up	5.86508	0
Paics	7.02828E-07	up	9.15608	0
Arhgap39	7.23313E-07	up	3.88081	0
Foxj2	8.23548E-07	up	3.99984	0
LOC10091188	8.81162E-07	up	11.2999	0
Pomk	1.09016E-06	up	7.00214	0.00492
LOC10369399	1.124E-06	up	5.5906	0
Ywhah	1.19151E-06	up	75.82956	1.74317
Dock6	1.24616E-06	up	2.4729	0
Dctn1	1.31021E-06	up	87.61118	8.50664
Vav2	1.63737E-06	up	3.012	0
Mff	1.69579E-06	up	7.15211	0
Zfp329	1.74762E-06	up	1.76969	0
LOC10091222	1.83777E-06	up	25.09244	0
Ywhag	1.92986E-06	up	67.01254	2.03441
Dcx	2.21668E-06	up	1.66667	0
LOC10091161	2.48067E-06	up	6.38708	0
RGD1564541	2.48067E-06	up	3.98751	0
Arhgef37	2.54521E-06	up	2.3624	0
Tmem186	2.57153E-06	up	7.00177	0
Vrk3	3.16371E-06	up	7.96239	0
Sec24c	3.25714E-06	up	3.83711	0.00323
Fam160a2	3.49465E-06	up	2.06187	0
Acvr1	3.51109E-06	up	5.59097	0.00476
Rbm39	3.56894E-06	up	6.78817	0
Vps29	3.98778E-06	up	13.42068	0
Rmnd5b	4.38892E-06	up	6.05491	0
Sidt1	4.55646E-06	up	2.20554	0
Me3	5.12949E-06	up	6.67326	0
Ndufaf1	5.18531E-06	up	6.52548	0
Kif1b	5.45881E-06	up	80.97998	14.38412
Cit	5.53435E-06	up	1.95026	0
Ube2h	6.7607E-06	up	33.66987	1.79607
AABR0702413	7.20766E-06	up	2.5591	0
Mmadhc	7.32912E-06	up	8.23463	0
LOC10834808	7.75038E-06	up	10.2818	0
Mto1	8.77316E-06	up	5.57988	0
Angel1	8.77316E-06	up	2.53284	0
Znf740	1.10326E-05	up	2.88439	0
Ddx17	1.2401E-05	up	35.84739	1.2112
Ppig	1.2908E-05	up	11.60439	0.1053
Dnm2	1.38662E-05	up	3.38354	0
LOC10091176	1.48046E-05	up	2.74493	0
Chd4	1.54085E-05	up	19.65816	1.10478
Dcun1d2	1.61998E-05	up	3.57196	0
Sh3bp1	1.66968E-05	up	3.72122	0
Zfp667	1.68547E-05	up	3.67362	0.00426
Slc4a5	1.69429E-05	up	3.67273	0.00426
Carmil1	1.73158E-05	up	1.89247	0
Tor1aip2	1.81813E-05	up	1.84353	0
Rnf216	1.89784E-05	up	1.7554	0
Alk	1.90954E-05	up	1.93656	0
RGD1307100	2.09866E-05	up	1.90651	0.01504
Bdnf	2.14795E-05	up	2.31672	0
Erc1	2.18975E-05	up	4.84383	0.07413
Dync1i2	2.20179E-05	up	4.908	0.01026
Calcoco1	2.2724E-05	up	4.26681	0
Fbxo38	2.67911E-05	up	2.55497	0.00328
Dopey1	2.73852E-05	up	2.68648	0.01494
Cic	2.81321E-05	up	6.79011	0.09532
Rasgrp3	2.99103E-05	up	1.92363	0
Nfil3	3.17215E-05	up	4.14691	0
Phrf1	3.17557E-05	up	2.43599	0.00551
AABR0707139	3.43337E-05	up	7.39042	0
AABR0706641	3.62114E-05	up	14.13906	0
NEWGENE_13	3.8988E-05	up	3.14247	0
Cops7a	3.99485E-05	up	15.11773	0.14744
Mrpl42	4.11609E-05	up	11.29726	0
Daxx	4.2049E-05	up	4.16196	0.00595
Rbbp6	4.22992E-05	up	1.2593	0
Fam83h	4.25894E-05	up	1.66046	0
Ccnt2	4.28521E-05	up	2.61984	0.01017
Rffl	4.49953E-05	up	2.06186	0
Rpgrip1l	4.52963E-05	up	1.39368	0.00203
Yap1	4.69026E-05	up	5.18705	0
Prkab2	5.39696E-05	up	1.42195	0
Magi3	5.63251E-05	up	1.18539	0
Chrna5	5.67292E-05	up	2.43153	0
Jak3	5.88009E-05	up	1.84481	0
LOC10091075	6.46132E-05	up	5.06983	0
Acox1	6.60481E-05	up	1.72555	0
Mff	6.63625E-05	up	4.69647	0.00754
Dzip1l	6.70254E-05	up	1.72678	0
AABR0700602	6.70254E-05	up	6.51413	0
Mfsd14a	6.70254E-05	up	3.56242	0
Focad	7.07264E-05	up	8.83432	0.33054
AABR0706248	7.43616E-05	up	3.98219	0
Proca1	7.83814E-05	up	4.46119	0
Arhgef1	8.07932E-05	up	1.95898	0
Grip2	8.32929E-05	up	2.06206	0
Kcnh1	8.4577E-05	up	1.5361	0
Zbtb9	8.4577E-05	up	3.07603	0
Ahi1	9.64917E-05	up	1.88989	0
Trim65	9.8795E-05	up	1.97056	0
Rhot1	0.000102962	up	11.95189	0.21252
Sstr1	0.000118494	up	5.62809	0.06625
NEWGENE_13	0.000119683	up	1.52846	0
Prrt4	0.000119683	up	1.91031	0
Trim26	0.000124262	up	3.74994	0.00714
Aspa	0.00012519	up	9.43602	0.02973
RGD1311892	0.000126709	up	1.83431	0
Mier1	0.000135335	up	1.17239	0
Prr36	0.000135335	up	1.6963	0
Asic1	0.000139904	up	1.40342	0
Clasp1	0.000139904	up	1.08116	0
Fbxl3	0.000143048	up	7.62763	0.03896
Eif5b	0.00014345	up	1.45112	0
Fgfr3	0.000144412	up	17.81275	0.71719
Amer2	0.000156057	up	2.91156	0
Mtfr1l	0.000157388	up	2.76642	0
Xiap	0.000162849	up	2.26499	0
LOC10369490	0.000167888	up	96.81615	6.41479
LOC10834813	0.000171679	up	5.28714	0.01105
Mx2	0.00017646	up	3.42416	0.01185
Fam81a	0.000178334	up	33.17256	2.8079
Rpl22	0.000180631	up	10.49816	0
Cldn2	0.000188683	up	2.63267	0.00928
Dnal1	0.000193777	up	1.66918	0
Cd99l2	0.000193777	up	1.96646	0
Sorcs2	0.000194898	up	2.6857	0.02516
Tdp1	0.000195499	up	1.84623	0
Tpd52l1	0.000202359	up	12.80819	0.09904
Slc4a8	0.000203329	up	6.22186	0.0951
Trim5	0.000213776	up	1.9003	0
LOC10255002	0.000215715	up	1.51248	0
Acta1	0.000227803	up	3.138	0
Mycbpap	0.000227803	up	1.55385	0
Il11ra1	0.000232014	up	2.61418	0
Vrk1	0.000236319	up	2.37081	0
Pomp	0.000236721	up	27.95614	0.38797
LOC10035968	0.000238508	up	4.7141	0
Ddi2	0.00024983	up	1.23958	0
Agk	0.000254542	up	2.36259	0
RGD1564887	0.000256939	up	2.88822	0
Apbb3	0.000264296	up	2.12273	0
LOC10369003	0.000264296	up	3.31133	0
Zfp384	0.000269344	up	1.60086	0
Gpbp1l1	0.000270974	up	8.23767	0.12711
Nfib	0.000271912	up	1.93582	0
AABR0701505	0.00029464	up	35.53231	0.82452
Nek4	0.000296437	up	1.49404	0
Ppp2r5c	0.000301351	up	5.97269	0.04218
LOC10091157	0.000305212	up	2.3793	0
Alkbh8	0.000330223	up	1.60466	0
AABR0700411	0.000337695	up	2.71665	0.01135
Sema6c	0.000350647	up	1.18232	0
Mdm1	0.000367555	up	1.76787	0.00468
Slc6a4	0.00036887	up	1.15772	0
Dixdc1	0.000400542	up	1.61928	0
Smndc1	0.000417653	up	2.71735	0
Gal3st1	0.000460591	up	3.11087	0.00887
Nae1	0.000464166	up	13.43029	0.30706
AABR0705900	0.000464631	up	2.50749	0
Cacng7	0.000484265	up	6.99866	0.09694
Tubgcp5	0.000486424	up	5.69708	0.11653
Acad9	0.000490799	up	17.48125	0.7535
LOC10369003	0.000496052	up	1.66238	0
Rnmt	0.000518514	up	1.01515	0
AABR0705130	0.000524331	up	1.24017	0
Stard10	0.000546418	up	13.46514	0.23
Paqr7	0.000568285	up	2.61521	0.01288
Adar	0.000578073	up	1.29173	0.00398
AABR0700262	0.00058066	up	2.51343	0
Vps13c	0.000592288	up	4.60421	0.25375
Pcgf6	0.000594224	up	1.52542	0
Abhd2	0.000615349	up	1.98767	0
Nrxn2	0.000633384	up	31.04657	3.24803
Tmem231	0.000635152	up	3.43337	0.03577
Gpm6a	0.00064631	up	39.59379	4.30407
Tdrd7	0.000653616	up	4.28552	0.07632
Casr	0.00065996	up	1.3519	0.00699
Impad1	0.000668579	up	1.78142	0
Agap3	0.000679281	up	29.35984	2.24485
Dock1	0.000692685	up	6.71026	0.34368
Cdkn1c	0.000693233	up	1.60647	0
Fbxo31	0.000701715	up	1.96586	0
Sema3f	0.000707248	up	2.92623	0.05809
Mgst1	0.000727227	up	4.52168	0.01513
Itgb5	0.000736341	up	2.68624	0.03126
Pigc	0.000774743	up	2.06736	0
Ubr2	0.000782973	up	4.42129	0.11109
AABR0706750	0.00078859	up	36.67474	0.74677
Acp5	0.000836233	up	1.99967	0
Trim13	0.000836233	up	2.05106	0
Tmem255a	0.000869421	up	2.06874	0
Fam81a	0.000875157	up	1.83826	0.00497
Plp1	0.000879008	up	426.00816	151.4816
RGD1565222	0.000915559	up	1.30529	0.0049
Msantd2	0.001126728	up	1.49232	0
Hrh2	0.001142978	up	1.36576	0
Ssbp2	0.001211263	up	1.60861	0
Dtx3	0.001216711	up	24.51675	1.28252
Gnb3	0.0012292	up	1.54173	0
Stard6	0.001247501	up	1.8925	0
Bace2	0.001304693	up	1.32523	0
Med7	0.001304693	up	2.00028	0
Clstn1	0.001347319	up	10.99314	0.73189
Cpt1a	0.001380079	up	7.12114	0.31893
Drd1	0.001383673	up	9.77504	0.32145
Lsm6	0.001408291	up	1.07321	0
Ocln	0.001410587	up	2.47705	0.0389
RT1-A1	0.001430358	up	1.44785	0
Thop1	0.001452905	up	1.59748	0
Mtrex	0.001461431	up	2.51862	0.03999
Adcy5	0.001489221	up	44.73311	8.48588
Pc	0.001532649	up	7.43432	0.33305
Clec11a	0.001593082	up	3.47149	0.06601
AC126641.1	0.001699933	up	5.54207	0.08315
Tmem39a	0.001708055	up	1.01479	0
Zkscan3	0.00174927	up	1.19716	0.0056
Polr2e	0.001766451	up	1.85005	0
Ica1	0.001789855	up	1.54239	0.00728
Cttn	0.001944376	up	13.26946	0.84448
Hfe	0.00195933	up	1.41777	0
Car3	0.001976136	up	15.48947	0.6526
Elovl1	0.001984212	up	39.78349	3.52108
Cttn	0.00208283	up	5.29403	0.09396
Tmem63c	0.002214596	up	5.02151	0.23145
Txnrd3	0.002217791	up	1.00451	0.00517
Mical2	0.002240766	up	56.88034	11.95514
Herc1	0.002279491	up	3.54936	0.30596
Hspa1a	0.002330971	up	1.42128	0.00746
Akr1a1	0.002403285	up	256.60319	63.18021
LOC10036595	0.002541797	up	1.05884	0
Relb	0.002541797	up	1.07147	0
Tpd52	0.002592403	up	1.92945	0
Trmt5	0.002637522	up	1.84572	0.01997
Ptprm	0.002654936	up	5.70153	0.31432
Enpp6	0.002694345	up	5.21245	0.1164
Six3	0.002698009	up	1.73736	0
Kiaa0408L	0.002739443	up	2.06917	0.04501
Arhgef9	0.002744069	up	3.97407	0.08051
Dnm2	0.002758504	up	2.68526	0.06221
Arl6ip5	0.002799806	up	56.40021	6.26171
Akt1	0.002946122	up	21.73608	1.98567
Dap3	0.002951553	up	3.75712	0.06397
Phf14	0.003011962	up	1.51234	0.02753
Gatm	0.003019775	up	49.45468	8.08042
LOC10369487	0.003053591	up	3.05954	0
Zfp61	0.003082371	up	1.52922	0.0176
Qtrt2	0.003257186	up	1.3048	0
Fhod3	0.003275299	up	6.89133	0.42146
Igtp	0.003403374	up	1.20415	0
Ctnbp2	0.003414757	up	11.45501	1.31764
Mob2	0.003479947	up	1.33301	0
Hivep2	0.00357709	up	27.12846	6.49222
Sirt4	0.003640529	up	1.09271	0
Clpb	0.003661167	up	12.96309	0.77178
Prpf8	0.003976889	up	33.73979	8.19219
Clip3	0.003983157	up	79.96161	20.41745
Nrg2	0.00401536	up	2.35221	0.0386
Scaper	0.004113597	up	1.42298	0.03547
Ccdc84	0.00439848	up	1.44735	0
Manbal	0.004449707	up	39.37867	3.5175
Cyth1	0.004481132	up	5.20877	0.22708
LOC10091183	0.004505412	up	8.14919	0.67105
Sacs	0.004828129	up	3.43091	0.37376
LOC10091024	0.004862042	up	1.99677	0
Sept7	0.004922129	up	37.2844	5.74429
Smarcad1	0.004969436	up	3.54534	0.16789
LOC10035966	0.004991648	up	3.6112	0.02604
Ash1l	0.005010954	up	11.23031	1.89197
Adam23	0.005108122	up	1.22514	0.01999
Fbxw5	0.005335353	up	19.06223	1.85968
Ppp1cc	0.005369549	up	67.66558	14.29662
Palm3	0.005393607	up	1.68017	0.03363
Ubc	0.005522865	up	164.46117	51.05634
Sec31a	0.005535264	up	3.68125	0.15724
Hspa2	0.005583584	up	8.11494	0.45359
Mark2	0.005942767	up	2.28874	0.07522
Kat6a	0.006114961	up	2.16921	0.12025
Plekho1	0.006171642	up	16.82624	1.05025
Stx7	0.006255496	up	11.97747	0.81631
Myh6	0.006364524	up	2.04001	0.08824
Etnk2	0.006389075	up	2.96325	0.0839
Cc2d1a	0.006540443	up	15.82207	1.8226
Lat2	0.006547345	up	1.88601	0
Cacna2d3	0.006553584	up	1.43978	0.0351
Lsm14a	0.006625655	up	1.28732	0.01046
Faim	0.006738699	up	1.80665	0
Evl	0.006803573	up	6.66735	0.27559
Atxn1	0.006919629	up	7.15421	0.59077
RGD1304624	0.006937819	up	1.08664	0
Hspa4l	0.006943034	up	7.04706	0.19193
Acvrl1	0.007509109	up	2.35871	0.08886
Gpm6a	0.007598845	up	254.04762	97.22868
Metap2	0.007764965	up	15.77949	1.32222
Usp19	0.007946753	up	14.22537	1.95353
Cby1	0.008316932	up	8.48311	0.2164
Pcdha6	0.008458488	up	1.67813	0.07003
Fn1	0.008519813	up	1.12496	0.04771
Pla2g2c	0.008580263	up	1.40609	0.04721
Map2	0.00858513	up	2.0964	0
Ppp2ca	0.008862613	up	1.17737	0
Mapt	0.009008074	up	2.3614	0.07636
Znrd1as1	0.00913761	up	1.59357	0.01484
Timm10b	0.009152347	up	2.19699	0
Caskin2	0.009295267	up	8.95099	1.04891
Dcst2	0.009405089	up	2.19663	0.09388
AABR0706285	0.009454989	up	2.86796	0
Pygo2	0.009691548	up	4.44873	0.24743
Ppp1cc	0.010100765	up	3.037	0.09628
Mtss1l	0.010231471	up	39.73946	9.02984
Gatad2b	0.010507504	up	5.1959	0.33943
Uchl5	0.010880286	up	3.31776	0.12821
Hnrnpul1	0.011657039	up	20.96315	3.74541
Rps8	0.01170095	up	31.66593	2.60243
RGD1304694	0.011828402	up	38.25773	8.5643
LOC10834810	0.011937494	up	3.38711	0.0802
Tceal9	0.012061445	up	31.38778	3.29227
Dnal1	0.01217065	up	6.20811	0.45337
Shank2	0.012271373	up	6.37133	0.91649
Zbtb5	0.012701196	up	3.18053	0.12493
Pbxip1	0.013141389	up	24.65593	4.4005
Pard3	0.013266737	up	1.34484	0.06533
Begain	0.013314632	up	7.75421	0.66814
Epb41l1	0.013500313	up	26.49349	4.50345
Atp2c1	0.013546894	up	1.45919	0.05056
Alms1	0.013611758	up	1.11432	0.06643
Stpg1	0.013774552	up	1.00403	0.02539
Svbp	0.013915183	up	1.19903	0
AABR0703608	0.013917327	up	2.51128	0.0555
Zfp622	0.014224585	up	4.81838	0.23399
Pip4k2c	0.014340635	up	21.03036	3.66837
Cited1	0.014648825	up	1.48462	0.01689
Zc3h4	0.014725116	up	5.67402	0.6596
Washc2c	0.015035849	up	2.12117	0.02441
Nipa2	0.015289621	up	11.03888	1.26664
Asah2	0.016146208	up	2.8121	0.20817
Vps29	0.016922904	up	7.75023	0.44792
Becn1	0.016960904	up	16.94506	2.17077
Stat5b	0.017091894	up	9.1976	0.95154
Cope	0.017555542	up	1.18829	0
Crocc	0.018639594	up	3.02051	0.29733
Tyk2	0.018790336	up	2.3762	0.17624
Ube2c	0.01981474	up	1.0303	0
Casc3	0.020369859	up	3.8641	0.32148
Mapk4	0.020530601	up	6.12512	0.73977
Tbc1d9	0.020928693	up	7.54944	1.17682
Ncstn	0.021453494	up	5.21057	0.44988
AABR0705013	0.021513183	up	1.38078	0
Kif23	0.021629017	up	1.13888	0.05071
Lpgat1	0.022072555	up	20.31593	4.32673
AABR0703065	0.022425288	up	1.72224	0
AC134224.3	0.022677834	up	3.59532	0.14479
Tnfaip8	0.023176297	up	2.08329	0.11298
Atp1a1	0.023729989	up	260.20463	124.90349
Trim46	0.023868933	up	5.46577	0.3521
LOC103689971	0.024384032	up	1.0112	0
Papd5	0.024768888	up	4.29745	0.31993
NEWGENE_13	0.02540597	up	2.96747	0.19426
RGD1561796	0.02543385	up	1.0322	0
Mroh1	0.025504863	up	5.63749	0.77064
Dda1	0.026303624	up	9.79	1.02448
LOC10369511	0.026491411	up	7.11755	0.45898
Ccar1	0.026568928	up	2.35968	0.19759
P2rx6	0.026658515	up	1.36326	0.06917
Fat3	0.026945716	up	1.97401	0.26189
Wdr31	0.027133619	up	2.98617	0.17699
Zscan26	0.027172873	up	2.63081	0.22538
Slc39a7	0.027493902	up	19.20141	3.07095
Smarca4	0.028327136	up	1.18495	0.0852
Slc12a6	0.028703085	up	14.60795	3.60854
Rpe	0.028732259	up	2.72959	0.15762
Tmem53	0.029010846	up	1.71524	0.06163
Gng3	0.029145731	up	88.33034	23.15259
Ankrd17	0.029235534	up	2.62726	0.31737
Sin3b	0.029617883	up	11.8743	1.15993
Appl1	0.029897791	up	15.04703	2.40511
Prodh1	0.030200195	up	11.25862	1.49551
Anxa7	0.030702332	up	8.30644	0.88927
Orc5	0.03148843	up	8.39851	0.96479
Rabl3	0.032208128	up	3.32156	0.25657
Sec31a	0.032667755	up	2.72491	0.27025
Ap3b2	0.032819218	up	31.5436	8.93943
Cyp2j10	0.033313088	up	3.61878	0.26674
Gpr3	0.033611826	up	2.87733	0.16369
Kmt2e	0.034123875	up	7.16341	1.29572
AABR0702888	0.03481106	up	1.02716	0.05113
Cep68	0.03492828	up	3.7061	0.33559
Tle4	0.034945242	up	3.66908	0.53675
Uhrf2	0.03579721	up	10.79234	1.94511
Lrrc56	0.036804761	up	1.62863	0.10803
Wnk2	0.03771474	up	13.55057	3.5238
Pnkp	0.038228419	up	5.64045	0.55256
Tapbp	0.038392281	up	5.85251	0.59099
Csnk1d	0.039256787	up	1.52913	0.09412
Slc7a1	0.039679243	up	7.63613	1.61281
Cbx3	0.040130119	up	49.89705	13.41249
Ttll5	0.040277909	up	2.68885	0.28608
Uap1	0.040539553	up	1.12353	0.07031
Tmem263	0.041099807	up	2.2661	0.14656
Spire1	0.041912243	up	1.92381	0.20724
Rpl10	0.042183236	up	173.09725	55.34022
Setd4	0.042387391	up	1.54508	0.10153
Fbxo31	0.042610104	up	17.64611	3.14265
Nbr1	0.043281285	up	14.85334	3.5783
Lyn	0.044040197	up	4.81708	0.63704
Smim8	0.044077826	up	2.35945	0.13906
Epb41l3	0.044604019	up	3.44407	0.42205
Snrpa1	0.046180116	up	7.55886	0.77405
Rps4x	0.046552068	up	21.90739	3.28708
Ano3	0.046562095	up	12.10301	3.18756
Uhrf1	0.046822577	up	0.93698	0.0632
Med26	0.047265574	up	2.20834	0.23051
Eny2	0.047445947	up	10.15142	1.5372
Zfp280d	0.047771086	up	1.77123	0.18239
Herc4	0.048246207	up	3.32823	0.41619
Eif4e2	0.048296536	up	7.25291	0.74695
Myl1	0.049949829	up	1.93442	0.10904
