# Supplementary material for: Sodium butyrate mediates histone crotonylation and alleviated neonatal rats hypoxic–ischemic brain injury through gut–brain axis
Source: Front Microbiol. 2022 Oct 20;13:993146. doi: 10.3389/fmicb.2022.993146 (PMC9631217; doi:10.3389/fmicb.2022.993146)
Supplement: Supplementary file 1 [file Data_Sheet_1.ZIP › Supplementary Table/Table S7.pdf]

Table S7 ChIP -seq enrichment analysis of promoter regions in HIBD and HIBD+SB group（HIBD+SB vs HIBD Up）

RefSeq_name	GeneSymbol	chr	Fold change	P-value	FDR	HIBD+SB vs HIBD	Length	HIBD_SB	HIBD_IP.cnt	HIBD_SB_IP.avg	HIBD_IP.avg	Peak_classification	Peak_To_TSS
NM_001108406	Sptlc1	chr17	278.3	5.65176E-10	2.75666E-05	Up	960	27.73	0	27.73	0	promoter	541
NM_053871	Srp54a	chr6	242.5	7.20612E-09	0.000135513	Up	800	24.15	0	24.15	0	promoter	-1375
NM_001108022	RGD1304624	chr6	242.5	7.20612E-09	0.000135513	Up	800	24.15	0	24.15	0	promoter	949
NM_199398	Panx3	chr8	233.6	1.36367E-08	0.00019968	Up	1080	23.26	0	23.26	0	promoter	314
NM_001108712	Mbip	chr6	233.6	1.36367E-08	0.00019968	Up	1200	23.26	0	23.26	0	promoter	1665
NM_053584	Gosr1	chr10	215.7	4.89345E-08	0.000422358	Up	620	21.47	0	21.47	0	promoter	1994
NR_032290	Mir674	chr3	215.7	4.89345E-08	0.000422358	Up	680	21.47	0	21.47	0	promoter	-1179
NM_053447	Hdac2	chr20	215.7	4.89345E-08	0.000422358	Up	620	21.47	0	21.47	0	promoter	-1033
NM_001000408	Olr833	chr5	206.7	9.28024E-08	0.000581966	Up	1120	20.57	0	20.57	0	promoter	-1280
NM_001037651	Prtg	chr8	206.7	9.28024E-08	0.000581966	Up	760	20.57	0	20.57	0	promoter	-1462
NM_001108018	Cbll1	chr6	28.67213115	2.6148E-10	1.63742E-05	Up	1020	34.88	1.12	34.88	1.12	promoter	-740
NM_001135174	Tomm7	chr4	24.27868852	9.85034E-09	0.000168883	Up	1000	29.52	1.12	29.52	1.12	promoter	1114
NM_022245	Cyb5a	chr18	22.07377049	6.01136E-08	0.000461146	Up	1060	26.83	1.12	26.83	1.12	promoter	-884
NM_001108530	Ube2d1	chr20	22.07377049	6.01136E-08	0.000461146	Up	880	26.83	1.12	26.83	1.12	promoter	-1749
NM_001100575	Mpp7	chr17	15.71367521	1.29438E-09	4.70324E-05	Up	1500	36.67	2.24	36.67	2.24	promoter	-1742
NM_031602	Kcnj10	chr13	15.71367521	1.29438E-09	4.70324E-05	Up	1400	36.67	2.24	36.67	2.24	promoter	-1810
NM_001034032	Dnajc12	chr20	15.33333333	2.31472E-09	6.84249E-05	Up	1480	35.78	2.24	35.78	2.24	promoter	384
NM_001107982	Zmym4	chr5	14.18376068	1.31643E-08	0.00019968	Up	1380	33.09	2.24	33.09	2.24	promoter	1673
NM_001035007	Mbtps2	chrX	14.18376068	1.31643E-08	0.00019968	Up	1180	33.09	2.24	33.09	2.24	promoter	876
NM_138523	Prpf18	chr17	13.41880342	4.17305E-08	0.0003945	Up	1660	31.3	2.24	31.3	2.24	promoter	-197
NM_001105995	Klhl8	chr14	13.41880342	4.17305E-08	0.0003945	Up	1180	31.3	2.24	31.3	2.24	promoter	-1780
NM_001109647	Spata5l1	chr3	13.03846154	7.41737E-08	0.000528165	Up	1300	30.41	2.24	30.41	2.24	promoter	-607
NM_001134736	Dnaj3	chr2	11.17681159	4.02767E-09	9.53424E-05	Up	1720	38.46	3.35	38.46	3.35	promoter	-718
NM_057136	Epn1	chr1	10.86467487	1.34559E-13	8.33241E-08	Up	1580	61.72	5.59	61.72	5.59	promoter	-502
NM_001024342	Dnaj1	chr5	10.65797101	1.23154E-08	0.000195928	Up	1340	36.67	3.35	36.67	3.35	promoter	1881
NM_001177442	Glcci1	chr4	10.4	2.14911E-08	0.000271083	Up	1560	35.78	3.35	35.78	3.35	promoter	-1142
NM_001106584	Rpa3	chr4	10.4	2.14911E-08	0.000271083	Up	1560	35.78	3.35	35.78	3.35	promoter	869
NM_001107373	Rbfa	chr18	10.07732865	2.10076E-12	5.62088E-07	Up	1720	57.24	5.59	57.24	5.59	promoter	799
NM_001105766	Pkmyt1	chr10	8.829321663	9.59975E-09	0.000168179	Up	1680	40.25	4.47	40.25	4.47	promoter	-1216
NM_001107165	Rtbdn	chr19	8.63238512	1.6474E-08	0.000229431	Up	1340	39.35	4.47	39.35	4.47	promoter	1761
NM_001107793	Acss2	chr3	7.456494325	2.6618E-11	3.40619E-06	Up	1240	59.03	7.83	59.03	7.83	promoter	740
NM_017153	Rps3a	chr2	7.405975395	1.91696E-08	0.000252627	Up	1000	42.04	5.59	42.04	5.59	promoter	901
NM_001109075	Cfap126	chr13	7.247803163	3.23271E-08	0.000330595	Up	1220	41.14	5.59	41.14	5.59	promoter	-748
NM_001108491	Me3	chr1	6.933216169	9.14019E-08	0.000581966	Up	1020	39.35	5.59	39.35	5.59	promoter	1202
NM_001134508	RGD1307603	chr1	6.450807636	3.37218E-08	0.000337203	Up	1100	43.83	6.71	43.83	6.71	promoter	981
NM_001047093	Klhl5	chr14	5.848451327	4.30867E-09	9.98527E-05	Up	1840	52.77	8.94	52.77	8.94	promoter	957
NM_001304352	Zbtb4	chr10	5.765447667	5.39268E-08	0.000452386	Up	1020	45.62	7.83	45.62	7.83	promoter	1393
NM_001011922	Nedd9	chr17	5.765447667	5.39268E-08	0.000452386	Up	1320	45.62	7.83	45.62	7.83	promoter	-1860
NM_001107489	Zfp575	chr1	5.75	7.04844E-09	0.000135513	Up	1160	51.88	8.94	51.88	8.94	promoter	1496
NM_001012047	Btd	chr16	5.718085106	1.22512E-10	9.65834E-06	Up	2020	64.4	11.18	64.4	11.18	promoter	-294
NM_001109437	Dbr1	chr8	5.490322581	6.94971E-11	6.49891E-06	Up	2220	67.98	12.3	67.98	12.3	promoter	355
NM_001109163	Fam111a	chr1	5.148784825	6.12732E-13	2.00377E-07	Up	2280	86.76	16.77	86.76	16.77	promoter	1250
NM_001013100	Abcd4	chr6	5.129032258	7.57064E-10	3.26619E-05	Up	2100	63.5	12.3	63.5	12.3	promoter	787
NM_001007620	Pdhb	chr15	5.116141732	2.71841E-08	0.000300406	Up	1740	51.88	10.06	51.88	10.06	promoter	-1797
NM_001008764	Psenen	chr1	5.083333333	5.73575E-09	0.000121449	Up	1000	57.24	11.18	57.24	11.18	promoter	365
NM_001008775	U2af1l4	chr1	5.083333333	5.73575E-09	0.000121449	Up	1000	57.24	11.18	57.24	11.18	promoter	-488
NM_001013203	Trim44	chr3	5.004432624	9.19783E-09	0.000163079	Up	1800	56.35	11.18	56.35	11.18	promoter	1320
NM_198732	Commd3	chr17	4.985483871	1.94349E-09	6.56818E-05	Up	1960	61.72	12.3	61.72	12.3	promoter	1689
NM_001163062	Zfp870	chr7	4.92464539	1.47191E-08	0.000214816	Up	1740	55.45	11.18	55.45	11.18	promoter	359
NM_053425	Ccs	chr1	4.687056738	5.95404E-08	0.000461146	Up	2240	52.77	11.18	52.77	11.18	promoter	1137
NM_001271283	Golm2	chr3	4.608156028	9.44374E-08	0.000589706	Up	2480	51.88	11.18	51.88	11.18	promoter	-1863
NM_001009678	Nepro	chr11	4.531100478	2.20082E-09	6.84249E-05	Up	2060	66.19	14.53	66.19	14.53	promoter	-646
NM_001106736	Fkbp3	chr6	4.469583049	3.46254E-09	9.07208E-05	Up	1880	65.29	14.53	65.29	14.53	promoter	234
NM_173133	Phax	chr18	4.37352071	2.57321E-08	0.000287524	Up	1560	59.03	13.42	59.03	13.42	promoter	1998
NM_001109346	Cpa4	chr4	4.16404648	3.23569E-08	0.000330595	Up	2020	60.82	14.53	60.82	14.53	promoter	261
NM_001100517	Aars	chr19	4.13229572	1.16851E-09	4.37181E-05	Up	1600	74.24	17.89	74.24	17.89	promoter	293
NM_001109163	Fam111a	chr1	4.088322466	5.46831E-09	0.000117477	Up	1980	68.87	16.77	68.87	16.77	promoter	-1420
NM_001106410	Gpbp1	chr2	3.929460581	2.00892E-08	0.000259412	Up	2440	66.19	16.77	66.19	16.77	promoter	-442
NM_001014099	Paqr8	chr9	3.833796554	1.55967E-08	0.000224288	Up	1660	68.87	17.89	68.87	17.89	promoter	-343
NM_021863	Hspa2	chr6	3.796441654	7.87489E-09	0.000142484	Up	2600	72.45	19.01	72.45	19.01	promoter	-639
NM_053684	Hcn2	chr7	3.770005928	7.23546E-08	0.000528165	Up	1480	63.5	16.77	63.5	16.77	promoter	1157
NM_001106224	Ube2s	chr1	3.490902339	3.33098E-10	1.98956E-05	Up	2140	93.91	26.83	93.91	26.83	promoter	856
NM_001011904	Rnf10	chr12	3.483598875	2.39023E-08	0.000287524	Up	1700	74.24	21.24	74.24	21.24	promoter	1029
NM_001011895	Trmt2a	chr11	3.399141631	9.35996E-11	7.76529E-06	Up	2020	102.86	30.19	102.86	30.19	promoter	710
NM_001106540	Pigt	chr3	3.345560507	4.14027E-10	2.28481E-05	Up	2260	97.49	29.07	97.49	29.07	promoter	666
NM_001104630	Sephs1	chr17	3.330879504	4.93826E-09	0.00011209	Up	2760	85.87	25.71	85.87	25.71	promoter	-1102
NM_001107378	Zfp608	chr18	3.291867805	3.65648E-09	9.26823E-05	Up	3100	88.55	26.83	88.55	26.83	promoter	-465
NM_001037658	Rnf130	chr10	3.269813001	8.82413E-08	0.000581966	Up	1640	73.34	22.36	73.34	22.36	promoter	1602
NM_001191085	Mical3	chr4	3.266327396	4.38639E-08	0.000412187	Up	1760	76.92	23.48	76.92	23.48	promoter	-800
NM_052809	Cdo1	chr18	3.192156863	5.88175E-09	0.000123946	Up	2060	89.44	27.95	89.44	27.95	promoter	1034
NM_001107694	Rfx5	chr2	3.157303371	3.49295E-08	0.000348884	Up	2080	81.39	25.71	81.39	25.71	promoter	435
NM_212511	Rpl35	chr3	3.008227631	2.88449E-08	0.000312119	Up	2120	87.65	29.07	87.65	29.07	promoter	-920
NM_001105812	Slc43a2	chr10	2.218629715	2.91291E-08	0.000314809	Up	2060	144	64.85	144	64.85	promoter	510
