# Supplementary material for: Sodium butyrate mediates histone crotonylation and alleviated neonatal rats hypoxic–ischemic brain injury through gut–brain axis
Source: Front Microbiol. 2022 Oct 20;13:993146. doi: 10.3389/fmicb.2022.993146 (PMC9631217; doi:10.3389/fmicb.2022.993146)
Supplement: Supplementary file 1 [file Data_Sheet_1.ZIP › Supplementary Table/Table S6.pdf]

Table S6 ChIP-seq enrichment analysis of promoter regions in Sham and HIBD group

RefSeq_name	GeneSymbol	chr	Fold change	P-value	FDR	HIBD vs Sham	Length	HIBD_IP.cnt	Sham_IP.cnt	HIBD_IP.avg	Sham_IP.avg	Peak_classification	Peak_To_TSS
NR_037363	Mir1188	chr6	-217.3	4.34251E-08	0.000323815	Down	680	0	21.63	0	21.63	promoter	1793
NM_001044242	Pdhx	chr3	-209	7.87462E-08	0.00046787	Down	800	0	20.8	0	20.8	promoter	1480
NM_001107188	Rasal2	chr13	-209	7.87462E-08	0.00046787	Down	780	0	20.8	0	20.8	promoter	492
NM_001025673	Cenpu	chr16	-21.83846154	2.80561E-08	0.000263169	Down	1400	1.2	28.29	1.2	28.29	promoter	-879
NM_001106584	Rpa3	chr4	-21.2	4.90416E-08	0.000352637	Down	1000	1.2	27.46	1.2	27.46	promoter	769
NM_001177442	Glcci1	chr4	-21.2	4.90416E-08	0.000352637	Down	1000	1.2	27.46	1.2	27.46	promoter	-1042
NM_032990	Gria3	chrX	-15.016	1.13404E-09	3.90902E-05	Down	1720	2.4	37.44	2.4	37.44	promoter	-1463
NM_001112742	Gria3	chrX	-15.016	1.13404E-09	3.90902E-05	Down	1720	2.4	37.44	2.4	37.44	promoter	-1463
NM_001108018	Cbll1	chr6	-13.48247978	4.74387E-12	9.55111E-07	Down	1560	3.61	49.92	3.61	49.92	promoter	-650
NM_001025724	Pnldc1	chr1	-12.688	4.81223E-08	0.000352637	Down	1360	2.4	31.62	2.4	31.62	promoter	-1725
NM_001137647	Sh3bgrl2	chr8	-10.18737271	4.67871E-11	5.18096E-06	Down	2100	4.81	49.92	4.81	49.92	promoter	-350
NM_001108262	Zc3h7a	chr10	-10.11859838	1.20403E-08	0.000165296	Down	2440	3.61	37.44	3.61	37.44	promoter	-574
NM_001109093	Grb10	chr14	-9.67115903	3.36267E-08	0.000303283	Down	1420	3.61	35.78	3.61	35.78	promoter	1971
NM_001168612	Crlf3	chr10	-9.447439353	5.6084E-08	0.000386642	Down	1380	3.61	34.95	3.61	34.95	promoter	-1962
NM_001007750	Chpt1	chr7	-9.447439353	5.6084E-08	0.000386642	Down	1400	3.61	34.95	3.61	34.95	promoter	-441
NM_017068	Lamp2	chrX	-9.447439353	5.6084E-08	0.000386642	Down	1000	3.61	34.95	3.61	34.95	promoter	409
NM_001172103	Pim2	chrX	-9.3401222	5.95247E-10	2.51772E-05	Down	2320	4.81	45.76	4.81	45.76	promoter	-1015
NM_001037643	Ube2z	chr10	-7.985743381	3.26954E-08	0.000297068	Down	1080	4.81	39.11	4.81	39.11	promoter	-707
NM_001013876	Fam161a	chr14	-7.208032956	6.8745E-13	2.09999E-07	Down	2020	9.61	69.89	9.61	69.89	promoter	-368
NM_001100565	Peli1	chr14	-6.273597811	2.22886E-08	0.000232567	Down	1700	7.21	45.76	7.21	45.76	promoter	-450
NM_001100574	Zkscan8	chr17	-6.074030552	4.1863E-09	8.24122E-05	Down	2040	8.41	51.59	8.41	51.59	promoter	141
NM_001107683	Slitrk3	chr2	-6.074030552	4.1863E-09	8.24122E-05	Down	2540	8.41	51.59	8.41	51.59	promoter	-1179
NM_057120	Nudt1	chr12	-6.046511628	5.67097E-08	0.000389441	Down	1700	7.21	44.1	7.21	44.1	promoter	1408
NM_001106002	Polr2b	chr14	-6.046511628	5.67097E-08	0.000389441	Down	1660	7.21	44.1	7.21	44.1	promoter	1647
NM_001047093	Klhl5	chr14	-5.876373626	9.2929E-11	7.76639E-06	Down	1660	10.82	64.07	10.82	64.07	promoter	827
NM_017296	Kcnj2	chr10	-5.780258519	1.6686E-08	0.000194753	Down	1980	8.41	49.09	8.41	49.09	promoter	-1956
NM_001014092	Paqr5	chr8	-5.682726204	2.63642E-08	0.000259082	Down	1520	8.41	48.26	8.41	48.26	promoter	65
NM_001106584	Rpa3	chr4	-5.571428571	5.71848E-10	2.51772E-05	Down	1920	10.82	60.74	10.82	60.74	promoter	-1411
NM_001177442	Glcci1	chr4	-5.571428571	5.71848E-10	2.51772E-05	Down	1920	10.82	60.74	10.82	60.74	promoter	1138
NM_001017461	Ogdh	chr14	-5.226072607	6.30667E-10	2.61072E-05	Down	2000	12.02	63.24	12.02	63.24	promoter	-380
NM_001106343	Zfp275	chr1	-5.151390319	4.57357E-08	0.000338481	Down	1920	9.61	49.92	9.61	49.92	promoter	704
NM_031347	Ppargc1a	chr14	-5.004504505	4.33096E-10	2.05171E-05	Down	2920	13.22	66.56	13.22	66.56	promoter	137
NM_001108282	Mrm3	chr10	-4.961538462	2.00716E-08	0.000212343	Down	1260	10.82	54.08	10.82	54.08	promoter	383
NM_001014227	Glod4	chr10	-4.961538462	2.00716E-08	0.000212343	Down	1260	10.82	54.08	10.82	54.08	promoter	-458
NM_172331	Chmp3	chr4	-4.80952381	4.79934E-08	0.000352637	Down	1320	10.82	52.42	10.82	52.42	promoter	1045
NM_001025146	Ndufs4	chr2	-4.763085399	4.48676E-10	2.10303E-05	Down	2220	14.42	69.06	14.42	69.06	promoter	593
NM_033298	Yes1	chr9	-4.745049505	1.34332E-08	0.000172466	Down	1480	12.02	57.41	12.02	57.41	promoter	285
NM_053723	Lancl1	chr9	-4.745049505	1.34332E-08	0.000172466	Down	1580	12.02	57.41	12.02	57.41	promoter	859
NM_057136	Epn1	chr1	-4.476584022	3.7597E-09	7.8925E-05	Down	2060	14.42	64.9	14.42	64.9	promoter	-322
NR_032143	Mir664-2	chr13	-4.47029703	7.41323E-08	0.000464442	Down	1940	12.02	54.08	12.02	54.08	promoter	486
NM_001014212	Rsl24d1	chr8	-4.317567568	4.75628E-08	0.00035171	Down	1540	13.22	57.41	13.22	57.41	promoter	697
NM_001010963	Zc3h15	chr3	-4.082061069	1.89459E-08	0.000204182	Down	1480	15.62	64.07	15.62	64.07	promoter	662
NM_001110138	Flad1	chr2	-3.92302799	6.3673E-08	0.000421716	Down	1180	15.62	61.57	15.62	61.57	promoter	752
NM_001108953	Zbtb6	chr3	-3.741287655	8.63423E-08	0.000488177	Down	2080	16.83	63.24	16.83	63.24	promoter	-4
NM_001009290	Ndufc2	chr1	-3.652216269	8.88849E-09	0.000131895	Down	2060	20.43	74.88	20.43	74.88	promoter	1617
NM_001135020	Sec61g	chr14	-3.565577543	7.89643E-09	0.000120816	Down	2400	21.63	77.38	21.63	77.38	promoter	12
NM_001079887	Ing4	chr4	-3.534919814	6.8528E-08	0.000446378	Down	2700	19.23	68.23	19.23	68.23	promoter	739
NM_001107517	Cyfip1	chr1	-3.254538279	1.58237E-08	0.000189494	Down	2560	25.24	82.37	25.24	82.37	promoter	1931
NM_198777	Tmem138	chr1	-3.040099558	4.05173E-10	1.99408E-05	Down	2900	36.06	109.83	36.06	109.83	promoter	-190
NM_017059	Bax	chr1	-2.476631904	6.88607E-09	0.000119312	Down	2460	51.68	128.14	51.68	128.14	promoter	1124
