# Supplementary material for: Sodium butyrate mediates histone crotonylation and alleviated neonatal rats hypoxic–ischemic brain injury through gut–brain axis
Source: Front Microbiol. 2022 Oct 20;13:993146. doi: 10.3389/fmicb.2022.993146 (PMC9631217; doi:10.3389/fmicb.2022.993146)
Supplement: Supplementary file 1 [file Data_Sheet_1.ZIP › Supplementary Table/Table S8.pdf]

Table S8 The primer sequences of qPCR

Gene	Sequence (5 - 3)
Bdnf	F: CAACGAAGAAAACCATAA
	R: GTGACCCACTCGCTAATA
Gdnf	F: GCTGAAGACCACTCCCTCG
	R: ACGTCATCAAACTGGTCAGGA
Cdnf	F: TGCTGTGTGTGCTCTCCTAA
	R: GTTGTTGTAGTGCTGGCTCA
Manf	F: CTCACAAGTGCACAGAACCC
	R: TGAGGTCACAGTGAGTTCCC
