# Supplementary material for: Sodium butyrate mediates histone crotonylation and alleviated neonatal rats hypoxic–ischemic brain injury through gut–brain axis
Source: Front Microbiol. 2022 Oct 20;13:993146. doi: 10.3389/fmicb.2022.993146 (PMC9631217; doi:10.3389/fmicb.2022.993146)
Supplement: Supplementary file 1 [file Data_Sheet_1.ZIP › Supplementary Table/Table S9.pdf]

Table S 9 The primer sequences of CIhP qPCR

**Gene**	**Sequence (5 - 3)**
CIhP-Bdnf	F: CTGGCAGGCTTTGATGAGAC
	R: TCCACACAAAGCTCTCGGAT
CIhP-Gdnf	F: CGCCAATATGCCCGAAGATT
	R: CCCAAACCCAAGTCAGTGAC
CIhP-Cdnf	F: TGTTCCTCGTGGCTTGTTTC
	R: CAGGGAAGGAGTCTGCATCA
CIhP-Manf	F: TAGGGAAACGGAACAGGCTT
	R: CTCACCAGGCCTTCAGATCA
