# Supplementary figures and images for: Sodium butyrate mediates histone crotonylation and alleviated neonatal rats hypoxic–ischemic brain injury through gut–brain axis
Source: Front Microbiol. 2022 Oct 20;13:993146. doi: 10.3389/fmicb.2022.993146 (PMC9631217; doi:10.3389/fmicb.2022.993146)

Fig. S1

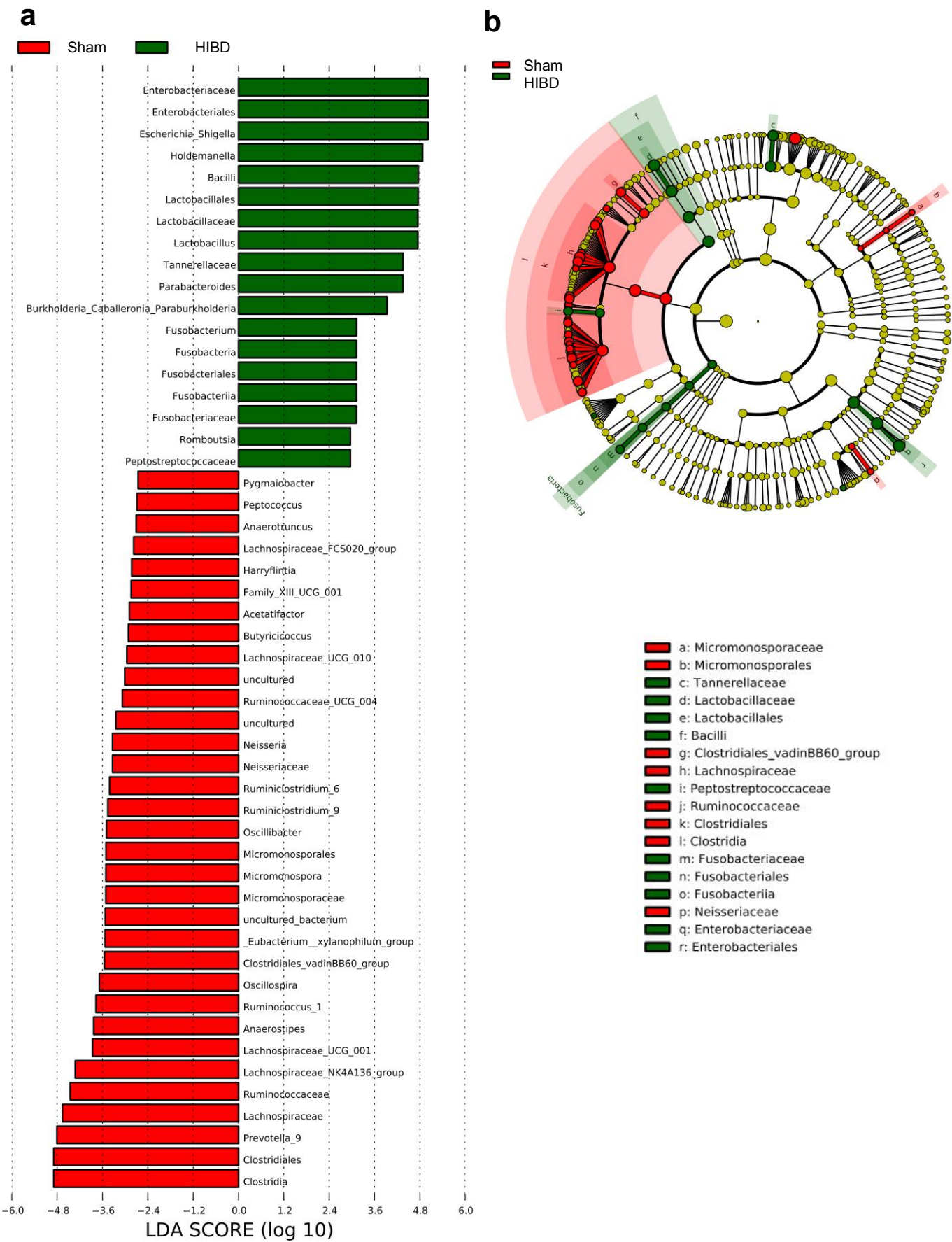

Fig. S2

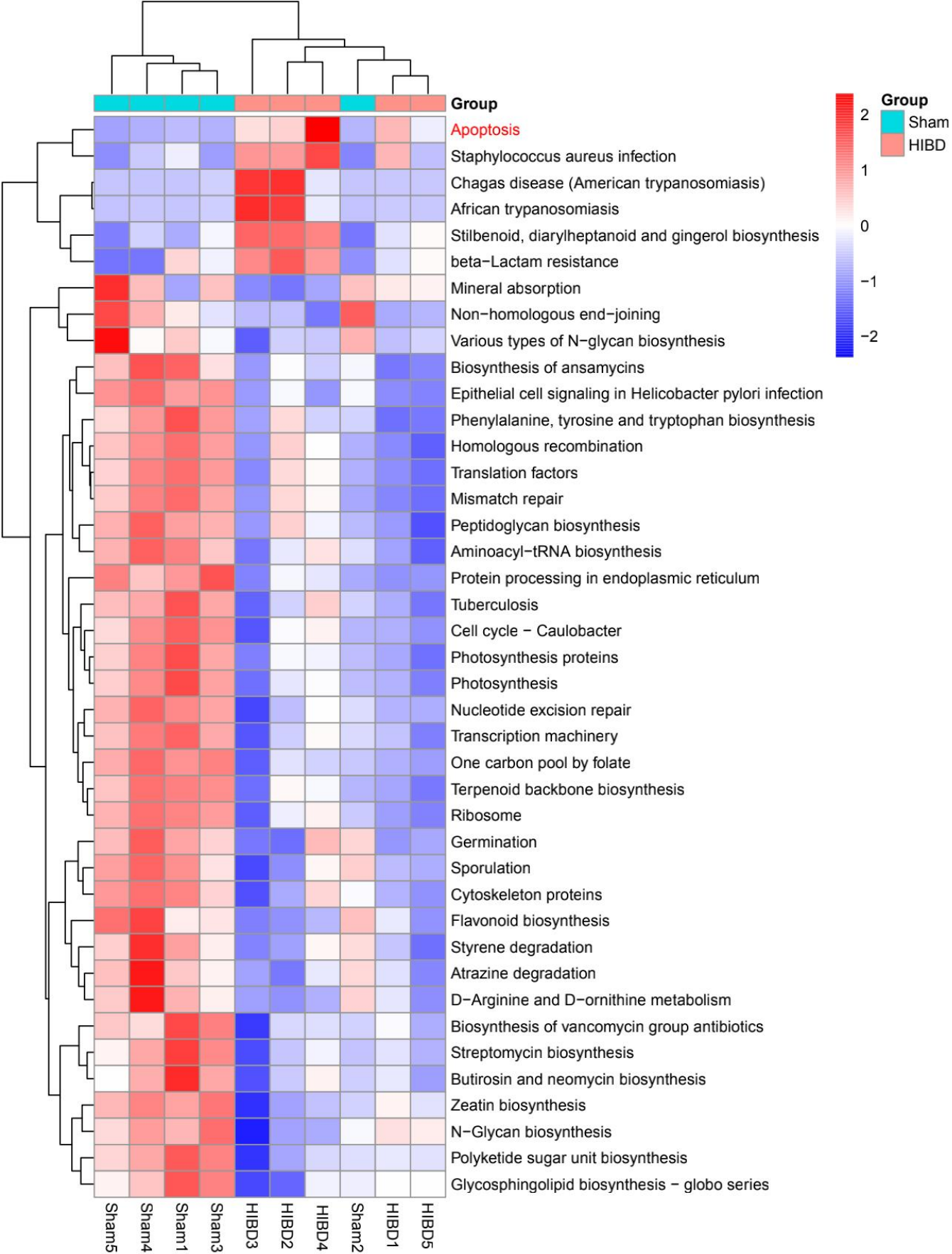

Fig. S3

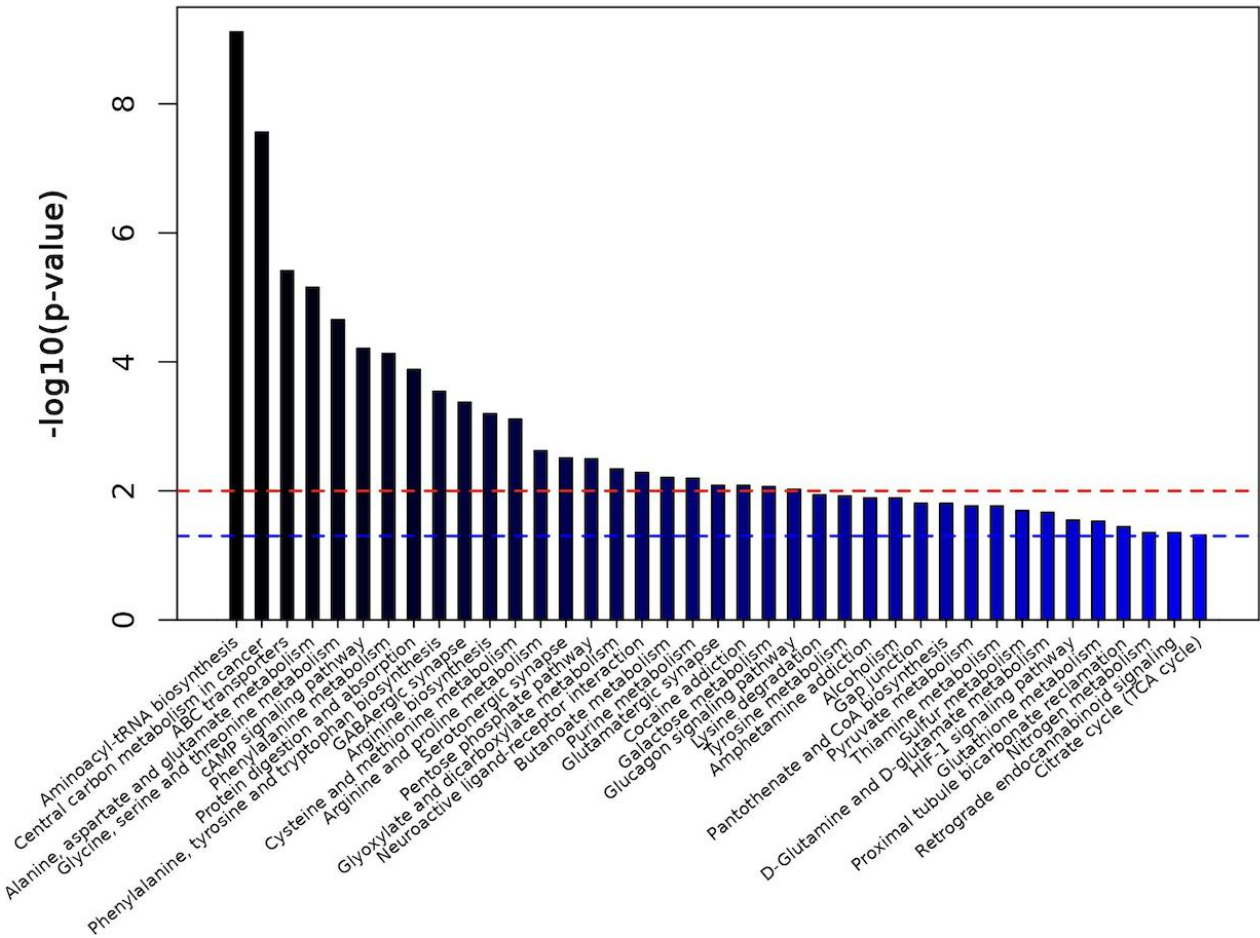

Fig. S4

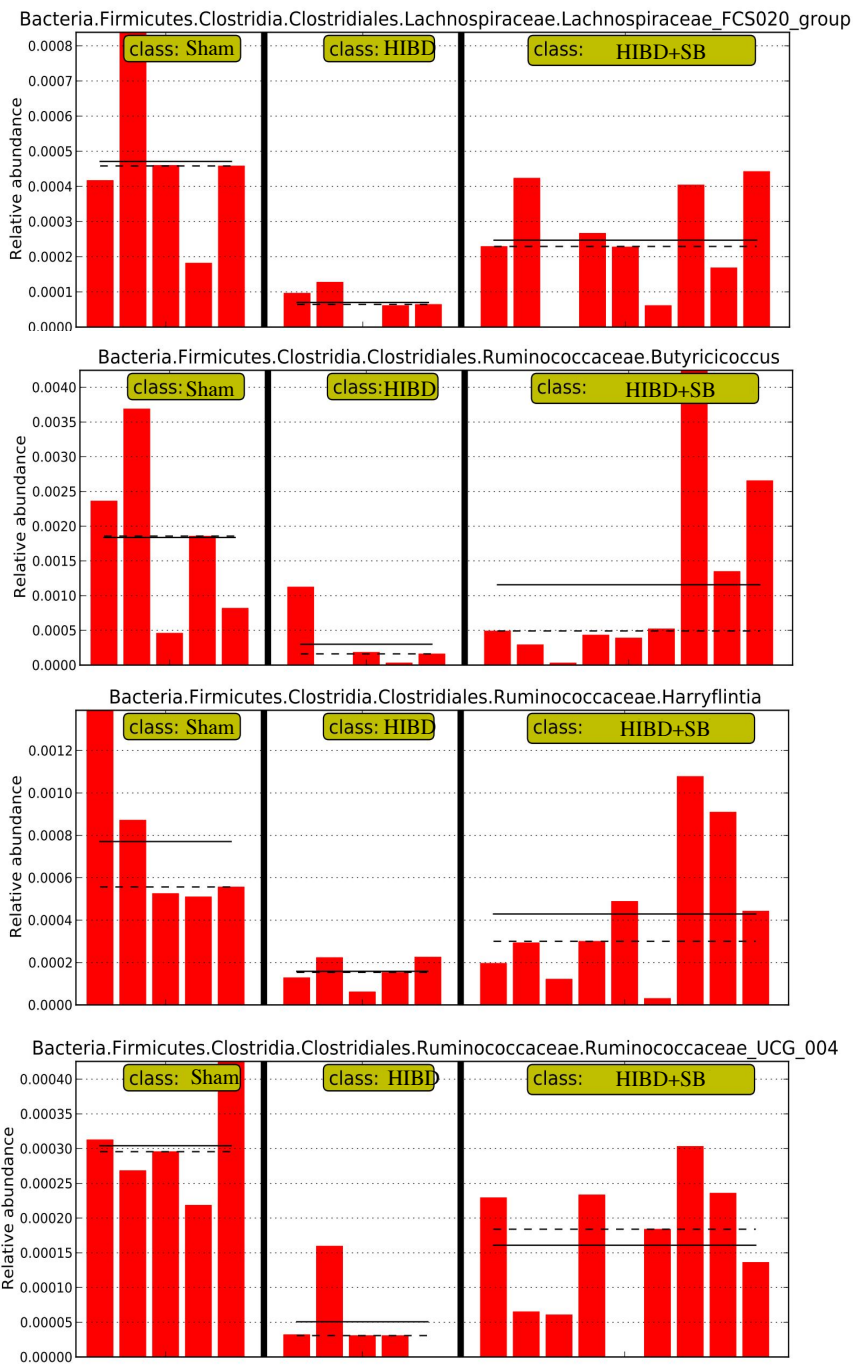

Fig. S5

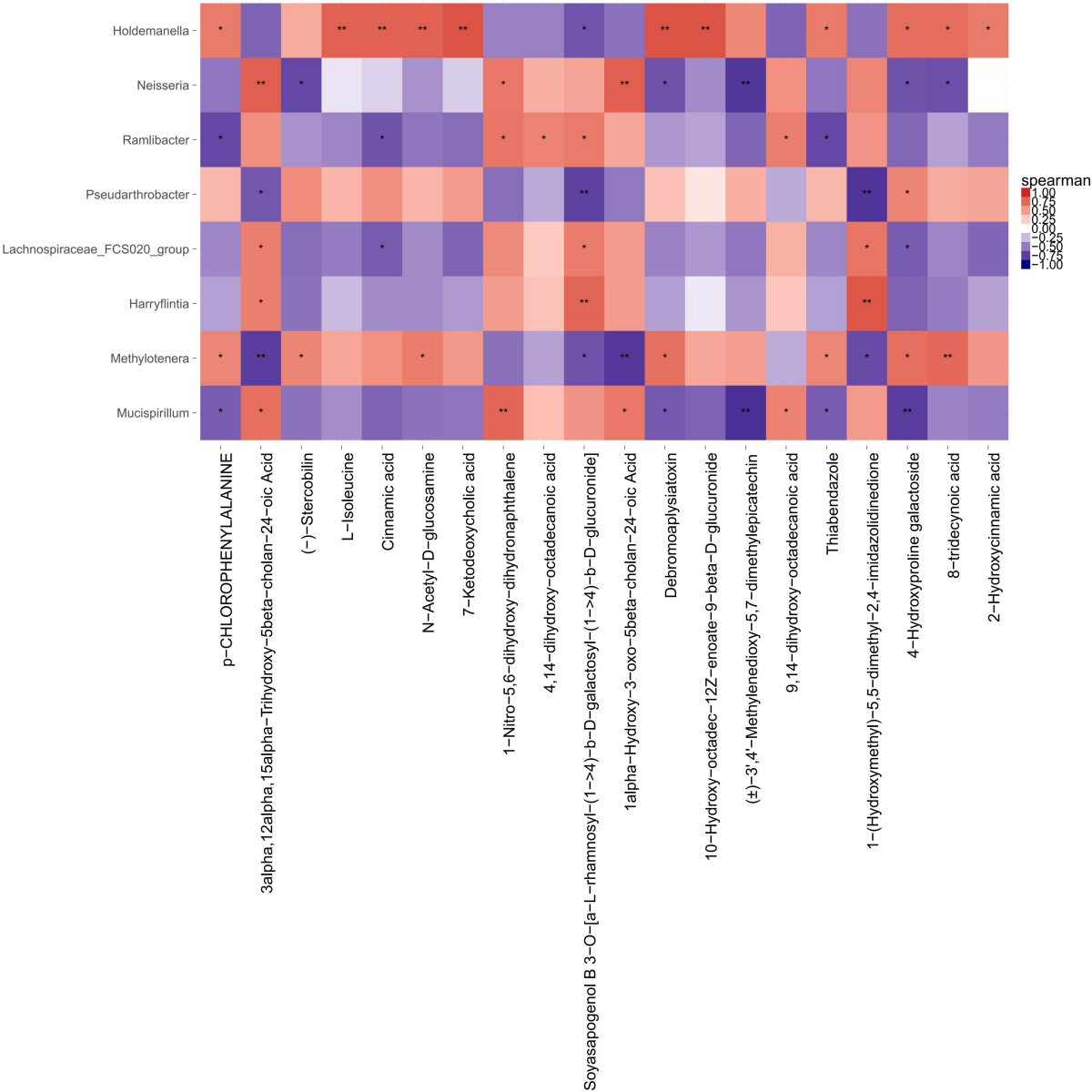

Fig. S6

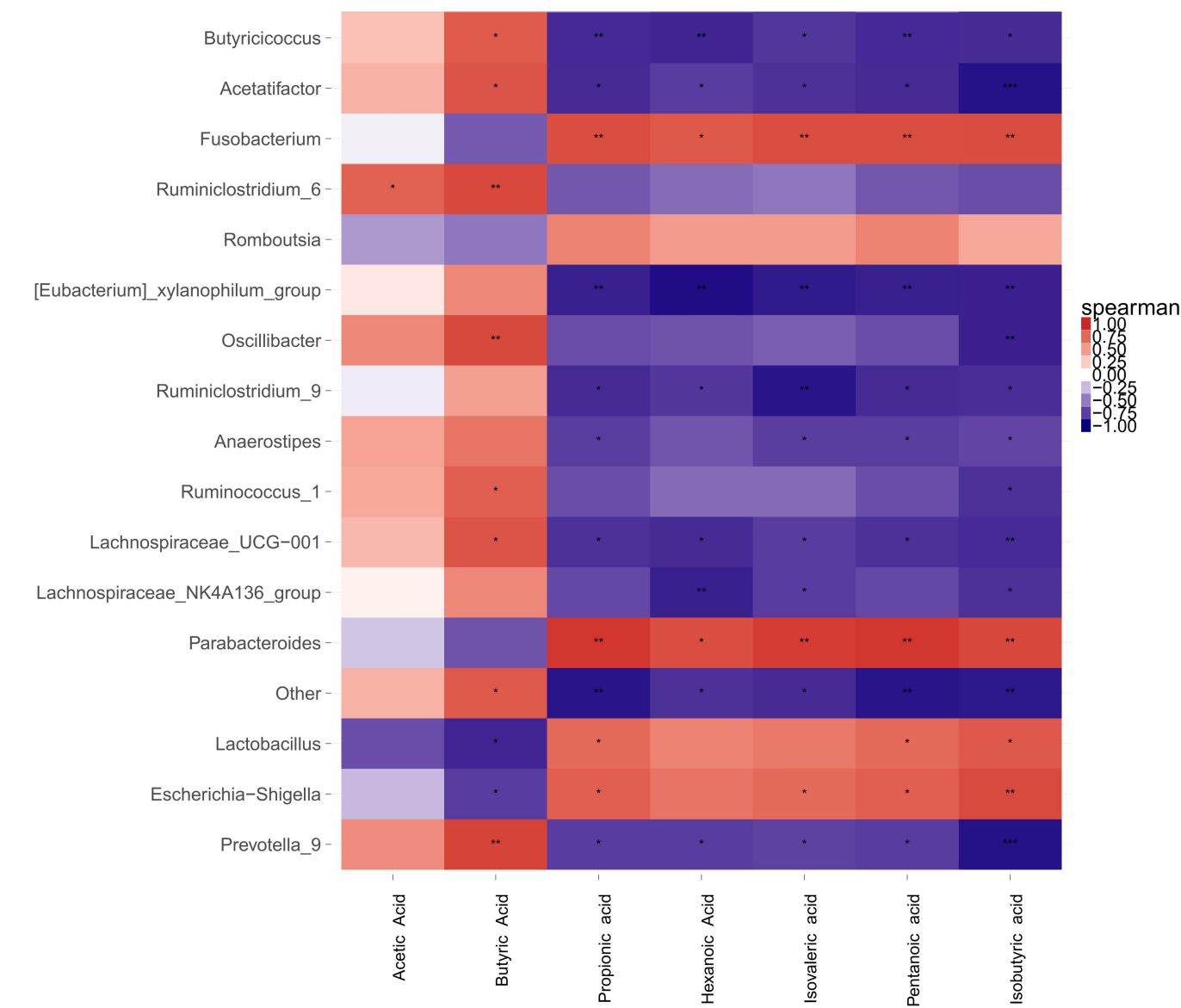

**Fig. S7**

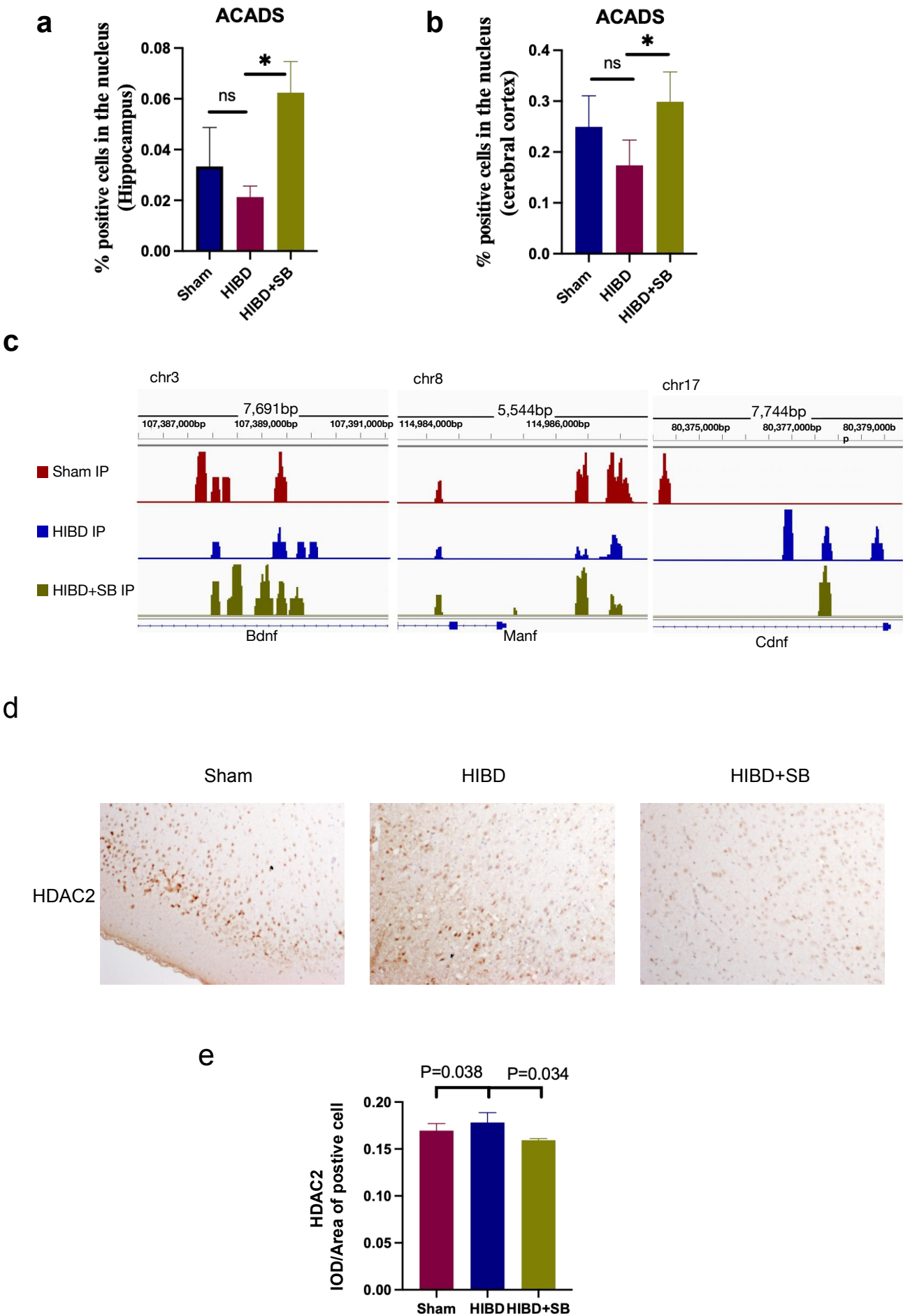

Supplement: Supplementary file 2 [file Data_Sheet_2.PDF]
