# Supplementary material for: Sodium butyrate mediates histone crotonylation and alleviated neonatal rats hypoxic–ischemic brain injury through gut–brain axis
Source: Front Microbiol. 2022 Oct 20;13:993146. doi: 10.3389/fmicb.2022.993146 (PMC9631217; doi:10.3389/fmicb.2022.993146)
Supplement: Supplementary file 1 [file table S4.pdf › Supplementary Table/table S4.pdf]

Table S4 Differentially expressed genes in HIBD and Sham group

Gene	Pvalue	regulation	HIBD	Sham
Gabbr1	1.00707E-40	down	0	110.28487
Ap2b1	1.94694E-27	down	0	44.76406
Dkk3	1.23988E-20	down	0.00287	32.5914
Nfasc	7.28815E-18	down	0	12.58928
Tbl1xr1	6.78378E-17	down	0	14.23806
Fscn1	9.53172E-16	down	0	66.3138
Arhgap21	6.01248E-15	down	0.0062	15.68469
Ryr2	2.2423E-14	down	0	5.70647
Tpi1	7.2929E-14	down	0.60727	126.63211
Chd8	7.31263E-14	down	0	9.33918
Slc2a3	2.69045E-13	down	0	19.37478
Ddr1	2.99873E-13	down	0.00391	21.94384
Zcchc12	4.17667E-13	down	0	34.49004
L1cam	9.45284E-13	down	0.07112	22.83391
Fbxw11	1.08352E-12	down	0	16.92724
Fermt2	3.34641E-12	down	0.03133	28.43424
LOC681410	3.75203E-12	down	0.17511	110.15795
Ndufs7	5.85414E-11	down	0	53.36427
Ssx2ip	6.02693E-11	down	0	15.65442
Lamb1	1.72077E-10	down	0	8.54766
LOC103690017	2.84192E-10	down	0	12.40156
LOC100911581	3.99544E-10	down	0	12.61913
Brd4	4.72511E-10	down	0.0143	9.60472
Plxna3	7.7383E-10	down	0	5.78768
Mt-atp6	9.9584E-10	down	3022.69801	11131.66649
Slit2	1.01806E-09	down	0	5.83843
Wbp11l1	1.49366E-09	down	0	16.12457
Tmem229b	1.511E-09	down	0	10.67726
Megf9	2.00182E-09	down	0.02201	17.40019
Plat	2.721E-09	down	0	14.98904
Ampd2	7.09497E-09	down	0.01283	13.83783
Pds5b	8.58963E-09	down	0	4.81279
LOC108348287	9.4145E-09	down	0	68.37841
Nploc4	1.5064E-08	down	0.10537	17.52443
Slain2	1.70398E-08	down	0	6.60555
Wbp4	1.97809E-08	down	0	9.9812
Dopey1	2.0728E-08	down	0.01494	6.77966
Acox1	3.39239E-08	down	0	7.60841
Dctn1	3.81878E-08	down	8.50664	101.77235
Ppm1f	5.56919E-08	down	0	5.89283
Col6a1	6.93166E-08	down	0	8.72363
Pomk	7.05467E-08	down	0.00492	10.75542
Snx6	7.40675E-08	down	0	13.7685
Jup	7.97399E-08	down	0.00837	9.9785
Dock9	8.56587E-08	down	0	3.39749
Adcy2	1.10793E-07	down	0.74625	36.81042
Birc2	1.2844E-07	down	0.00442	9.00585
Znf740	1.6352E-07	down	0	6.56452
Sorcs2	1.64286E-07	down	0.02516	8.00666
LOC103689943	1.69846E-07	down	0.00754	4.75137
Grm4	2.23148E-07	down	0	9.45498
Sarnp	2.85104E-07	down	0	19.98282
Vav2	3.78013E-07	down	0	4.11982
LOC100912228	4.03613E-07	down	0	34.70447
Rbbp6	5.32469E-07	down	0	3.37863
Dhrs7b	5.51657E-07	down	0	16.55051
Ywhag	5.55119E-07	down	2.03441	73.85347
Ddx42	5.7262E-07	down	0.00707	6.65546
Reck	6.10938E-07	down	0	4.69243
Vrk3	7.04674E-07	down	0	11.18713
Casp8ap2	1.02479E-06	down	0	2.48281
Ankfy1	1.15922E-06	down	0	2.47534
Dcx	1.21919E-06	down	0	1.99638
Acvr1	1.26149E-06	down	0.00476	7.10472
Cxxc1	1.53587E-06	down	0	6.44761
Sssca1	1.62502E-06	down	0.01364	19.54985
Zfp384	1.85136E-06	down	0	6.16008
Arhgap39	1.99612E-06	down	0	3.50049
Dzip1l	2.12254E-06	down	0	4.16898
Cdc45	2.46256E-06	down	0.1057	8.94027
RGD1307100	2.5369E-06	down	0.01504	2.68308
Foxj2	2.64261E-06	down	0	3.49811
LOC100911516	3.02729E-06	down	0.37338	75.80316
Mto1	3.05367E-06	down	0	7.48918
Ctif	3.41483E-06	down	0	2.41637
AABR07015067.1	3.5336E-06	down	0	31.76584
Rps8	3.56722E-06	down	2.60243	114.93728
Mmadhc	3.58585E-06	down	0	10.29465
LOC100911615	3.72927E-06	down	0	6.35559
Rbm39	4.30292E-06	down	0	7.0448
Nav1	4.85136E-06	down	0.21971	10.52005
Arnt2	4.89492E-06	down	0.2912	12.48011
Slc1a3	4.95115E-06	down	19.8956	131.49734
Sf1	5.49907E-06	down	0	4.99978
Cic	5.86902E-06	down	0.09532	8.70028
Cdh7	6.53791E-06	down	0	1.99694
Cops7a	6.96674E-06	down	0.14744	20.57464
Rin2	7.88658E-06	down	0.0197	5.60971
AABR07024139.1	8.42795E-06	down	0	2.66769
Plch1	8.65201E-06	down	0	1.91943
Wars	8.88259E-06	down	0	6.6599
Pitpna	9.22168E-06	down	7.92001	107.29599
RGD1308428	9.61839E-06	down	0.00303	3.16545
Sh3bp1	1.00334E-05	down	0	4.52597
St3gal1	1.03543E-05	down	0.03089	5.32044
Max	1.08133E-05	down	0	5.74763
Tor1aip2	1.09301E-05	down	0	2.24535
Alk	1.23773E-05	down	0	2.32018
Dclre1b	1.25811E-05	down	0	2.7722
Dcun1d2	1.27191E-05	down	0	4.08523
Mff	1.52919E-05	down	0.00754	7.14986
LOC100911766	1.59506E-05	down	0	2.91274
Myo5a	1.63118E-05	down	0	1.89291
Cntrl	1.77496E-05	down	0	1.45162
Paics	2.04714E-05	down	0	5.00446
Maml1	2.04714E-05	down	0	1.79521
Kcnk4	2.115E-05	down	0.13244	21.64786
Ppig	2.41368E-05	down	0.1053	11.24754
Gatd1	2.54022E-05	down	0	6.20862
Sap130	2.69918E-05	down	0.01739	4.53698
Carmil1	2.71168E-05	down	0	1.82935
Ccnt2	2.73177E-05	down	0.01017	3.0729
Pfdn6	2.81071E-05	down	0	15.54096
AC134224.1	3.00192E-05	down	2.65484	47.38925
Kif16b	3.07673E-05	down	0	1.64048
Dot1l	3.09546E-05	down	0	1.4718
Xkr8	3.25004E-05	down	0	3.29737
LOC100912585	3.29005E-05	down	0	1.62595
Tmem255a	3.41349E-05	down	0	6.48856
Zfp667	3.51828E-05	down	0.00426	3.34712
Camk2n2	3.79615E-05	down	0.68454	68.34814
NEWGENE_13592	4.33046E-05	down	0	3.30883
Ccne1	4.61691E-05	down	0	4.074
Ercc8	4.79926E-05	down	0	4.13923
Rps6ka3	5.02264E-05	down	0	1.18642
Gpx4	5.05719E-05	down	0.77287	54.39542
LOC100910750	5.29285E-05	down	0	5.81777
AABR07019086.1	5.54321E-05	down	0	1.84865
LOC103694903	6.35022E-05	down	6.41479	108.71375
Man2c1	6.59787E-05	down	0.07597	7.86999
Focad	6.82591E-05	down	0.33054	9.14668
Trim65	7.68721E-05	down	0	2.3065
Mdm1	8.27682E-05	down	0.00468	2.96705
Rasgrp3	8.42707E-05	down	0	1.56602
Lmo4	8.73387E-05	down	0	4.17196
Slc4a5	8.9749E-05	down	0.00426	2.64372
Git1	9.25278E-05	down	0	2.089
Smarcb1	9.38818E-05	down	0	1.75348
Sacs	9.85402E-05	down	0.37376	6.08178
Sstr1	0.00010327	down	0.06625	6.13198
Nell2	0.0001049	down	25.44494	131.18934
AABR07062481.1	0.000108808	down	0	3.86831
Vps53	0.000108808	down	0	2.33665
LOC100911769	0.000112124	down	0	2.48135
Tmem38a	0.000116442	down	0	3.22236
Prkcz	0.00011646	down	0.08002	6.82136
Stambp	0.00011733	down	0	3.46358
Snx12	0.000118225	down	0	9.03194
LOC103690317	0.000118225	down	0	3.14297
Wscd1	0.000120059	down	0.91338	22.76945
March7	0.000123774	down	0	2.51462
Ahi1	0.000126666	down	0	1.89466
Jak3	0.000134785	down	0	1.56987
Lca5	0.000137991	down	0	1.85471
Prrt4	0.000137991	down	0	1.99301
Ntn3	0.000139143	down	0.00592	3.2557
Snx14	0.000148177	down	0	2.05141
Mfsd14a	0.000149363	down	0	3.0497
Asap2	0.000159598	down	0.01964	4.0139
Carmil1	0.000169232	down	0.0027	1.4049
Dnal1	0.000176986	down	0	1.88267
Rgs14	0.000181436	down	0.15835	9.56949
LOC100359687	0.000182895	down	0	5.64475
Tmem9	0.000197047	down	0	3.57919
Flad1	0.000204889	down	0.04022	5.13968
Amer2	0.000207193	down	0	2.90746
Casp3	0.000217957	down	0	2.04022
Kcnh1	0.000219814	down	0	1.24472
Smad2	0.000225497	down	0	1.89001
Yap1	0.00022938	down	0	3.50021
LOC102550026	0.000239431	down	0	1.60316
Htatip2	0.000240379	down	0.01039	4.86542
LOC103690164	0.000245709	down	0	3.03955
Mtfr1l	0.000247844	down	0	2.61119
Spag5	0.00025438	down	0	1.21944
Arhgef1	0.00026112	down	0	1.47878
LOC108348055	0.000263413	down	0	6.11184
Wdr31	0.000272825	down	0	3.4906
Dyrk2	0.000273119	down	0.37041	9.12002
Gas2	0.00027524	down	0	2.05421
Zbtb9	0.00027524	down	0	2.31201
Mta1	0.000292408	down	1.21709	22.5074
Tdrd7	0.000297958	down	0.07632	5.4219
Acvrl1	0.000309355	down	0.0041	1.779
Rhot1	0.000310971	down	0.21252	10.46406
Sept5	0.000316702	down	0.09066	11.22856
Urgcp	0.000339408	down	0.02056	3.02279
Sec31a	0.00036998	down	0.15724	7.39582
Mx2	0.000392192	down	0.01185	2.92387
Mrps12	0.000392995	down	0	5.7007
RT1-A1	0.000424257	down	0	2.58578
Nxph1	0.00043025	down	0.19894	11.16892
Nle1	0.000441037	down	0	2.24657
Ppfia2	0.000460245	down	0.30391	10.0009
Alkbh8	0.000463187	down	0	1.56538
Eif4b	0.000465277	down	5.32807	41.11217
RGD1564887	0.000477144	down	0	2.55175
LOC108348139	0.000501265	down	0.01105	4.10422
Nfkb1	0.000529828	down	0.00372	1.35592
Cldn2	0.000542468	down	0.00928	2.07007
Zkscan3	0.000547004	down	0.0056	2.01956
Ebf1	0.00057296	down	0	1.15877
Spock2	0.00058336	down	0.01125	3.97082
Pot1	0.000588561	down	0.01594	2.68938
Mycbpap	0.000603815	down	0	1.20666
Epb41l1	0.000606296	down	4.50345	43.36238
Tmco4	0.000616749	down	0	1.04909
Arhgap22	0.000616749	down	0	1.27626
Rusc2	0.000618234	down	1.96404	21.46406
Tsta3	0.000650673	down	0	2.81134
Tsku	0.000664912	down	0	1.31125
Tmem231	0.000674104	down	0.03577	3.613
Acad9	0.000674303	down	0.7535	17.37835
AABR07067506.1	0.000678723	down	0.74677	40.31569
Rtkn	0.000694617	down	0	1.77627
Fbxo31	0.000702309	down	0	2.17234
Sema6c	0.000702309	down	0	1.00828
Nfil3	0.000718029	down	0	1.67156
Ilvbl	0.00072606	down	0	1.23948
Chd2	0.000728871	down	0.00666	1.0504
Slfn13	0.000741272	down	0.00426	1.38547
Itgb5	0.000746831	down	0.03126	2.86106
LOC108348298	0.000750863	down	0	2.32862
LOC103690141	0.00077678	down	0	1.87642
Spats2l	0.00077678	down	0	1.39354
LOC688583	0.000785678	down	0	2.08342
RGD1565222	0.000821098	down	0.0049	1.53376
Msantd2	0.000822632	down	0	1.86724
Sirt4	0.000841965	down	0	2.16064
Bace2	0.000861899	down	0	1.72594
Capg	0.0008721	down	0	2.05911
Cep19	0.000892983	down	0	1.9491
Pigc	0.000892983	down	0	2.16334
Impad1	0.000914531	down	0	1.74473
AABR07059243.1	0.000914531	down	0	11.88638
LOC103690038	0.000995606	down	0	1.40638
Pla2g4a	0.001020516	down	0	1.00705
Lrrc20	0.001059464	down	0	1.10873
Smndc1	0.001086541	down	0	2.08393
Agpat4	0.001101818	down	4.06701	43.73597
RT1-CE5	0.001128924	down	0	1.70801
Srr	0.001128924	down	0	2.32358
Tmem39a	0.001143545	down	0	1.31688
Clip3	0.001155964	down	20.41745	88.29619
Nae1	0.001164647	down	0.30706	11.69237
Tpx2	0.00116831	down	0.00907	1.56616
Itgb4	0.001179426	down	0.01486	1.2704
Ocln	0.001205115	down	0.0389	2.77321
Mgat5b	0.001217021	down	0.02549	2.15816
Tug1	0.001250588	down	0.26635	10.37679
Mitf	0.001253402	down	0	1.22385
Ubr2	0.001263625	down	0.11109	4.20458
Kcnip4	0.001266177	down	0.09746	5.83315
Mff	0.0012874	down	0	1.20641
Adcy5	0.001290012	down	8.48588	43.65847
Hivep2	0.001296031	down	6.49222	29.2868
Prtfdc1	0.001302828	down	0.0654	6.43161
Zfp189	0.001322661	down	0	1.2819
Mical2	0.001324213	down	11.95514	58.01665
AC117058.1	0.001341083	down	0.0106	2.76568
Med7	0.001359249	down	0	2.17687
Rpl22	0.001359249	down	0	5.49848
St7l	0.001378061	down	0	1.04033
LOC103690035	0.001378061	down	0	1.96238
Med8	0.001416764	down	0	3.87264
Csf1	0.001436674	down	0	1.31621
Ddx59	0.001456969	down	0	1.20582
AABR07042936.2	0.001477658	down	0	9.91023
Gpbp1l1	0.001503787	down	0.12711	5.87807
Cttn	0.001513981	down	0.84448	14.49991
Intu	0.001542197	down	0	1.53923
Armcx3	0.00156457	down	0	1.06532
LOC100365958	0.001610674	down	0	1.40483
Top3b	0.001651714	down	0.00486	1.16886
Herc1	0.001678525	down	0.30596	3.85743
RGD1565472	0.001683413	down	0	4.84922
Smarcad1	0.001731451	down	0.16789	4.86135
Tef	0.001760772	down	0	1.9445
Txnrd3	0.001832195	down	0.00517	1.19312
AABR07028989.1	0.001843148	down	0	1.16359
Agpat1	0.001871319	down	0.01817	4.15954
Akr1a1	0.001923976	down	63.18021	252.48253
Hapln4	0.001928543	down	1.57516	17.52992
Dcakd	0.001961553	down	0	1.20898
Ppp1cc	0.001988055	down	0.09628	5.88289
Amy1a	0.002024753	down	0	1.32614
Il11ra1	0.002090809	down	0	1.25335
Clpb	0.002121953	down	0.77178	15.25625
Mob2	0.002124959	down	0	1.79248
Clec11a	0.002132629	down	0.06601	3.38821
Abhd14a	0.002159886	down	0	1.4976
Fam193b	0.002159886	down	0	1.18122
Agk	0.002232165	down	0	1.13901
Kiaa0408L	0.002233922	down	0.04501	2.3538
Osbpl9	0.002250832	down	0.02725	2.49758
Nrg2	0.002345568	down	0.0386	3.10726
Sec31a	0.002376737	down	0.27025	6.07553
Gnb3	0.002387112	down	0	1.3073
Mgst1	0.002467501	down	0.01513	3.09648
Stard10	0.002475927	down	0.23	9.64752
Aurkc	0.002513271	down	0	1.79931
Fastk	0.002557377	down	0	1.23039
Mtrex	0.002561329	down	0.03999	2.24926
Tnks2	0.002596009	down	0.7726	11.66176
Sema3f	0.002604538	down	0.05809	2.21834
Zfyve27	0.002605928	down	0.22818	4.94566
Mgat1	0.00267869	down	0.00565	1.17007
Arhgap17	0.002724634	down	0.02949	1.91226
Tmem9b	0.002730789	down	0.45051	14.78885
Exph5	0.002731712	down	0.07163	2.48647
Polr2e	0.002744955	down	0	1.7132
Prpf8	0.002815071	down	8.19219	33.83661
Ngdn	0.002970929	down	0.70561	17.57186
LOC681292	0.003181729	down	0	1.79445
Med24	0.003262811	down	0.27194	6.34184
Gal3st3	0.003303753	down	0.00887	1.6093
Ttf1	0.003381669	down	0.03287	1.6141
Arid1a	0.003448549	down	2.30708	14.32994
Qtrt2	0.003504989	down	0	1.39638
Ano6	0.003538353	down	0.05538	2.04097
Thop1	0.00357506	down	0	1.22955
Dock11	0.003603745	down	0.0518	1.79607
Rplp0	0.003665643	down	34.69538	162.11941
Tyk2	0.003719247	down	0.17624	4.06087
Dock1	0.003736897	down	0.03468	5.17045
Krt26	0.003914839	down	0.10866	3.95694
AABR07062859.1	0.003956209	down	0	4.45978
Kif7	0.004011226	down	0.04285	1.9115
Tbc1d31	0.004131946	down	0.12824	3.83943
Rpe	0.004282801	down	0.15762	6.26756
Fam173b	0.00439602	down	0	1.15336
Fancm	0.004397715	down	0.02603	1.52334
Fmnl2	0.00444401	down	0.02166	1.37357
Lyrm1	0.004492023	down	0	1.26843
Fsd1l	0.005156916	down	0.00772	1.15992
Ppp1cc	0.005177111	down	14.29662	66.39091
Manbal	0.005218404	down	3.5175	39.73908
Egr3	0.005278335	down	0.75541	11.4703
Abca8a	0.005316198	down	0.24693	4.29824
Wnk2	0.005585881	down	3.5238	19.15787
LOC103690064	0.005633385	down	0	1.11923
Ripor1	0.005747641	down	3.32033	21.44613
Cacna2d3	0.005837309	down	0.0351	1.6214
AABR07004112.1	0.006249115	down	0.01135	1.00211
LOC363337	0.006333984	down	0.00884	1.21752
Asph	0.006357227	down	0	1.69092
Atp1a1	0.006396049	down	124.90349	291.74044
Tas1r2	0.006556784	down	0.29736	7.35802
Uap1	0.006999127	down	0.07031	2.65316
LOC103694879	0.007031184	down	0	2.40019
Ppp2ca	0.007214827	down	0	1.39415
Herc4	0.0072303	down	0.41619	6.34168
Tns2	0.007261682	down	0.02052	0.98184
LOC103694875	0.007405074	down	0	1.86698
Cited1	0.007780853	down	0.01689	2.12972
Atg5	0.007782321	down	0.39035	7.86985
Zfp61	0.007818061	down	0.0176	1.12624
AABR07054096.1	0.008018468	down	0	1.3385
Sept7	0.008051962	down	5.74429	34.64167
Zfx	0.008199272	down	0.24947	3.86866
Ppp2r5c	0.008310986	down	0.04218	2.06177
Hspa4l	0.008320232	down	0.19193	7.0359
Dap3	0.008363598	down	0.06397	2.70322
Cby1	0.008429013	down	0.2164	9.11445
Pard3	0.008456598	down	0.06533	1.68281
Ehmt2	0.008560399	down	0.02192	0.9781
Dnajc9	0.008578301	down	0.03506	1.8702
LOC108348106	0.00866684	down	0.0802	4.25828
RGD1304624	0.008702936	down	0	1.07918
Tbc1d9	0.009352924	down	1.17682	9.29839
S100a3	0.009473718	down	0	1.72966
Stx7	0.009480344	down	0.81631	11.31154
Anxa6	0.010013915	down	3.07768	22.29578
Agap3	0.010407734	down	0.41476	6.19721
Armcx1	0.010738585	down	0.26741	5.72906
LOC102555457	0.010763246	down	0.87508	11.75816
Gatm	0.010944111	down	8.08042	40.2969
Crocc	0.010974535	down	0.29733	3.6646
Zfp622	0.011194097	down	0.23399	5.60423
Fam71e1	0.011343629	down	0	1.72866
Cc2d1a	0.01136878	down	1.8226	14.64334
Rln1	0.012084381	down	0	1.32022
Ap3b2	0.01223972	down	8.93943	37.22993
AABR07044383.1	0.012480415	down	0	1.26897
LOC100910245	0.012480415	down	0	1.48229
Ldlrap1	0.012721798	down	0.10914	3.84072
Grm5	0.012768388	down	0.34674	5.31505
AABR07051450.1	0.012860423	down	6.89938	43.22239
Edrf1	0.013137046	down	0.54576	5.57814
Igsf9	0.013404659	down	0.05936	1.47305
Pou2f1	0.013541812	down	0.03618	1.31579
Dnajc8	0.013554817	down	0.07429	2.41365
Timm10b	0.014257955	down	0	1.98355
Znrd1as1	0.014519419	down	0.01484	1.42436
Tshz1	0.014633544	down	0.46978	6.02266
AABR07036087.1	0.014869098	down	0.0555	2.65552
Smc6	0.015027091	down	1.48491	10.65117
Top2b	0.01510463	down	0.71976	13.22407
Pla2g2c	0.015942083	down	0.04721	1.16752
Ing1	0.015949013	down	0.63821	8.70306
C1qtnf1	0.016253394	down	0.06586	2.07115
Fam161a	0.016259532	down	0.0226	1.03758
B4galt6	0.016259532	down	0.03744	1.71848
Pip4k2c	0.016370281	down	3.66837	20.91226
Smarcc1	0.016793213	down	0.03305	0.9735
RGD1304694	0.016960917	down	8.5643	35.72863
Faim	0.016976618	down	0	1.34053
Eml1	0.017068238	down	0.03623	1.18277
Use1	0.017200117	down	1.23973	16.03543
Cdh24	0.017221009	down	0.43506	6.07935
Ranbp10	0.017375761	down	0.76308	6.11578
Metap2	0.017524526	down	1.32222	13.41963
Smcr8	0.01761683	down	0.15399	2.7189
Tmem198b	0.017696014	down	0.07087	1.71824
Asah2	0.017699363	down	0.20817	2.8685
Nipa2	0.018020023	down	1.26664	11.05084
Mpdz	0.018143882	down	0.54648	4.54117
Agpat5	0.019250698	down	1.34288	11.97937
Pitpnm3	0.019593861	down	3.07198	13.79765
Gstz1	0.019841188	down	0.06898	2.26115
Trim46	0.020109642	down	0.3521	6.20538
Rfc5	0.021102029	down	0	1.0105
Pkn2	0.021246011	down	0.13625	1.94463
Foxred2	0.021411385	down	0.24239	3.04688
Zfp955a	0.022432036	down	0.50184	6.6803
Nfs1	0.022704557	down	1.10965	10.84493
Aspa	0.023436961	down	0.28229	6.02307
AC126641.1	0.023458869	down	5.41702	21.17789
Sapcd1	0.023582184	down	0	1.46477
Pdp1	0.023914654	down	3.10894	18.94274
Asph	0.023938631	down	0.02304	1.76503
Inpp5b	0.025244566	down	0.63404	5.83428
RT1-M6-1	0.025411592	down	0	1.39157
Tp53i3	0.025411592	down	0	1.05292
Bahd1	0.02632403	down	1.78116	10.66683
Mast1	0.026413893	down	11.42125	34.89765
Abcc9	0.026553151	down	0.12014	1.55706
LOC100911837	0.026773769	down	0.67105	5.54078
AABR07033657.1	0.026829959	down	0.01742	1.26741
Fam149a	0.027505606	down	0.07372	1.15158
Ttl5	0.02797459	down	0.28608	3.24261
Uhrf2	0.028000146	down	1.94511	11.91478
Kat6a	0.028077163	down	0.12025	1.40597
Lonp2	0.028132924	down	4.1626	21.86294
Appl1	0.028462213	down	2.40511	15.84452
Kmt2e	0.028736822	down	1.29572	7.74995
Phod3	0.029647076	down	0.42146	4.02402
Klc1	0.030014673	down	8.93338	39.14595
Atp2c1	0.03036085	down	0.05056	1.07326
Dennd1a	0.030744571	down	1.44173	9.13596
Tnrc6c	0.030750065	down	0.50614	4.10257
Jam2	0.030762411	down	2.08768	11.79228
Med26	0.031968852	down	0.23051	2.7328
Lat2	0.032022091	down	0	1.02167
Grin2d	0.032451156	down	0.68688	5.10366
Tnr	0.033524598	down	0.29882	3.02575
Gpx4	0.033697383	down	18.59184	80.80704
AABR07032135.1	0.034279796	down	0.02858	1.85702
Plekho1	0.034301793	down	0.12401	2.2551
Rbfox2	0.035124014	down	0.07562	1.49111
Wasf1	0.03514338	down	0.33549	3.58343
Ankrd46	0.03606857	down	0.46227	4.90661
AABR07066379.1	0.036545294	down	0.05048	1.35865
Akt1	0.036737661	down	1.98567	12.89791
Plek	0.03674903	down	0.1208	1.94771
Pnodh1	0.038004856	down	1.49551	10.9513
Zfp276	0.038124058	down	0.52759	5.08914
Wdr18	0.038212532	down	0.42911	7.38692
Spire1	0.039758442	down	0.20724	2.0607
Csnk1d	0.039969241	down	0.09412	1.62651
Snrpa1	0.040179255	down	0.77405	8.43193
LOC108348098	0.040329819	down	0.01948	1.17124
Camk2b	0.042126704	down	0.52734	4.28754
RGD621098	0.042636126	down	0.30419	3.6666
Pomp	0.043075573	down	0.38797	5.50531
Ccp110	0.043849509	down	0.48565	3.75085
Psmb4	0.043890212	down	26.65065	98.66546
LOC108348180	0.044020758	down	0.0922	1.22853
LOC108351137	0.044519172	down	19.10969	74.27121
Mtss1l	0.045056508	down	9.02984	30.15209
Asap1	0.045487739	down	0.3218	3.09526
LOC108349244	0.046489628	down	0.27253	2.54273
Kif1b	0.046490551	down	0.97726	5.53407
Letmd1	0.047110031	down	2.73308	14.8193
Harbi1	0.047690909	down	0.27325	2.90928
Cttnbp2	0.048737227	down	1.31764	6.70414
Fam172a	0.049746681	down	0	1.91255
Incenp	0.049867587	down	0.43562	3.66411
Slc1a3	4.07212E-41	up	151.11305	0.01837
Prpf8	3.43483E-24	up	26.70323	0
Gabbr1	3.65755E-23	up	176.41099	1.8211
Dmxl2	2.49266E-19	up	17.558	0.0119
Slc9a6	1.11723E-18	up	26.89067	0
Gpx4	1.67013E-17	up	116.83944	0
AABR07070043.1	4.89669E-15	up	157.33983	0.55295
Impad1	6.89849E-15	up	13.7667	0
Ryr2	3.74537E-14	up	5.45926	0
L1cam	1.32514E-13	up	14.95248	0
Igf2	5.58271E-12	up	17.25282	0
Hpca	1.13758E-11	up	240.40057	8.86113
Rps19	1.26691E-11	up	120.91633	0
Dlg3	1.82227E-11	up	22.7122	0.03023
Ap2b1	1.95196E-11	up	10.29924	0
Arpc5l	3.19112E-11	up	42.53452	0
Chchd10	3.62753E-11	up	70.28348	0
Ube2v2	7.81062E-11	up	15.45125	0
Dync1li2	8.64499E-11	up	13.00225	0.00324
E2f1	1.18615E-10	up	27.54185	0
Prrc2a	2.80344E-10	up	6.65978	0
Prrc2a	3.47848E-10	up	24.4635	0.33513
Rgs14	3.8192E-10	up	15.5678	0
Sccpdh	5.3791E-10	up	23.26509	0
Golga4	8.69267E-10	up	8.90734	0.01366
Mcm3ap	1.10968E-09	up	9.77864	0.01352
Atrx	2.10617E-09	up	7.86987	0.03247
Hnrnpab	2.21711E-09	up	29.22428	0
Sorcs2	3.54257E-09	up	8.67648	0.00948
Psme4	7.28568E-09	up	7.05375	0.00252
Ap2b1	7.56405E-09	up	9.74078	0
Idh3g	8.93046E-09	up	63.80711	0.42498
Flnb	1.01259E-08	up	3.70536	0
Sms	1.20701E-08	up	19.907	0
Btaf1	1.3347E-08	up	3.96523	0
Iars	1.70298E-08	up	7.1348	0
Ube3c	1.70854E-08	up	14.13845	0.01767
Tlk1	2.22145E-08	up	8.04725	0
Cryzl1	2.52227E-08	up	16.25479	0
Edrf1	3.21324E-08	up	5.5534	0
Tas1r2	4.22293E-08	up	8.65811	0
Olfm3	6.29965E-08	up	6.50487	0
Prox1	7.01922E-08	up	3.63207	0
LOC108348078	9.39306E-08	up	11.78534	0
Thoc7	1.04777E-07	up	19.60371	0
Hbp1	1.42539E-07	up	9.02724	0
Exph5	1.5164E-07	up	2.6299	0
Retsat	2.26887E-07	up	21.50152	0.0878
Nav1	3.21889E-07	up	3.91574	0
Nfs1	3.60672E-07	up	15.19824	0
Grm4	4.37128E-07	up	9.15086	0
Macf1	4.52302E-07	up	1.03773	0.00059
LOC103689945	4.64067E-07	up	3.70601	0
Ipo4	6.33276E-07	up	20.33504	0.28506
Zmym6	7.45765E-07	up	4.52749	0
Echs1	1.0438E-06	up	11.82098	0
Tmem178b	1.05386E-06	up	9.40899	0.19935
Bicral	1.11073E-06	up	4.41703	0
Ube2a	1.36827E-06	up	12.11393	0.00815
Rgl2	1.61174E-06	up	5.70711	0
Sfmbt1	2.10234E-06	up	5.11488	0
Rpl26	2.3339E-06	up	13.69444	0
Casp8ap2	2.3816E-06	up	2.2557	0
Nexmif	2.47839E-06	up	2.54368	0.00189
Prtfdc1	2.85334E-06	up	13.50647	0
LOC103692166	2.98641E-06	up	3.35776	0
Sirpa	3.13462E-06	up	27.18816	0.90773
Agpat4	3.70126E-06	up	9.3191	0
Kif1b	4.12561E-06	up	2.17545	0
Adgrl4	4.19038E-06	up	3.24109	0
Mapk9	4.3184E-06	up	25.87087	0.80985
Hnrnpab	4.34579E-06	up	9.38471	0
Dhrs7b	4.34579E-06	up	10.45477	0
Ubtd2	4.36849E-06	up	5.06763	0
Furin	4.38641E-06	up	5.46828	0.01308
Zfhx4	4.73158E-06	up	1.6546	0.004
Pcnx1	4.92753E-06	up	1.65959	0
Trak1	5.05931E-06	up	3.46172	0.00291
Sipa1l1	5.12486E-06	up	14.85861	0.20998
Tp53	6.02758E-06	up	7.22451	0
LOC100363116	6.22435E-06	up	6.14881	0
Sall2	6.56797E-06	up	2.4005	0
Smpdl3b	6.78399E-06	up	5.02347	0
Rplp2	6.82074E-06	up	35.48387	0
Pot1	6.93229E-06	up	5.21409	0
AABR07035479.1	7.16138E-06	up	4.86232	0
LOC108349244	8.1638E-06	up	2.80842	0
Gars	8.72163E-06	up	4.94151	0
Npat	8.86751E-06	up	1.89938	0
Neurl1	9.1682E-06	up	48.42593	4.12831
Kat14	1.06016E-05	up	3.22812	0
Nrp2	1.09582E-05	up	5.79842	0.05254
Kdm5b	1.18056E-05	up	1.84454	0
Zfp280d	1.30867E-05	up	3.21279	0
Nsrp1	1.36259E-05	up	3.14553	0
Adgrl1	1.43423E-05	up	2.827	0.00501
Sh3bp5l	1.46084E-05	up	3.53515	0
Nsmce1	1.48661E-05	up	7.17271	0
Hyal2	1.48661E-05	up	5.50544	0
Oplah	1.5093E-05	up	3.8892	0.00697
Gpr155	1.54479E-05	up	3.22174	0.0058
Romo1	1.59489E-05	up	25.19237	0
LOC103689965	1.66209E-05	up	1.88146	0
LOC103689920	1.68187E-05	up	2.09821	0
Cntrl	1.81505E-05	up	2.59783	0.0092
LOC103690013	1.84988E-05	up	12.63621	0
Klhl4	1.86099E-05	up	2.68087	0
Klc4	1.88343E-05	up	4.03101	0
Adcy6	1.88343E-05	up	1.66302	0
Gabra5	1.92923E-05	up	4.40912	0
Senp5	1.9526E-05	up	4.30047	0
Ankib1	2.0743E-05	up	1.61209	0
Slx4ip	2.08693E-05	up	6.53494	0
Pds5b	2.08693E-05	up	2.18948	0
Pwp1	2.20459E-05	up	4.57269	0
LOC100910838	2.27317E-05	up	2.42075	0
St3gal1	2.31195E-05	up	5.08009	0.03959
NEWGENE_13081	2.32977E-05	up	7.3999	0
Oxr1	2.45493E-05	up	3.33992	0.00661
Tef	2.5163E-05	up	31.18851	2.04283
AABR07018038.1	2.70475E-05	up	6.16936	0
Gsptl1	2.7218E-05	up	3.50479	0
Eif3k	2.76651E-05	up	13.24815	0.01584
Shroom2	2.89642E-05	up	12.91207	0.5849
Jarid2	2.89915E-05	up	2.07451	0
Filip1	2.91759E-05	up	1.71155	0
Fbxw9	3.06285E-05	up	5.95774	0.0073
Pla2g6	3.1286E-05	up	6.45176	0.03142
Fam50a	3.16997E-05	up	6.47106	0
Mtif2	3.1904E-05	up	3.23499	0
Kcng4	3.47006E-05	up	2.05917	0
Mitf	3.56167E-05	up	1.68027	0
Wdr54	3.60957E-05	up	12.11614	0.02608
Slc5a6	3.82902E-05	up	3.62155	0.00468
Srr	3.87917E-05	up	4.00693	0
AABR07058985.2	4.03661E-05	up	2.62694	0
Pgm2l1	4.31565E-05	up	1.91927	0
Rmnd5a	4.73431E-05	up	11.74044	0.03566
Rrn3	4.76958E-05	up	5.67468	0.03252
Dyrk1a	4.90964E-05	up	2.68461	0
Kbtbd11	5.25241E-05	up	11.99227	0.28209
Ankrd6	5.72828E-05	up	2.81796	0.00893
Ddx17	6.03977E-05	up	18.84162	0.06044
LOC102552640	6.13909E-05	up	4.55401	0
Yif1b	6.59479E-05	up	3.57226	0
Klhl8	6.59479E-05	up	2.22774	0
RGD1309621	6.59479E-05	up	1.46791	0
Adcyap1r1	6.64251E-05	up	1.12336	0
Dctn1	6.71949E-05	up	2.95589	0.00972
Wdr41	6.78812E-05	up	2.46288	0
Letmd1	6.93749E-05	up	5.1678	0
Prima1	6.98814E-05	up	2.00295	0
Nt5c3b	7.12074E-05	up	10.69936	0.07585
Syt6	7.46416E-05	up	1.67635	0
Tpx2	8.03871E-05	up	2.33017	0
Synj2	8.44502E-05	up	1.85002	0.00292
Pex5l	8.76987E-05	up	5.43736	0.01904
AABR07035375.1	9.07098E-05	up	4.29042	0
Apopt1	9.42539E-05	up	10.86793	0
Snrpa1	9.42539E-05	up	5.67786	0
Brd9	0.000101412	up	5.17395	0.02258
AABR07029963.1	0.000105093	up	2.28588	0
Ntrk3	0.000105923	up	2.42441	0
March8	0.000107607	up	1.45542	0
Eml1	0.000113757	up	2.53073	0
C3	0.00011841	up	1.16626	0
Cp	0.000121312	up	1.80188	0
Aldh1l1	0.000122298	up	2.14785	0
Fancm	0.000131624	up	1.83613	0
Slfn13	0.000136044	up	1.68018	0
Rpl17	0.000137747	up	85.06232	2.6263
Ldirad4	0.000142815	up	2.18644	0.00661
Hmg20b	0.000151655	up	3.80347	0
Fibcd1l1	0.000155544	up	3.93758	0
Npepo	0.000159549	up	1.9617	0
Kcna3	0.000167333	up	5.37277	0.04792
Pitpnb	0.000167922	up	6.5006	0
Caap1	0.000172299	up	3.47974	0
Pop1	0.000176809	up	1.5489	0
Hipk2	0.000178342	up	1.07274	0
LOC108348052	0.000178342	up	3.10123	0
Fam3c	0.000183262	up	23.23029	0.88421
Lca5	0.000187877	up	1.50297	0
Uchl3	0.000189523	up	5.58341	0
Afap1	0.000192867	up	1.7577	0
Taf15	0.000199763	up	3.35809	0
Vps37c	0.000208795	up	4.65641	0
Sec31a	0.000211197	up	1.87207	0.0063
Arid1b	0.000212539	up	1.32089	0
AABR07062481.1	0.000230727	up	4.15227	0.00874
NEWGENE_13062	0.000234584	up	4.34541	0
LOC103690141	0.00024329	up	10.83308	0.16789
Dhx36	0.000244719	up	11.92866	0.64243
AABR07028989.1	0.000245481	up	2.46391	0
Sec22a	0.000245481	up	2.86627	0
St6galnac6	0.000261407	up	15.0162	0.40082
Hus1	0.000264181	up	1.0229	0
Jakmip2	0.000264181	up	1.77804	0
Mtx1	0.000269116	up	3.13932	0
Zfp689	0.000276725	up	1.86005	0
Slco1a4	0.000280932	up	8.61499	0.14831
Heph	0.000306914	up	1.02077	0
Zfp449	0.000318862	up	1.62083	0
Pus7	0.000325046	up	1.73182	0
Snph	0.000329394	up	10.78913	0.46036
LOC100911456	0.000331376	up	2.34797	0
Gtf2ird1	0.000334597	up	1.48728	0
Proser3	0.000337287	up	1.6744	0.00396
Rtl8a	0.000337641	up	47.00767	0.5378
Jak3	0.000351284	up	1.10638	0
Gstz1	0.000352812	up	8.68544	0.09183
Steap2	0.000354742	up	1.18589	0
Ddx5	0.000379227	up	77.02868	11.2645
Inpp1	0.00038015	up	1.90401	0
Cfb	0.000383959	up	1.57036	0
Sorbs1	0.000387816	up	1.03184	0
Nup54	0.000394785	up	2.37256	0.0059
Mme	0.000451849	up	1.27168	0
Tmem63c	0.000456569	up	1.04504	0
RT1-CE4	0.000481121	up	2.34867	0
LOC100911374	0.000486228	up	3.3334	0
Zfp213	0.000501966	up	1.51477	0
Cbln3	0.000501966	up	1.86129	0
Rbfox1	0.000509117	up	3.58258	0.047
Arhgap17	0.000512815	up	1.13537	0
Dock6	0.000529651	up	2.16782	0.02872
ST7	0.000559303	up	1.66501	0
Zfp414	0.000563013	up	10.99967	0.12448
LOC498453	0.000565485	up	1.47533	0
Akap13	0.000578149	up	1.00036	0.0114
Zgpat	0.000581832	up	14.27335	0.38844
AABR07031193.1	0.000611297	up	1.25537	0
Tox2	0.000667912	up	6.09991	0.07584
LOC103690054	0.000685373	up	3.63829	0
Wdr73	0.000726788	up	2.09026	0
Atrip	0.000735459	up	1.18934	0
Map2k5	0.000744265	up	1.3891	0
Ppig	0.000762902	up	1.14328	0.00355
Kcnh6	0.000771526	up	1.1054	0
Pitpnb	0.000780902	up	1.85097	0
Emp1	0.000790429	up	1.15414	0
Cyp2t1	0.000809945	up	1.88866	0
Tcf25	0.000825547	up	20.15225	1.26559
P2ry14	0.000872438	up	1.70343	0
Eng	0.000891609	up	1.7396	0.00917
Tjap1	0.00090948	up	3.71033	0.04005
Emp3	0.000917681	up	3.96356	0
Sgsm1	0.000930751	up	25.99321	3.82027
Nrxn2	0.000959555	up	16.60638	1.92889
Asph	0.000960095	up	1.61917	0
Ska2	0.000991583	up	2.3576	0
Zfp161	0.001049009	up	2.8679	0.03858
Plch1	0.001067297	up	2.03035	0.03337
Pecr	0.00118139	up	1.21893	0
Zfp653	0.001133516	up	1.23407	0
Gmfg	0.001165138	up	4.63965	0
Parp9	0.001167406	up	1.52783	0.00885
Tgif1	0.00118198	up	1.88903	0
Ube2j2	0.00118613	up	4.79502	0.04726
Abcc5	0.001315322	up	11.88429	1.02461
NEWGENE_13062	0.001323271	up	3.43114	0
Ahdc1	0.001378925	up	9.60324	0.7293
Rcbtb1	0.001382463	up	1.49433	0
Homer1	0.00146717	up	1.13133	0
Phf1	0.001526957	up	7.43019	0.20478
Cckbr	0.001535445	up	2.12583	0
Iqcd	0.001559176	up	1.70537	0
Spsb2	0.001559176	up	1.89424	0
Ncoa3	0.00157031	up	1.10675	0.00907
Pdp1	0.001605999	up	18.51446	1.27961
Slc4a3	0.001620639	up	5.19832	0.1836
Zfp385a	0.001660567	up	21.5099	1.48919
Kcnma1	0.001667596	up	3.12263	0.06166
Pdlim7	0.001685762	up	1.34237	0
Pprc1	0.001688413	up	2.74873	0.06489
Katnal1	0.001697718	up	1.50906	0
Kif1b	0.001829342	up	14.38412	2.36608
LOC108348175	0.001952225	up	2.09581	0
Man2c1	0.001963568	up	1.2257	0.00861
Kcnip1	0.002142737	up	2.11254	0.00976
Vegfb	0.002163558	up	12.51601	0.46836
Plek	0.002201533	up	1.23192	0
LOC103690026	0.002201533	up	2.35172	0
AABR07049085.1	0.002206276	up	1.29143	0.01399
Ctns	0.002221482	up	1.4979	0.01105
LOC501110	0.00224304	up	1.87286	0.01387
Cabin1	0.002268616	up	1.87461	0.04799
Pip4k2c	0.002298733	up	17.98517	1.3537
Fosl1	0.002321961	up	1.20154	0
Abi1	0.002321961	up	1.12068	0
Amot	0.002344564	up	4.48005	0.23968
Zfp787	0.002364131	up	3.77663	0
Nudt5	0.002407369	up	2.98396	0
Midn	0.002488975	up	14.13464	1.07114
Gnb3	0.002591727	up	1.15245	0
Ankrd11	0.002601033	up	13.76452	2.22877
LOC100911692	0.002691295	up	1.17845	0
Pced1b	0.00274788	up	2.07315	0.02094
Phldb2	0.002796244	up	1.10777	0
LOC680910	0.002850853	up	1.59173	0
NEWGENE_13081	0.002906951	up	1.51006	0
Ece1	0.002972105	up	2.98091	0.08657
Mical2	0.002972989	up	8.36318	0.38163
Micu2	0.002997193	up	1.14977	0.00609
Efemp1	0.003101424	up	3.36028	0.06085
Prpf39	0.003132845	up	1.90057	0.02348
Dock11	0.00314965	up	1.85927	0.05236
Chd4	0.003193797	up	1.10478	0.02117
Nat1	0.003211694	up	1.02801	0
Arpc2	0.003277908	up	3.74067	0
Camk2g	0.003277908	up	1.0066	0
Nsmaf	0.003470565	up	3.56029	0.09007
Spata2L	0.003478667	up	17.80658	1.67998
Drosha	0.00358084	up	19.17028	2.6115
Selenoi	0.003679966	up	1.2328	0.00712
Fastkd3	0.003732802	up	1.17106	0.0068
Slc22a5	0.003783327	up	2.7588	0.06469
Spp1	0.003968774	up	1.12119	0
Topbp1	0.004033892	up	1.26646	0.02251
Podxl	0.004088616	up	2.94152	0.11094
Reln	0.004295634	up	3.18227	0.2288
LOC100910708	0.004545839	up	1.71597	0
Mpp3	0.004727859	up	11.60416	0.81515
Ktn1	0.004731824	up	3.32674	0.13815
Nipsnap2	0.004873926	up	3.08042	0.03931
Fbxo9	0.004956064	up	42.53571	6.53323
Cyp1b1	0.004996658	up	1.03697	0
Cdk1	0.00511893	up	1.22317	0
Nup43	0.005245358	up	1.18024	0
Tuba1c	0.005310152	up	87.34896	17.54283
LOC687399	0.00537612	up	1.19794	0
Sgk1	0.005474848	up	1.26807	0.017
Mpst	0.005565136	up	16.47786	0.85917
Prss22	0.005651407	up	1.09709	0
Mdm1	0.005677227	up	1.28706	0.02245
Trafd1	0.005800353	up	17.6456	1.66778
Stx16	0.006148377	up	17.03223	2.4155
Stmn1	0.006227565	up	112.2474	21.73234
Arpc2	0.006683487	up	1.72545	0.02016
Mlc1	0.00668505	up	83.54815	23.56062
Spata2	0.006962128	up	3.1604	0.09925
Gabra6	0.006986466	up	3.27868	0.10965
Tmem164	0.007007991	up	9.32576	1.04006
AABR07039304.1	0.00706017	up	15.07261	0.31212
Senp1	0.007161889	up	1.32187	0
Ppp1cc	0.007433893	up	2.19735	0.03365
Stard6	0.007570809	up	1.09599	0
Nrcam	0.007574197	up	1.0105	0.03273
LOC100911516	0.007805162	up	51.17524	4.58602
Mycbpap	0.008328973	up	1.65483	0.04863
Hnrnpf	0.008420696	up	1.54467	0.03187
Recql	0.008422775	up	1.19264	0.01542
Plcxd2	0.008551692	up	1.01754	0.01325
Nrxn1	0.008566617	up	9.43727	0.65821
Neto1	0.008903127	up	15.00759	1.895
Cdv3	0.009009225	up	1.01682	0
Pi16	0.009105578	up	1.9269	0.04449
Grm3	0.009273123	up	1.25746	0.02927
Specc1	0.009417534	up	1.31295	0.05528
Cdr2l	0.009928488	up	8.27095	0.70177
Fcgr1a	0.010384362	up	1.24302	0.01116
Gkap1	0.010686252	up	1.18478	0.01702
AC127784.2	0.010686252	up	5.79303	0.08323
Ckap5	0.010847978	up	3.05825	0.1974
Grin2a	0.011784217	up	3.004	0.17022
Orc5	0.012183744	up	2.65583	0.10327
R3hdm1	0.01299014	up	30.88139	8.03438
Jcad	0.013021811	up	3.423	0.22606
Traip	0.013350023	up	3.25849	0.14595
Spns2	0.013530835	up	4.62581	0.33872
Ralbp1	0.013872575	up	15.53297	2.33606
Epb41l3	0.01387984	up	24.5885	5.60985
Anapc11	0.014018599	up	1.47293	0.0551
Pelp1	0.014384683	up	14.50991	2.15016
Postn	0.014764745	up	1.43022	0.05318
Gpr4	0.01502738	up	5.59576	0.29006
Foxk2	0.015335299	up	4.49496	0.36781
AABR07042937.1	0.015422523	up	3.69486	0
Elp5	0.015435471	up	12.10839	0.98006
Bmp3	0.015798568	up	1.49325	0.0772
Ntrk2	0.015849619	up	54.46272	20.51298
Rffl	0.015900898	up	1.3045	0.0501
Kcnip1	0.016234562	up	4.24013	0.20821
Fam19a5	0.016270809	up	32.24144	6.54959
Inpp4a	0.016414002	up	20.35763	3.73002
Hipk4	0.016553977	up	7.57731	0.61626
Fgf13	0.016618417	up	5.56137	0.39771
Vcam1	0.017120196	up	5.33077	0.38683
Tmem18	0.017638741	up	12.43866	1.57389
Scamp3	0.017876851	up	4.08776	0.23786
Mettl18	0.018257787	up	1.01518	0.01858
Atp2b1	0.018341045	up	4.26262	0.36263
Smndc1	0.018452451	up	3.86841	0.18853
Mrgbp	0.018602408	up	1.37909	0
Mdk	0.018602408	up	1.17634	0
Wnk1	0.019813711	up	1.60502	0.1368
Tmbim6	0.019961245	up	2.98326	0.10494
Zfp777	0.020514144	up	8.34726	0.89218
LOC100912483	0.021136636	up	4.41741	0.10892
Ndrg2	0.021258796	up	15.15564	1.85378
Nkapd1	0.021346072	up	2.56942	0.16694
Exoc1	0.021412924	up	12.05337	1.61121
Wfdc6a	0.021699439	up	1.05139	0
Smim8	0.021699439	up	1.37983	0
Adam11	0.021704755	up	2.27218	0.16824
Scaf8	0.021866028	up	4.71612	0.4505
Ntng2	0.022032578	up	6.90015	0.59484
Trappc8	0.022292384	up	9.65044	1.61123
LOC103690018	0.022348466	up	6.59125	0.44876
Hddc2	0.022597844	up	4.59707	0.26226
Cc2d1a	0.024347565	up	7.25221	0.81171
LOC100359687	0.024438248	up	5.52173	0.32574
LOC100911576	0.024473872	up	23.87215	3.53973
Incenp	0.024479075	up	3.15856	0.23427
Gatad2a	0.025082976	up	2.39491	0.19355
Ssr1	0.025338048	up	4.59144	0.26811
Mapk9	0.025371405	up	9.66017	1.39453
Mfhas1	0.025779524	up	1.15835	0.05705
Spred1	0.02621337	up	3.20917	0.18759
Lsm14a	0.026345934	up	2.96419	0.21108
AABR07055844.2	0.027389653	up	6.34725	0.34318
Bok	0.027479259	up	1.37308	0
Cabin1	0.027689013	up	3.38697	0.41275
Nabp1	0.027882863	up	2.65041	0.17136
Uck2	0.028182418	up	11.95687	0.9677
LOC691918	0.028614645	up	1.55409	0
Yipf6	0.028614645	up	1.21911	0
Kat2a	0.029326757	up	29.23934	7.33308
Mpv17l	0.029368996	up	2.36876	0.08768
Pigc	0.029461219	up	5.29152	0.27797
Banp	0.02975825	up	2.58071	0.16597
LOC100910750	0.029781968	up	1.48546	0.10466
Alox5	0.030657676	up	1.66955	0.09525
Naglu	0.030889443	up	2.63762	0.18717
Magi1	0.031019469	up	0.96745	0.05213
Zfp467	0.031217484	up	2.9717	0.19526
Ankzf1	0.03129004	up	8.91851	1.12954
Eps15l1	0.031443388	up	9.09852	1.28483
AABR07007642.1	0.03158067	up	6.37209	0.67326
Slc6a6	0.031857309	up	1.55835	0.09057
Myo5b	0.032012502	up	5.97471	1.09444
Fcgrt	0.032050152	up	5.92634	0.4947
Cttn	0.032078396	up	17.06914	3.25732
RGD1359158	0.032772635	up	2.16483	0.11377
AABR07027088.1	0.033116649	up	12.41504	0.87512
Tab2	0.033278235	up	1.71284	0.13716
Tcea2	0.034027666	up	27.73928	4.34358
Ctnna1	0.034776351	up	26.85671	7.35855
Tfrc	0.034892105	up	8.58855	1.52608
Sdha	0.03490256	up	97.16115	36.52833
Pacsin3	0.035181578	up	8.99684	0.99242
AABR07030521.1	0.035526202	up	5.46026	0.58608
Dlgap4	0.037120213	up	8.28312	1.22198
Pcdha6	0.037165938	up	1.62372	0.1434
Gbf1	0.03732444	up	7.14138	1.37467
Tmco4	0.037959787	up	1.0451	0.06081
Dlg2	0.038655559	up	14.3753	2.49781
Cad	0.039103891	up	2.22616	0.25265
Sorcs3	0.039493386	up	2.16258	0.2154
Cox7a2l	0.039654111	up	1.87403	0.1016
Slc6a6	0.039708033	up	6.48407	0.74547
Mrps11	0.040688342	up	16.22862	1.85991
Kalrn	0.040802823	up	4.68553	0.89447
Ddx39a	0.04146756	up	2.36819	0.16923
RGD1309730	0.0423569	up	1.48101	0.06589
Smarca2	0.042909911	up	1.86258	0.18237
Nf1	0.042937278	up	6.30082	1.42774
Dync1li2	0.04375799	up	4.25538	0.29478
Rtn4	0.044612048	up	1.26153	0.08355
Spock1	0.044900997	up	3.95178	0.34252
Kifc3	0.045722813	up	1.4479	0.11737
Flt3lg	0.046409969	up	1.37285	0.06401
Tug1	0.047031327	up	4.22473	0.62071
Car4	0.047117795	up	6.99996	0.72813
Smc6	0.047554302	up	12.90352	2.84014
Yap1	0.049022694	up	5.44927	0.5507
Kcne2	0.049470188	up	1.37306	0.08932
